# Furostanol and Spirostanol Saponins from *Tribulus terrestris*

**DOI:** 10.3390/molecules21040429

**Published:** 2016-03-30

**Authors:** Zhen-Fang Wang, Bing-Bing Wang, Yang Zhao, Fang-Xu Wang, Yan Sun, Rui-Jie Guo, Xin-Bo Song, Hai-Li Xin, Xin-Guang Sun

**Affiliations:** 1 Department of Pharmaceutical Care, PLA General Hospital, Beijing 100850, China; wangzhenfangok@126.com (Z.-F.W.); syshirley0986@163.com (Y.S.); guoruijie5683@163.com (R.-J.G.); 2 Tianjin Zhongyi Pharmaceutical Co. Ltd., Tianjin University of Traditional Chinese Medicine, Tianjin 300193, China; manager@tjzyzy.com; 3 Department of Biology, Beijing Institute of Radiation Medicine, Beijing 100850, China; wbbyzu0401@126.com (B.-B.W.); mmyzhao@163.com (Y.Z.); wangfangxu5@163.com (F.-X.W.)

**Keywords:** *Tribulus terrestris*, steroidal saponins, terrestrinins J–U, structure identification

## Abstract

Twelve new steroidal saponins, including eleven furostanol saponins, terrestrinin J–T (**1**–**11**), and one spirostanol saponin, terrestrinin U (**12**), together with seven known steroidal saponins **13**–**19** were isolated from *T. terrestris*. The structures of the new compounds were established on the basis of spectroscopic data, including 1D and 2D NMR and HRESIMS, and comparisons with published data.

## 1. Introduction

**Figure 1:**
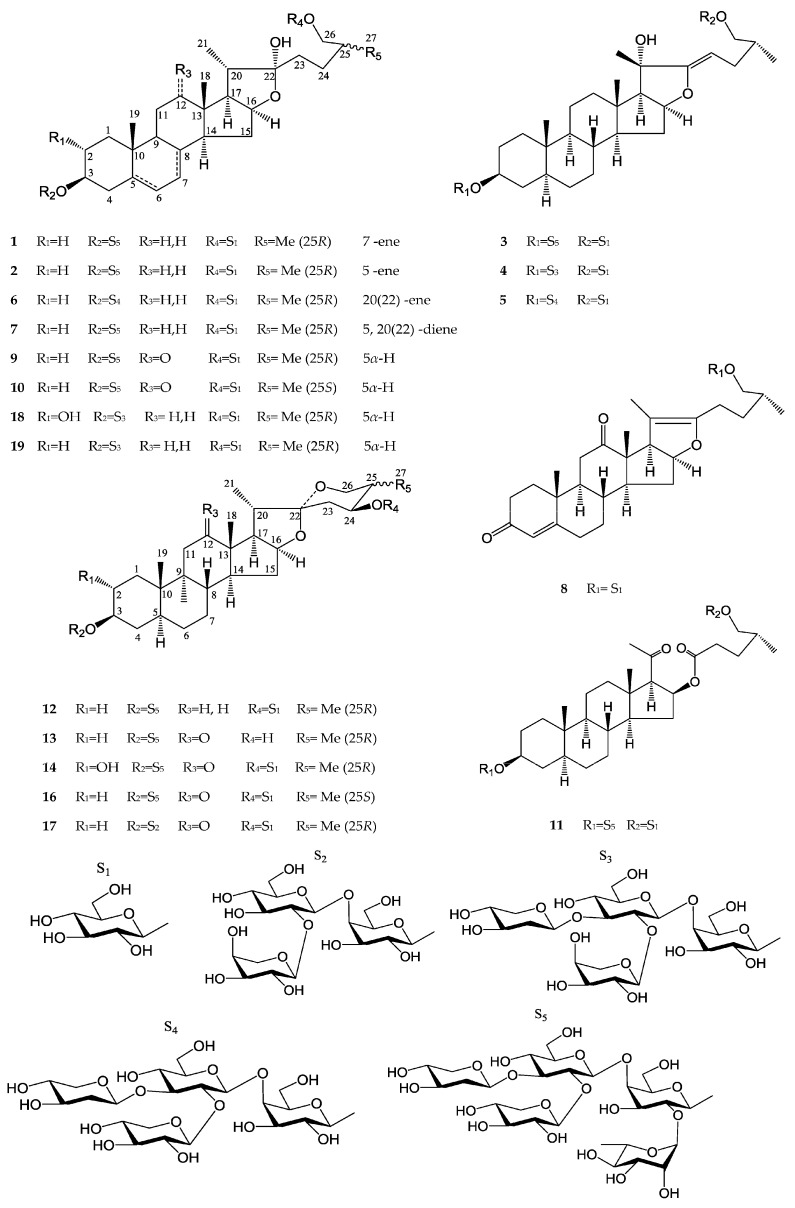
Steroidal saponins **1**–**19** isolated from *Tribulus terrestris*.

*Tribulus terrestris* L. is a perennial plant widely distributed around the world, especially in subtropical areas. Its dried fruit, named “Jili” in Chinese, has been used as a traditional Chinese medicine (TCM) for the treatment of edema, abdominal distention, emission, morbid leucorrhea and vitiligo [1]. Additionally, *T. terrestris* (the fruit or the whole plant) can not only act as an aphrodisiac tonic and an antibacterial agent [2,3], but is also used for the treatment of cardiovascular diseases [4,5]. Previous phytochemical studies have reported a number of saponins and alkaloids from this plant, and several studies have demonstrated that saponins are responsible for the biological activities of *T. terrestris* [1,5,6,7,8,9,10,11,12]. In this phytochemical investigation focused on the steroidal saponins of this plant, eleven new furostanol saponins **1**–**11** and one new spirostanol saponin (**12**), together with seven known steroidal saponins **13**–**19** were isolated. Their structures (Figure 1) were elucidated by extensive analysis of mass spectrometry, and 1D and 2D NMR spectroscopy data, as well as comparisons with published data.

## 2. Results and Discussion

The fresh whole plant of *T. terrestris* was extracted using 70% aq. EtOH. The extract was subjected to macroporous resin SP825 column chromatography to afford two saponin-rich fractions. These fractions were subsequently separated on silica-gel, MCI silica-gel, ODS silica-gel and semi-preparative HPLC to provide twelve new steroidal saponins, named terrestrinins J–U (**1**–**12**), which were identified by NMR techniques and HRESIMS, and seven known steroidal saponins **13**–**19** that were identified as by comparison of their NMR and MS data with those reported in the literature as (25*R*)-3β-hydroxy-5α-spirostan-12-one 3-*O*-β-d-xylopyranosyl-(1→2)-[β-d-xylopyranosyl-(1→3)]-β-d-glucopyranosyl-(1→4)-[α-l-rhamnopyranosyl-(1→2)]-β-d-galactopyran-oside (**13**) [5], (25*R*)-26-[(β-d-glucopyranosyl)oxy]-5α-furostane-3β,22α-diol 3-*O*-α-l-rhamno-pyranosyl-(1→2)-[β-d-glucopyranosyl-(1→4)]-β-d-galactopyranoside (**14**) [13], (25*R*)-26-[(β-d-glucopyranosyl)oxy-5α-furost-20(22)-en-3β-ol 3-*O*-β-d-xylopyranosyl-(1→3)-[β-d-xylopyranosyl-(1→2)]-β-d-glucopyranos-yl (1→4)-[α-l-rhamnopyranosyl(1→2)]-β-d-galactopyranoside (**15**) [14], 25*S*-terrestrosin I (**16**) [15], 25*R*-terrestrosin I (**17**) [15], parvispinoside A (**18**) [16], and parvispinoside B (**19**) [16].

**Figure 2:**
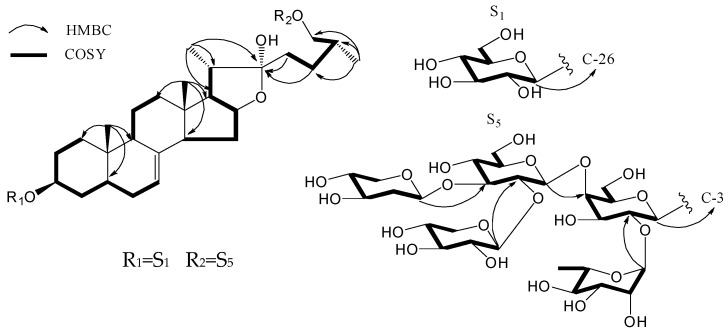
Key COSY and HMBC correlations for **1**.

Compound **1** was isolated as a white powder and its molecular formula was determined to be C_61_H_100_O_31_ by the HRESIMS [M − H]^−^ ion peak at *m*/*z* 1327.6162 (calcd. 1327.6170). The ^1^H-NMR spectrum of **1** showed two methyl singlets at δ 0.76 (3H, s, Me-18) and 0.87 (3H, s, Me-19), two methyl doublets at δ 1.31 (3H, d, *J* = 7.2 Hz, Me-21) and 0.97 (3H, d, *J* = 6.6 Hz, Me-27), and one olefinic proton at δ 5.09 (br s, H-7). Additionally, it also showed six anomeric proton signals at δ 4.83 (1H, d, *J* = 7.8 Hz, H-1′), 6.20 (1H, s, H-1′′), 4.98 (1H, d, *J* = 7.8 Hz, H-1′′′), 5.23 (1H, d, *J* = 7.8 Hz, H-1′′′′), 5.42 (1H, d, *J* = 7.8 Hz, H-1′′′′′), and 4.81 (1H, d, *J* = 7.8 Hz, H-1′′′′′′), which indicated that **1** contains six sugar moieties. The ^13^C-NMR spectrum exhibited 61 carbons, including two olefinic carbons at δ 118.8 (C-7) and 139.0 (C-8). The ^1^H-^1^H COSY correlations for δ 5.09 (H-7)/1.67 (H-6a) and δ 2.08 (H-6b)/1.22 (H-5), as well as the HMBC correlations (Figure 2) between δ 0.87 (H-19) and δ 40.3 (C-5), 139.0 (C-8), and 49.3 (C-9) indicated the presence of a double bond between C-7 and C-8.The α orientation of the C-22 hydroxy group of the aglycone moiety was deduced from the hemiketal carbon signal at δ 110.7 [17], and was further confirmed by the ROESY correlation between H-20 (δ 2.19) and H-23 (δ 2.03). The chemical shift difference between the two proton signals of H_2_-26 (Δδ_H_ = 0.32 ppm < 0.48 ppm) demonstrated the 25*R* configuration of **1** [18].

The above data assignments for the aglycone moiety of **1** were supported by ^1^H-^1^H COSY, HMBC, and HSQC experiments (Figures S3–S5). Furthermore, ROESY correlations between H-5/H-9, H-3, and H-14/H-17, Me-21, as well as the absence of ROESY correlations between Me-18/Me-19, H-20 indicated that the H-5 is α-oriented and A/B, C/D, and D/E ring junctions are *trans*, *trans*, and *cis*, respectively. Accordingly, the aglycone moiety of **1** was deduced to be (25*R*)-5α-furost-7-ene-3β,22α,26-triol. The absolute configurations of the sugar units, glucose, galactose, rhamnose and xylose, were identified to be d (glucose, galactose, and xylose) and l (rhamnose), respectively, by GC analysis. Furthermore, the proton spin systems and the carbon resonances of each sugar were fully assigned by ^1^H-^1^H COSY, HSQC, and HMBC spectra of **1**.

The large coupling constants (^3^*J*_1,2_ > 7 Hz) were consistent with the β-configuration of glucose, galactose, and xylose [19,20,21], while the carbon signals for δ 72.5 (C-3′′) and δ 69.3 (C-5′′) provided evidence for α-configuration of rhamnose [19]. The sugar sequence and its linkage to the aglycone were ascertained by long-range correlations between δ 6.20 (H-1′′) and δ 76.5 (C-2′), δ 4.98 (H-1′′′) and δ 81.3 (C-4′), δ 5.23 (H-1′′′′) and δ 87.6 (C-3′′′), δ 5.42 (H-1′′′′′) and δ 81.5 (C-2′′′′), δ 4.83 (H-1′) and δ 77.0 (C-3), and δ 4.81 (H-1′′′′′′) and δ 75.3 (C-26) in the HMBC spectrum (Figure 2). Thus, compound **1** was elucidated as (25*R*)-26-[(β-d-glucopyranosyl)oxy]-5α-furost-7-ene-3β,22α-diol 3-*O*-β-d-xylo-pyranoseyl-(1→2)-[β-d-xylopyranosyl-(1→3)]-β-d-glucopyranosyl-(1→4)-[α-l-rhamnopyranosyl-(1→2)]-β-d-galacto-pyranoside, which was named terrestrinin J.

Compound **2** was obtained as a white amorphous powder with the same molecular formula as **1**, C_61_H_100_O_31_, as determined by HRESIMS (*m*/*z* 1327.6230 [M − H]^−^). Comparison of the NMR data of **2** with those of **1** (Table 1 and Table 2) indicated that the structure of **2** is similar to that of **1**, expect the B ring. The signals at δ 141.0 (C-5), 121.7 (C-6) and δ 5.28 (H-6) indicated the double bond at C-5 and C-6 in **2**. Thus, the aglycone of **2** was identified as (25*R*)-furost-5-ene-3β,22α,26-triol. The whole structure of **2** was finally confirmed by the 1D and 2D NMR experiments which indicated the structure of **2** to be (25*R*)-26-[(β-d-glucopyranosyl)oxy]-furostan-5-ene-3β,22α*-*diol 3-*O*-β-d-xylo-pyranosyl-(1→2)-[β-d-xylopyranosyl-(1→3)]-β-d-glucopyranosyl-(1→4)-[α-l-rhamnopyranosyl-(1→2)]-β-d-galactopyranoside, named terrestrinin K.

The molecular formula of **3** was determined to be C_61_H_100_O_31_ by HRESIMS ([M − H]^−^ *m*/*z* 1327.6205, calcd. 1327.6170). The NMR data of compound **3** were very similar to those of a known compound, (20*S*,25*R*)-26-[(β-d-glucopyranosyl)oxy]-furost-5,22-diene-3β,20α-diol 3-*O*-β-d-gluco-pyranosyl-(1→4)-[*a*-l-rhamnopyra-nosyl-(1→2)]-β-d-glucopyranoside, reported in the literature [14], which indicated that they share a similar aglycone, except for the loss of a double bond at C-5 and C-6. The HMBC correlations (See Figure S17) of H-17/C-18, C-20, C-22, H-21/C-17, C-20, C22, H-23/C-20, C-22, and H-24/C-17, C-22, C-23, C-25, were observed, indicating the double bond at C-22 and C-23 and the hydroxyl group at C-20, respectively. The ROESY (See Figure S19) spectrum showed correlations between Me-21 and H-23 and between Me-21 and Me-18 indicated the hydroxyl group at C-20 to be in the α orientation. Additionally, the ROESY correlations between δ 1.22 (H-5) and 1.56 (H-9) revealed the α-orientation of H-5. Further, comparison the NMR data of sugar units between **3** and **1** revealed that they had same sugar moiety, which was confirmed by the combined analyses of ^1^H-^1^H COSY, HSQC, and HMBC spectra of **3**. On the basis of the above evidence, **3** was elucidated as (20*S*,25*R*)-26-[(β-d-glucopyranosyl)oxy]-5α-furost-22-ene-3β,20α-diol 3-*O*-β-d-xylopyranosyl-(1→2)-[β-d-xylopyranos-yl-(1→3)]-β-d-glucopyranosyl-(1→4)-[α-l-rhamnopyranosyl-(1→2)]-β-d-galactopyranoside, named terrestrinin L.

Compound **4** was isolated as a white amorphous powder with the molecular formula C_56_H_92_O_28_ (HRESIMS, [M − H]^−^ at *m*/*z* 1211.5687). Comparison of the MS and NMR data of **4** with those of **3** suggested that the structure of **4** is similar to that of **3**, with one fewer sugar unit in the sugar chain linked to C-3 of the aglycone. The sugar sequence of glucose, galactose, xylose and its linkage to C-3 and C-26 of the aglycone were ascertained by correlations between δ 4.91 (H-1′) and δ 77.4 (C-3), δ 5.18 (H-1′′) and δ 79.6 (C-4′), δ 5.47 (H-1′′′) and δ 81.1 (C-2′′), δ 5.07 (H-1′′′′) and δ 85.6 (C-3′′), and δ 4.82 (H-1′′′′′) and δ 75.3 (C-26) in the HMBC spectrum. Thus, the structure of **4** was concluded to be (20*S*,25*R*)-26-[(β-d-glucopyranosyl)oxy]-5α-furost-22-ene-3β,20α-diol 3-*O*-β-d-galactopyranosyl-(1→2)-[β-d-xylopyranosyl-(1→3)]-*O*-β-d-glucopyranosyl-(1→4)-β-d-galactopyranoside, named terrestrinin M.

The molecular formula of **5** was deduced as C_56_H_92_O_28_ due to the appearance of a [M − H]^−^ ion at *m*/*z* 1211.5709 in the HRESIMS, as same as **4**. According to the comparison of the ^1^H and ^13^C-NMR data of **5** with that of **4**, they were deduced to share the same aglycone moiety. Further comparison of the NMR data of **5** with those of polianthoside D [21,22] suggested they have the same sugar moiety. The HMBC correlations between δ 5.19 (H-1′′) and δ 79.9 (C-4′), δ 5.56 (H-1′′′) and δ 81.4 (C-2′′), δ 5.23 (H-1′′′′) and δ 86.8 (C-3′′), and δ 4.82 (H-1′′′′′) and δ 75.3 (C-26), as well as between δ 4.88 (H-1′) and δ 77.4 (C-3), revealed the positions of glycosylations and the sugar sequence. In conclusion, the structure of **5** was concluded to be (20*S*,25*R*)-26-[(β-d-glucopyranosyl)oxy]-5α-furost-22-ene-3β,20α-diol 3-*O*-β-d-glucopyranosyl-(1→2)-[β-d-xylopyranosyl-(1→3)]-*O*-β-d-glucopyranosyl-(1→4)-β-d-galactopyranoside and named terrestrinin N.

Compound **6** showed an [M − H]^−^ ion peak at *m*/*z* 1195.5730 (Calcd. 1195.5748) in the negative HRESIMS, corresponding to a molecular formula of C_56_H_92_O_27_. The ^13^C-NMR spectrum showed two olefinic carbon signals at δ 103.7 (C-20) and 152.4 (C-22), which indicated that a double bond existed in **6**. In the HMBC spectrum (See Figure S36), the correlations of H-17/C-20, C22, H-21/C-17, C-10, C-22, H-23/C-20, C-22, C-24, C-25, and H-24/C-22 indicated that the double bond is located between C-20 and C-22. Comparison NMR data of **6** with those of terrestroside A [8], revealed that they had the same aglycone structure. Further comparison of the NMR data of **6** with those of **5** suggested they have the same sugar moiety. The sugar sequence and its linkage sites were ascertained by the HMBC correlations. Thus, the structure of **6** was elucidated as (25*R*)-26-[(β-d-glucopyranosyl)oxy]-5α-furost-20(22)-ene-3β-ol 3-*O*-β-d-glucopyranosyl-(1→2)-[β-d-xylopyranosyl-(1→3)]-*O*-β-d-glucopyranosyl-(1→4)-β-d-galactopyranoside, named terrestrinin O.

On the basis of HRESIMS (*m*/*z* 1193.5640), compound **7** showed the same molecular formula, C_56_H_90_O_27_, as polygodoside G [23]. Likewise, the two compounds showed almost identical NMR data, with the only difference between **7** and polygodoside G being their respective 25*R* and 25*S* configuration. The chemical shift value between H_2_-26 geminal protons (Δδ_H_ < 0.48 ppm), suggested a 25*R* configuration for **7**. Therefore, compound **7** was elucidated to be (25*R*)-26-[(β-d-gluco-pyranosyl)oxy]-furosta-5,20(22)-dien-3β-ol 3-*O*-β-d-glucopyranosyl-(1→2)-[β-d-xylopyranosyl-(1→3)]-*O*-β-d-glucopyranosyl-(1→4)-β-d-galactopyranoside, and named terrestrinin P.

The HR ESI-MS of **8** showed an [M + HCOO]^−^ ion at *m*/*z* 633.3265 (Calcd. 633.3275), suggesting a molecular formula of C_33_H_48_O_9_. The ^1^H-NMR spectrum of **8** (Table 3) showed an anomeric proton signals at δ 4.81 (1H, d, *J* = 7.8 Hz, H-1′), four methyl group signals at δ 0.99, 1.08, 1.75, and 1.02 (each 3H, Me-18, -19, -21, and -27), and one olefinic proton signal at δ 5.85, s, H-4). The ^13^C-NMR spectrum showed two double bond signals at δ 124.8 (C-4) and 168.5 (C-5), and δ 103.1 (C-20) and 153.3 (C-22), and two characteristic ketone carbons at δ 197.9 (C-3) and 211.8 (C-12). The NMR data of **8** were quite similar to those of terrestrinin A [24], with the only evident difference being the geminal signals for H_2_-26, which indicated that the difference between their structures was the configuration of C-25. According to the H_2_-26 signals at δ 3.62 (1H, dd, *J* = 6.0, 9.6 Hz, 26-Ha) and 3.95 (1H, o, 26-Hb), the configuration of C-25 was identified to be *R* [25]. Hence, **8** was assigned to be (25*R*)-26-[(β-d-glucopyranosyl)oxy]-furosta-4,20(22)-diene-3,12-dione, named terrestrinin Q.

Compound **9** was isolated as a white amorphous powder with a molecular formula of C_61_H_100_O_32_, which was determined by the negative ion HRESIMS (*m*/*z* 1343.6113 [M − H]^−^). Comparison of the ^1^H and ^13^C-NMR data of **9** (Table 1) with those of polianthoside D [22] and **1** revealed that **9** and polianthoside D shared the same aglycone, and **9** and **1** shared the same sugar moiety. Thus, **9** was elucidated as (25*R*)-26-[(β-d-glucopyranosyl)oxy]-3β,22α-dihydroxy-5α-furostan-12-one 3-*O*-β-d-xylopyranosyl-(1→2)-[β-d-xylopyranosyl-(1→3)]-β-d-glucopyranosyl-(1→4)-[α-l-rhamnopyranosyl-(1→2)]-β-d-galacto-pyranoside, and named terrestrinin R.

Compound **10** displayed an [M − H]− ion at *m*/*z* 1343.6145 (Calcd. 1343.6119) by HRESIMS, giving a molecular formula of C_61_H_100_O_32_, which was identified as a isomer of **9** (Table 1 and Table 3). A detailed comparison the NMR data of **10** with **9**, suggested that **10** contained the same chains and almost identical aglycone moiety as **9**. The only difference was the *S* configuration (H_2_-26: Δδ_H_ = 0.58 ppm) of C-25 in **10** rather than an *R* configuration in **9** [18,25]. Thus, the structure of **10** was assigned as (25*S*)-26-[(β-d-glucopyranosyl)oxy]-3β,22α-dihydroxy-5α-furostan-12-one 3-*O*-β-d-xylopyranosyl-(1→2)-[β-d-xylopyranosyl-(1→3)]-β-d-glucopyranosyl-(1→4)-[α-l-rhamnopyranosyl-(1→2)]-β-d-galactopyranoside, named terrestrinin S.

HRESIMS of **11** gave a [M − H]^−^ ion at *m*/*z* 1343.6128, indicating a molecular formula of C_61_H_100_O_32_. In the ^13^C-NMR spectrum, two carbonyl group signals at δ 205.5 (C-20) and 173.2 (C-22) were observed in **11**. The key COSY and HMBC correlations (See Figures S66–S67) observed for **11** indicated that two carbonyl groups were present at C-20 and C-22. Comparison of the NMR data of **11** with those of diodcresides A [26] and **1**, indicated that **11** had the same aglycone as diodcresides A, and the same sugar moiety as **1**. The small difference in chemical shift values of the geminal protons H_2_-26 indicated a 25*R* configuration [18] (Δδ_H_ = 0.39 ppm) for **11**. Additionally, the α-configuration of H-16 was determined from the *J* value of 7.8 Hz between H-16 and H-17. Based on these data, the structure of **11** was characterized as 16β-{(25*R*)-26-[(β-d-glucopyranosyl)oxy]-25-methylpentanoyloxy}-3β-hydroxy*-*5α-pregnan-20-one 3-*O*-β-d-xylopyranosyl-(1→2)-[β-d-xylopyranosyl-(1→3)]-β-d-gluco-pyranosyl-(1→4)-[α-l-rhamnopyranosyl-(1→2)]-β-d-galactopyranoside, named terrestrinin T.

Compound **12** had the molecular formula of C_61_H_100_O_31_, as established by HRESIMS (*m*/*z* 1327.6173 [M − H]^−^). The ^1^H-NMR spectrum displayed four steroidal methyl groups at δ 0.72 (3H, s, Me-18), 0.83 (3H, s, Me-19), 1.05 (3H, d, *J* = 6.6 Hz, Me-21), and 1.13 (3H, d, *J* = 6.6 Hz, Me-27) , and six anomeric proton signals at δ 4.84 (1H, d, *J* = 7.8 Hz, H-1′), 6.18 (1H, s, H-1′′), δ 4.97 (1H, d, *J* = 7.8 Hz, H-1′′′), 5.23 (1H, d, *J* = 7.8 Hz, H-1′′′′), 5.42 (1H, d, *J* = 7.8 Hz, H-1′′′′′), and 4.92 (1H, d, *J* = 7.8 Hz, H-1′′′′′′), as well as six anomeric carbon signals at δ 100.2, 102.0, 105.4, 105.1, 105.8 and 106.4. The sugar units and their configurations was identified by GC analysis after acidic hydrolysis, as well as the large coupling constants. Furthermore, the NMR data of **12** were similar to those of a known spirostanol saponin, 25R-tribulosin [27], with an additional sugar group at the aglycone. The additional sugar group positioned at C-24 was confirmed by the long-range correlations between H-1′′′′′′ (δ 4.92) and C-24 (δ 81.6) in the HMBC spectrum (See Figure S72). Additionally, the ROSEY correlations (See Figure S74) from H-23ax to H-24 and H-20, and from H-26ax to H-24 and H-16, the *J* value of 12.6 Hz (H-24/H-23ax), and chemical shifts of C-23 (δ 40.9) suggested the 24S configuration of **12** [7,12,28]. On the basis of the foregoing evidence, the structure of **12** was established as (25*S*)-24-[(β-d-glucopyranosyl)oxy]-5α-spirost-3β-ol 3-O-β-d-xylopyranosyl-(1→2)-[β-d-xylopyranosyl-(1→3)]-β-d-glucopyranosyl-(1→4)-[α-l-rhamnopyranosyl-(1→2)]-β-d-galactopyranoside, named terrestrinin U.

## 3. Experimental Section

### 3.1. General Information

Optical rotations were obtained on a 341 digital polarimeter (Perkin-Elmer, Waltham, MA, USA). IR and UV spectra were recorded on FTIR-8400S (Shimadzu Corp., Tokyo, Japan) and UV2550 spectrometer (Shimadzu Corp.), respectively. HRESIMS were recorded on Synapt Q/TOF MS (Waters Corp., Milford, MA, USA). NMR spectra (^1^H at 600 MHz and ^13^C at 150 MHz) were taken on a UNITY INOVA 600 spectrometer (Varian, Palo Alto, CA, USA) in pyridine-*d*_5_ solution, and the chemical shifts are given in ppm on the δ scale with tetramethylsilane (TMS) as an internal standard. HPLC separations were performed using a Waters 2695 series instrument equipped with an analytical Venusil XBP C18 column (250 × 4.6 mm, 5 μm, Agela Technologies, Tianjin, China), a YMC (250 × 10 mm, 5 μm, Kyoto, Japan) preparative column, and ELSD 2000 evaporative light-scattering detector (Alltech, Lexigton, KY, USA). ODS gel (50 µm, YMC), Sephadex LH-20 (Pharmacia, Uppsala, Sweden), and MCI gel (CHP 20P, 75–150 μm, Mitsubishi Chemical Corporation, Tokyo, Japan) were used for column chromatography. TLC was carried out on silica gel GF254 (Yantai Chemical Inst., Yantai, China) plates, and spots were visualized under UV light (254 or 365 nm) or by spraying with 10% H_2_SO_4_ in 95% EtOH followed by heating.

### 3.2. Plant Material

The fresh whole plant of *T. terrestris* was collected from Beijing, China in July 2013, and authenticated by Prof. Bao-Lin Guo (Institute of Medicinal Plant Development, Chinese Academy of Medical Science and Peking Union Medical College). A voucher specimen (No. 20130726) has been deposited at the Herbarium of the Beijing Institute of Radiation Medicine, Beijing, China.

### 3.3. Extraction and Isolation

The fresh whole plant (20 kg) of *T. terrestris* was cut into small pieces and refluxed with 75% (*v*/*v*) EtOH (40 L × 1 h, 2 times). The combined extract was evaporated under reduced pressure and then separated by column chromatography (CC) on macroporous resin SP825 eluting with 5%, 60% and 90% EtOH in succession. The 60% EtOH eluate fraction was separated on a silica-gel column using gradient solvents of CHCl_3_–MeOH–H_2_O (15:1:0.01, 9:1:0.01 and 2:1:0.01, *v*/*v*) to give five fractions (Fr. A-E). Fr. B were subjected to ODS CC eluted with MeCN–H_2_O (20:86, *v*/*v*) and semi-preparative HPLC with MeCN–H_2_O (19:81, *v*/*v*) to yield **16** (4.7 mg) and **17** (21.1 mg). Fr. C was subjected to column chromatography (CC) over MCI silica gel eluted with EtOH–H_2_O (25:75 to 55:45, *v*/*v*) to give five subfractions (Fr. C_1_ to Fr. C_3_). Fr. C_2_ was subjected to ODS CC (20% MeCN in H_2_O) to give three subfractions Fr. C_2-1_ to Fr. C_2-3_. Fr. C_2-2_ was purified by ODS semi-preparative HPLC (MeCN–H_2_O, 25:75, *v*/*v*) to afford **18** (54.5 mg). Fr. C_2-3_ was further purified by semi-preparative HPLC eluted with MeCN–H_2_O (22: 78, *v*/*v*) to yield **4** (16.0 mg) and **5** (15.3 mg). Fr. C_3_ was separated on an MCI-gel column with Me_2_CO–H_2_O (32:68, *v*/*v*) and was purified by semi-preparative HPLC with Me_2_CO-H_2_O (29:71) to yield **19** (110.8 mg). Fr. D was fractioned by ODS chromatography (23%–30% MeCN in H_2_O) to give three subfractions (Fr. D_1_ to Fr. D_3_). Fr. D1 was further purified successively by ODS semi-preparative HPLC (MeCN–H_2_O, 22:78, *v*/*v*) to yield **10** (10.4 mg) and **9** (70.5 mg). Fr. D_2_ was purified by semi-preparative HPLC with MeCN–H_2_O (24:76, *v*/*v*) to yield **3** (4.8 mg). Fr. D_3_ was separated by an ODS column eluted with MeOH–H_2_O (28:72, *v*/*v*), and then was purified by semi-preparative HPLC with MeCN–H_2_O (24:76, *v*/*v*) to yield **11** (5.8 mg), **1** (4.4 mg), and **2** (5.0 mg). Fr. E was subjected to ODS silica-gel CC with a gradient mixture of MeCN-H_2_O, (32:68 to 40:60, *v*/*v*) as elute, and three fractions were obtained (Fr. E_1_ to Fr. E_3_). Fr. E_1_ was purified by semi-preparative HPLC with MeCN-H_2_O (32:68) to yield **12** (60.7 mg), **6** (125.4 mg), and **7** (17.0 mg), and Fr. E_2_ was purified by semi-preparative HPLC with MeCN-H_2_O (35:75) to yield **13** (9.3 mg), **8** (7.4 mg), and **15** (81.1 mg), respectively. Fr. E_2_ was purified successively by semi-preparative HPLC using MeCN-H_2_O (34:66, *v*/*v*) as mobile phase to afford **14** (6.2 mg).

*Terrestrinin J* (**1**): White amorphous powder, ${\left[\alpha \right]}_{D}^{20}$−62.4 (*c* 0.067, MeOH), HR-ESI-MS (neg.): *m*/*z* 1327.6162 [M − H]^−^ (Calcd for C_61_H_99_O_31_, 1327.6170), ESI-MS (pos.): *m*/*z* 1311.6 [M + H − H_2_O]^+^, 1179.6 [M + H − H_2_O − 132]^+^, 1017.5 [M + H − H_2_O − 132 − 162]^+^ , 885.5 [M + H − H_2_O − 2 × 132 − 162]^+^, 739.4 [M + H − H_2_O − 2 × 132 − 162 − 146]^+^, 577.4 [M + H − H_2_O − 2 × 132 − 2 × 162 − 146]^+^, 415.3 [M + H − H_2_O − 2 × 132 − 3 × 162 − 146]^+^, ^13^C-NMR (pyridine-*d*_5_) and ^1^H-NMR (pyridine-*d*_5_) data see Table 1 and Table 2.

*Terrestrinin K* (**2**): White amorphous powder, ${\left[\alpha \right]}_{D}^{20}$−64.5 (*c* 0.071, MeOH), HR-ESI-MS (neg.): *m*/*z* 1327.6230 [M − H]^−^ (Calcd for C_61_H_99_O_31_, 1327.6170), ESI-MS (pos.): *m*/*z* 1311.64 [M + H − H_2_O]^+^, 1179.6 [M + H − H_2_O − 132]^+^, 885.5 [M + H − H_2_O − 2 × 132 − 162]^+^, 739.4 [M + H − H_2_O − 2 × 132 − 162 − 146]^+^, 577.4 [M + H − H_2_O − 2 × 132 − 2 × 162 − 146]^+^, 415.3 [M + H − H_2_O − 2 × 132 − 3 × 162 − 146]^+^, ^13^C-NMR (pyridine-*d*_5_) and ^1^H-NMR (pyridine-*d*_5_) data see Table 1 and Table 2.

*Terrestrinin L* (**3**): White amorphous powder, ${\left[\alpha \right]}_{D}^{20}$−35.8 (*c* 0.059, MeOH), HR-ESI-MS (neg.): *m*/*z* 1327.6105 [M − H]^−^ (Calcd for C_61_H_99_O_31_, 1327.6170), ESI-MS (pos.): *m*/*z* 1311.6 [M + H − H_2_O]^+^, 1149.6 [M + H − H_2_O − 162]^+^, 1017.6 [M + H − H_2_O − 162 − 132]^+^, 885.5 [M + H − H_2_O − 162 − 2 × 132]^+^, 723.4 [M + H − H_2_O − 2 × 162 − 2 × 132]^+^, 577.4 [M + H − H_2_O − 2 × 162 − 2 × 132 − 146]^+^, 415.3 [M + H − H_2_O − 3 × 162 – 2 × 132 − 146]^+^, ^13^C-NMR (pyridine-*d*_5_) and ^1^H-NMR (pyridine-*d*_5_) data see Table 1 and Table 2.

*Terrestrinin M* (**4**): White amorphous powder, ${\left[\alpha \right]}_{D}^{20}$−31.3 (*c* 0.064, MeOH), HR-ESI-MS (neg.): *m*/*z* *m*/*z* 1211.5687 [M − H]^−^ (Calcd for C_56_H_91_O_28_, 1211.5697), ESI-MS (pos.): *m*/*z* 1195.57 [M + H − H_2_O]^+^, 1033.52 [M + H − H_2_O − 162]^+^, 901.48 [M + H − H_2_O − 162 − 132]^+^, 739.43 [M + H − H_2_O − 2 × 162 − 132]^+^, 577.37 [M + H − H_2_O − 3 × 162 − 132]^+^, 415.32 [M + H − H_2_O − 4 × 162 − 132]^+^, ^13^C-NMR (pyridine-*d*_5_) and ^1^H-NMR (pyridine-*d*_5_) data see Table 1 and Table 2.

*Terrestrinin N* (**5**): White amorphous powder, ${\left[\alpha \right]}_{D}^{20}$−26.0 (*c* 0.065, MeOH), HR-ESI-MS (neg.): *m*/*z* 1211.5709 [M − H]^−^ (Calcd for C_56_H_91_O_28_, 1211.5697), ESI-MS (pos.): *m*/*z* 1195.6 [M + H − H_2_O]^+^, 1033.5 [M + H − H_2_O − 162]^+^, 901.5 [M + H − H_2_O − 162 − 132]^+^, 739.4 [M + H − H_2_O − 2 × 162 − 132]^+^, 577.4 [M + H − H_2_O − 3 × 162 − 132]^+^, 415.3 [M + H − H_2_O − 4 × 162 − 132]^+^, ^13^C-NMR (pyridine-*d*_5_) and ^1^H-NMR (pyridine-*d*_5_) data see Table 1 and Table 2.

*Terrestrinin O* (**6**): White amorphous powder, ${\left[\alpha \right]}_{D}^{20}$−43.3 (*c* 0.069, MeOH), HR-ESI-MS (neg.): *m*/*z* 1195.5730 [M − H]^−^ (Calcd for C_56_H_91_O_27_, 1195.5748), ESI-MS (pos.): *m*/*z* 1035.5 [M + H − 162]^+^, 741.4 [M + H − 2 × 162 − 132]^+^, 579.4 [M + H − 2 × 162 − 2 × 132]^+^, 417.3 [M + H − 3 × 162 − 2 × 132]^+^, ^13^C-NMR (pyridine-*d*_5_) and ^1^H-NMR (pyridine-*d*_5_) data see Table 1 and Table 2.

*Terrestrinin P* (**7**): White amorphous powder, ${\left[\alpha \right]}_{D}^{20}$−42.0 (*c* 0.067, MeOH), HR-ESI-MS (neg.): *m*/*z* 1193.5640 [M − H]^−^ (Calcd for C_56_H_89_O_27_, 1193.5591), ESI-MS (pos.): *m*/*z* 1033.5 [M + H − 162]^+^, 739.4 [M + H − 2 × 162 − 132]^+^, 577.4 [M + H − 2 × 162 − 2 × 132]^+^, 415.3 [M + H − 3 × 162 − 2 × 132]^+^, ^13^C-NMR (pyridine-*d*_5_) and ^1^H-NMR (pyridine-*d*_5_) data see Table 1 and Table 3.

*Terrestrinin Q* (**8**): White amorphous powder, ${\left[\alpha \right]}_{D}^{20}$+8.8 (*c* 0.069, MeOH), HR-ESI-MS (neg.): *m*/*z* 633.3265 [M + HCOO]^−^ (Calcd for C_34_H_49_O_11_, 633.3275), ESI-MS (pos.): *m*/*z* 427.29 [M + H − 162]^+^, ^13^C-NMR (pyridine-*d*_5_) and ^1^H-NMR (pyridine-*d*_5_) data see Table 1 and Table 3.

*Terrestrinin R* (**9**): White amorphous powder, ${\left[\alpha \right]}_{D}^{20}$−49.1 (*c* 0.077, MeOH), HR-ESI-MS (neg.): *m*/*z* 1343.6113 [M − H]^−^ (Calcd for C_61_H_99_O_32_, 1343.6119), ESI-MS (pos.): *m*/*z* 1327.7 [M + H − H_2_O]^+^, 1195.6 [M + H − H_2_O − 132]^+^, 901.5 [M + H − H_2_O − 2 × 132 − 162]^+^, 739.4 [M + H − H_2_O − 2 × 132 − 2 × 162]^+^, 593.4 [M + H − H_2_O − 2 × 132 − 2 × 162 − 146]^+^, 431.3 [M + H − H_2_O − 2 × 132 − 3 × 162 − 146]^+^, ^13^C-NMR (pyridine-*d*_5_) and ^1^H-NMR (pyridine-*d*_5_) data see Table 1 and Table 3.

*Terrestrinin S* (**10**): White amorphous powder, ${\left[\alpha \right]}_{D}^{20}$−43.5 (*c* 0.067, MeOH), HR-ESI-MS (neg.): *m*/*z* 1343.6145 [M − H]^−^ (Calcd for C_61_H_99_O_32_, 1343.6119), ESI-MS (pos.): *m*/*z* 1327.7 [M + H − H_2_O]^+^, 1195.6 [M + H − H_2_O − 132]^+^, 901.5 [M + H − H_2_O − 2 × 132 − 162]^+^, 739.4 [M + H − H_2_O − 2 × 132 − 2 × 162]^+^, 593.4 [M + H − H_2_O − 2 × 132 − 2 × 162 − 146]^+^, 431.3 [M + H − H_2_O − 2 × 132 − 3 × 162 − 146]^+^, ^13^C-NMR (pyridine-*d*_5_) and ^1^H-NMR (pyridine-*d*_5_) data see Table 1 and Table 3.

*Terrestrinin T* (**11**): White amorphous powder, ${\left[\alpha \right]}_{D}^{20}$−52.5 (*c* 0.067, MeOH), HR-ESI-MS (neg.): *m*/*z* 1343.6128 [M − H]^−^ (Calcd for C_61_H_99_O_32_, 1343.6119), ESI-MS (pos.): *m*/*z* 1213.60 [M + H − 132]^+^, 1051.6 [M + H − 132 − 162]^+^, 919.5 [M + H − 2 × 132 − 162]^+^, 757.4 [M + H − 2 × 132 − 2 × 162]^+^, 611.4 [M + H − 2 × 132 − 2 × 162 − 146]^+^, 449.3 [M + H − 2 × 132 − 3 × 162 − 146]^+^, ^13^C-NMR (pyridine-*d*_5_) and ^1^H-NMR (pyridine-*d*_5_) data see Table 1 and Table 3.

*Terrestrinin U* (**12**): White amorphous powder, ${\left[\alpha \right]}_{D}^{20}$−71.3 (*c* 0.067, MeOH), HR-ESI-MS (neg.): *m*/*z* 1327.6173 [M − H]^−^ (Calcd for C_61_H_99_O_31_, 1327.6170), ESI-MS (pos.): *m*/*z* 1149.6 [M + H − H_2_O − 162]^+^, 903.5 [M + H − H_2_O − 162 − 146]^+^, 723.4 [M + H − H_2_O − 2 × 162 − 2 × 132]^+^, 577.4 [M + H − H_2_O − 2 × 162 − 2 × 132 − 146]^+^, 415.3 [M + H − H_2_O − 3 × 162 − 2 × 132 − 146]^+^, ^13^C-NMR (pyridine-*d*_5_) and ^1^H-NMR (pyridine-*d*_5_) data see Table 1 and Table 3.

### 3.4. Acid Hydrolysis and Sugar Analysis

Isolates (2.0 mg each for compounds **1**–**12**) were individually hydrolyzed in 2N CF_3_COOH (5 mL) and heated at 95 °C for 5 h. After extraction with CH_2_Cl_2_ (5 mL) three times, the aqueous layer was repeatedly evaporated to dryness with EtOH until neutral and then the residue of the sugars in pyridine (1 mL) was added to l-cysteine methyl ester hydrochloride (3.0 mg), and the mixture was stirred at 60 °C for 1 h. Furthermore, HMDS-TMCS (hexamethyldisilazane–trimethylchlorosilane) (0.6 mL) was added and then kept at 60 °C for 0.5 h. Finally, the supernatant was analyzed by GC under the following conditions: 6890 gas chromatograph (Agilent Technologies, Santa Clara, CA, USA); HP-5 capillary column; column temperature: 180 °C/250 °C, programmed increase, 15 °C/min; 5973 mass spectrograph detector; carrier gas: N_2_ (1 mL/min); injection and detector temperature: 250 °C; injection volume: 1.0 µL, split ratio: 1/50. The derivatives of L-rhamnose, D-xylose D-glucose, and D-galactose were detected, with the following retention times, *t*_R_ (min): D-xylose (15.05 and 16.90), L-rhamnose (16.65 and 18.45), D-glucose (20.19 and 20.95), and D-galactose (20.80 and 22.48) compared the retention times with those of standard samples, respectively.

## 4. Conclusions

In conclusion, twelve new steroidal glycosides, including eleven furostanol saponins **1**–**11** and one spirostanol saponin **12**, were isolated from the fresh whole plant of *T. terrestris*, as well as seven known steroidal saponins **13**–**19**. Their structures were elucidated by extensive analysis of spectroscopic methods including 1D and 2D NMR experiments (HSQC, HMBC, COSY, and ROESY), and HRESIMS. Among them, the aglycone of compound **1** found in this study has a rare aglycone with an unsaturation between C-7 and C-8. In addition, compounds **13**, **16** and **17** were isolated as monomeric form for the first time. This work could be helpful to investigate on the bioactive compounds from *T. terrestris*.

## Figures and Tables

**Table 1:** ^13^C-NMR data of compounds **1**–**12** (δ in pyridine-*d*_5_).

Position	1	2	3	4	5	6	7	8	9	10	11	12
1	37.3	37.6	37.3	37.2	37.2	37.3	37.5	35.4	36.7	36.7	37.2	37.3
2	30.0	30.2	30.0	29.9	29.9	29.9	30.2	32.4	29.7	29.7	29.9	30.3
3	77.0	77.9	77.1	77.4	77.4	77.4	78.2	197.9	76.7	76.7	77.1	77.0
4	29.9	38.9	34.4	34.9	34.9	34.4	39.3	124.8	34.2	34.2	34.5	34.4
5	40.3	141.0	44.7	44.7	44.7	44.7	141.1	168.5	44.5	44.5	44.7	44.7
6	34.3	121.7	29.0	28.9	28.9	29.0	121.6	34.1	28.7	28.7	28.9	29.0
7	118.8	32.4	32.3	32.2	32.2	32.6	32.4	31.6	31.7	31.7	32.1	32.4
8	139.0	31.7	34.6	34.6	34.6	35.0	31.4	34.1	34.4	34.4	34.4	35.3
9	49.3	50.4	54.3	54.2	54.2	54.4	50.3	54.7	55.8	55.9	54.5	54.4
10	34.7	37.2	35.9	35.8	35.8	35.8	37.0	38.9	36.4	36.4	35.9	35.9
11	21.7	21.1	20.7	20.7	20.7	21.5	21.3	37.6	38.0	38.0	20.8	21.3
12	39.6	39.9	39.5	39.5	39.5	39.9	39.7	211.8	212.9	212.9	38.4	40.1
13	41.9	40.7	40.7	40.7	40.7	43.8	43.4	57.3	55.6	55.6	42.6	40.8
14	55.1	56.6	56.8	56.8	56.8	54.8	55.0	53.3	55.8	55.8	53.8	56.5
15	31.8	32.5	33.4	33.4	33.4	34.9	34.5	33.8	31.8	31.8	32.3	32.0
16	80.9	81.1	84.2	84.2	84.2	84.5	84.5	82.9	79.7	79.7	74.7	81.6
17	63.8	63.9	68.0	68.0	68.0	64.7	64.5	56.2	55.1	55.1	66.8	62.6
18	16.5	16.4	13.8	13.8	13.8	14.5	14.1	14.1	16.2	16.2	14.0	16.6
19	13.1	19.4	12.4	12.3	12.3	12.3	19.4	16.5	11.9	11.9	12.4	12.4
20	41.2	40.8	76.7	76.7	76.7	103.7	103.6	103.1	41.3	41.3	205.5	42.2
21	16.3	16.4	21.9	21.9	21.9	11.8	11.8	11.6	15.3	15.3	30.5	14.9
22	110.7	110.7	163.7	163.7	163.7	152.4	152.4	153.3	110.8	110.8	173.2	111.6
23	37.2	37.2	91.6	91.6	91.6	23.7	23.7	23.7	37.1	37.1	35.4	40.9
24	28.4	28.4	29.9	29.9	29.9	31.5	31.5	31.5	28.4	28.3	29.1	81.6
25	34.3	34.3	35.0	35.0	35.0	33.5	33.5	33.5	34.3	34.3	33.4	38.3
26	75.1	75.2	75.3	75.3	75.3	75.0	75.0	75.0	75.3	75.4	74.7	65.1
27	17.5	17.5	17.7	17.7	17.7	17.4	17.4	17.3	17.5	17.5	16.9	13.5
	**Gal**	**Gal**	**Gal**	**Gal**	**Gal**	**Gal**	**Gal**	**Glc**	**Gal**	**Gal**	**Gal**	**Gal**
1′	100.3	100.6	100.2	102.3	102.5	102.5	102.5	105.0	100.2	100.2	100.2	100.2
2′	76.5	76.4	76.6	73.1	73.2	73.2	73.2	75.2	76.6	76.6	76.6	76.6
3′	76.7	76.7	76.7	75.8	75.6	75.6	75.6	78.7	76.8	76.7	76.7	76.7
4′	81.3	81.3	81.3	79.6	79.9	79.9	79.9	71.8	81.3	81.3	81.3	81.4
5′	70.7	70.7	70.7	75.5	75.4	75.4	75.4	78.5	70.8	70.8	70.7	70.7
6′	60.4	60.3	60.4	60.5	60.7	60.7	60.6	62.9	60.5	60.5	60.4	60.5
	**Rha**	**Rha**	**Rha**	**Glc**	**Glc**	**Glc**	**Glc**		**Rha**	**Rha**	**Rha**	**Rha**
1′′	101.9	101.9	102.0	105.6	105.2	105.2	105.2		102.0	102.0	102.0	102.0
2′′	72.5	72.5	72.5	81.1	81.4	81.4	81.4		72.5	72.5	72.5	72.5
3′′	72.7	72.7	72.7	85.6	86.8	86.8	86.8		72.7	72.7	72.7	72.7
4′′	74.1	74.1	74.0	70.7	70.5	70.5	70.5		74.0	74.0	74.0	74.0
5′′	69.3	69.4	69.4	77.6	77.6	77.6	77.6		69.4	69.4	69.3	69.4
6′′	18.5	18.5	18.5	63.1	63.0	63.0	63.0		18.5	18.5	18.5	18.5
	**Glc**	**Glc**	**Glc**	**Gal**	**Glc**	**Glc**	**Glc**		**Glc**	**Glc**	**Glc**	**Glc**
1′′′	105.4	105.4	105.4	105.3	105.0	105.0	104.9		105.3	105.3	105.4	105.4
2′′′	81.5	81.5	81.5	73.8	76.3	76.3	76.3		81.5	81.5	81.5	81.5
3′′′	87.6	87.6	87.6	74.0	78.8	78.8	78.8		87.6	87.6	87.6	87.7
4′′′	70.4	70.4	70.4	70.5	71.1	71.1	71.1		70.4	70.4	70.4	70.4
5′′′	77.8	77.8	77.8	77.6	77.8	77.8	77.8		77.8	77.8	77.8	77.8
6′′′	62.9	62.9	62.9	62.7	62.5	62.5	62.5		62.9	62.9	62.9	62.9
	**Xyl**	**Xyl**	**Xyl**	**Xyl**	**Xyl**	**Xyl**	**Xyl**		**Xyl**	**Xyl**	**Xyl**	**Xyl**
1′′′′	105.1	105.1	105.1	104.9	104.9	104.9	105.0		105.1	105.1	105.1	105.1
2′′′′	75.2	75.1	75.1	75.0	75.1	75.1	75.1		75.1	75.1	75.1	75.1
3′′′′	78.8	78.8	78.8	78.5	78.7	78.7	78.7		78.8	78.8	78.8	78.8
4′′′′	70.7	70.7	70.7	70.7	70.8	70.8	70.8		70.8	70.8	70.7	70.7
5′′′′	67.4	67.4	67.4	67.3	67.4	67.4	67.4		67.4	67.4	67.4	67.4
	**Xyl**	**Xyl**	**Xyl**	**Glc**	**Glc**	**Glc**	**Glc**		**Xyl**	**Xyl**	**Xyl**	**Xyl**
1′′′′′	105.8	105.8	105.8	104.9	104.9	104.9	104.9		105.8	105.8	105.8	105.8
2′′′′′	75.8	75.8	75.8	75.4	75.4	75.2	75.2		75.8	75.8	75.8	75.8
3′′′′′	79.1	79.1	79.1	78.6	78.6	78.7	78.7		79.1	79.1	79.1	79.1
4′′′′′	70.9	70.9	70.9	71.8	71.8	71.8	71.8		70.9	70.9	70.9	70.9
5′′′′′	67.7	67.7	67.7	78.5	78.5	78.5	78.5		67.7	67.7	67.7	67.7
6′′′′′				62.9	62.9	62.9	62.9					
	**Glc**	**Glc**	**Glc**						**Glc**	**Glc**	**Glc**	**Glc**
1′′′′′′	105.0	105.0	104.9						105.0	105.1	104.9	106.4
2′′′′′′	75.3	75.3	75.4						75.2	75.2	75.2	75.7
3′′′′′′	78.6	78.6	78.6						78.7	78.7	78.6	78.6
4′′′′′′	71.7	71.8	71.8						71.8	71.8	71.7	71.8
5′′′′′′	78.5	78.5	78.5						78.5	78.5	78.5	78.0
6′′′′′′	62.9	62.9	62.9						62.9	62.9	62.9	62.9

**Table 2:** ^1^H-NMR data (*J* in Hz) of compounds **1**–**6** in pyridine-*d*_5_ (δ in ppm).

Position	1	2	3	4	5	6
1	0.93 m	0.94 m	0.80 m	0.79 m	0.78 m	0.78 m
	1.66 m	1.70 m	1.54 m	1.49 m	1.50 m	1.50 m
2	1.68 m	1.85 m	2.01 m	1.61 m	1.58 m	1.58 m
	2.02 m	2.06 m	2.76 m	2.04 m	2.03 m	2.03 m
3	3.90 m	3.87 m	3.91 m	4.03 m	4.09 m	4.03 m
4	1.69 m	2.72 m	1.66 m	1.37 m	1.33 m	1.34 m
	1.74 m	2.74 m	1.91 m	1.80 m	1.77 m	1.77 m
5	1.22 m	-	0.87 m	0.90 m	0.87 m	0.89 m
6	1.67 o	5.28 m	1.14 m	1.11 m	1.10 m	1.10 m
	2.08 o	-	1.16 m	1.13 m	1.12 m	1.12 m
7	5.09 br s	1.46 o	0.77 m	0.76 m	0.77 m	0.79 m
	-	1.84 d (15.0)	1.47 m	1.47 m	1.48 m	1.49 m
8	-	1.54 m	1.36 m	1.33 m	1.33 m	1.32 m
9	1.56 br s	0.87 m	0.45 m	0.45 m	0.44 m	0.48 m
10	-	-	-	-	-	-
11	1.44 m	1.40 m	1.19 m	1.16 m	1.16 m	1.19 m
	1.48 m	1.42 m	1.36 m	1.35 m	1.34 m	1.39 m
12	1.14 m	1.08 m	1.10 m	1.10 m	1.10 m	1.08 m
	1.70 o	1.72 m	1.84 m	1.83 m	1.84 m	1.68 m
13	-	-	-	-	-	-
14	1.89 m	1.04 m	0.91 m	0.91 m	0.90 m	0.80 m
15	1.71 o	1.44 o	1.44 m	1.45 m	1.45 m	1.44 m
	2.02 m	2.01 o	2.02 m	2.02 m	2.04 m	2.08 m
16	4.99 o	4.94 m	5.18 m	5.19 m	5.20 m	4.77 m
17	2.02 o	1.92 o	2.21 d (6.6)	2.20 d (6.6)	2.21 d (6.0)	2.42 d (10.2)
18	0.76 s	0.87 s	0.86 s	0.86 s	0.87 s	0.70 s
19	0.87 s	1.04 s	0.84 s	0.62 s	0.63 s	0.89 s
20	2.19 m	2.22 m	-	-	-	
21	1.31 d (7.2)	1.32 d (7.2)	1.71 s	1.71 s	1.72 s	1.63 s
22	-	-	-	-	-	-
23	2.01 m	2.01 m	4.48 o	4.49 o	4.50 o	2.21 m
	2.03 m	2.03 m	-	-	-	
24	1.66 m	1.66 m	2.27 m	2.28 m	2.28 m	1.46 m
	2.03 m	2.03 m	2.39 m	2.38 m	2.39 m	1.82 m
25	1.90 m	1.91 m	2.05 m	2.07 m	2.08 m	1.94 m
26	3.60 dd (6.0, 9.0)	3.61 dd (6.0, 9.0)	3.68 o	3.69 dd (6.0, 9.0)	3.68 o	3.62 dd (6.0, 9.0)
	3.92 m	3.93 m	4.02 o	3.97 o o	4.01 o	3.94 m
27	0.97 d (6.6)	0.98 d (7.2)	1.07 d (6.6)	1.08 d (6.6)	1.08 d (6.6)	1.04 d (7.8)
	**Gal**	**Gal**	**Gal**	**Gal**	**Gal**	**Gal**
1′	4.83 d (7.8)	4.82 d (7.8)	4.85 d (7.8)	4.91 d (7.8)	4.88 d (7.8)	4.88 d (7.8)
2′	4.48 o	4.49 o	4.47 o	4.34 t (7.2)	4.41 o	4.41 o
3′	4.14 o	4.12 o	4.14 o	4.09 o	4.12 o	4.12 o
4′	4.47 o	4.47 o	4.48 o	4.58 o	4.59 o	4.59 o
5′	4.11 o	4.11 o	4.11 o	4.02 m	4.02 m	4.02 m
6′	4.17 m	4.13 m	4.19 m	4.19 m	4.21 m	4.21 m
	4.69 m	4.68 m	4.69 dd (9.6, 15.6)	4.68 dd (10.2, 16.2)	4.69 t (10.2)	4.69 t (10.2)
	Rha	Rha	Rha	Gal	Glc	Glc
1′′	6.20 s	6.20 s	6.19 s	5.18 d (7.8)	5.19 d (7.8)	5.19 d (7.8)
2′′	4.75 o	4.76 br s	4.75 dd (5.4, 11.4)	4.55 o	4.42 dd (7.8, 16.8)	4.42 dd (7.8, 16.8)
3′′	4.54 o	4.57 br d (12.0)	4.54 o	4.14 o	4.16 t (9.0)	4.16 t (9.0)
4′′	4.23 o	4.23 o	4.23 o	3.76 t (10.2)	3.82 t (10.2)	3.82 t (10.2)
5′′	4.93 m	4.95 m	4.91 m	3.92 m	3.88 m	3.88 m
6′′	1.71 d (6.0)	1.72 d (6.6)	1.70 d (6.0)	4.00 o	4.03 o	4.03 o
				4.49 o	4.52 o	4.52 o
	**Glc**	**Glc**	**Glc**	**Glc**	**Glc**	**Glc**
1′′′	4.98 d (7.8)	4.98 d (7.8)	4.99 d (7.8)	5.47 d (7.8)	5.56 d (7.8)	5.56 d (7.8)
2′′′	4.27 t (7.8)	4.26 t (8.4)	4.27 t (8.4)	4.60 o	4.06 o	4.06 o
3′′′	4.06 o	4.06 o	4.04 o	3.88 o	4.06 o	4.06 o
4′′′	3.82 o	3.81 o	3.82 o	4.22 o	4.22 o	4.22 o
5′′′	3.83 m	3.82 m	3.82 m	3.92 m	3.89 m	3.89 m
6′′′	4.01 o	4.01 br d (10.8)	4.01 o	4.39 dd (6.0, 11.4)	4.36 o	4.36 o
	4.50 o	4.51 o	4.51 o	4.53 o	4.57 o	4.57 o
	**Xyl**	**Xyl**	**Xyl**	**Xyl**	**Xyl**	**Xyl**
1′′′′	5.23 d (7.8)	5.23 d (7.8)	5.23 d (7.8)	5.07 d (7.8)	5.23 d (7.8)	5.23 d (7.8)
2′′′′	3.93 m	3.94 o	3.96 o	3.93 o	3.95 o	3.95 o
3′′′′	4.08 o	4.08 o	4.08 o	3.93 o	4.08 o	4.08 o
4′′′′	4.11 m	4.11 m	4.11 m	4.05 m	4.10 m	4.10 m
5′′′′	3.66 t (10.8)	3.65 t (10.8)	3.65 t (10.8)	3.58 t (10.8)	3.66 t (10.8)	3.66 t (10.8)
	4.21 o	4.20 o	4.20 o	4.17 o	4.22 o	4.22 o
	**Xyl**	**Xyl**	**Xyl**	**Glc**	**Glc**	**Glc**
1′′′′′	5.42 d (7.8)	5.42 d (7.8)	5.42 d (7.8)	4.82 d (7.8)	4.82 d (7.8)	4.83 d (7.8)
2′′′′′	3.97 m	3.97 m	3.97 m	4.02 o	4.02 o	4.02 o
3′′′′′	4.07 o	4.07 o	4.07 o	4.23 o	4.23 o	4.23 o
4′′′′′	4.49 m	4.49 m	4.49 m	4.23 o	4.23 o	4.23 o
5′′′′′	3.48 t (10.8)	3.49 t (10.8)	3.48 t (10.8)	3.93 m	3.91 m	3.91 m
	4.77 o	4.75 o	4.77 d (10.8)	4.53 m	4.53 m	4.53 m
	**Glc**	**Glc**	**Glc**			
1′′′′′′	4.81 d (7.8)	4.81 d (7.8)	4.81 d (7.8)			
2′′′′′′	4.01 o	4.01 o	4.02 o			
3′′′′′′	4.23 o	4.23 o	4.23 o			
4′′′′′′	4.22 o	4.23 o	4.23 o			
5′′′′′′	3.93 m	3.93 m	3.92 m			
6′′′′′′	4.53 m	4.53 m	4.53 m			

o: overlapped with other signals; m: multiplet signals.

**Table 3:** ^1^H-NMR data (*J* in Hz) for compounds **7**–**12** in pyridine-*d*_5_ (δ in ppm).

Position	7	8	9	10	11	12
1	0.95 m	1.44 m	0.70 m	0.70 m	0.76 m	0.77 m
	1.67 m	1.66 m	1.34 m	1.34 m	1.52 m	1.55 m
2	1.74 m	2.34 m	1.69 m	1.69 m	1.74 o	1.76 m
	2.13 m	2.39 m	1.95 m	1.95 m	2.01 o	2.01 m
3	4.10 m	-	3.82 m	3.82 m	3.89 m	3.90 m
4	2.42 m	5.85 s	1.63 m	1.63 m	1.64 m	1.65 m
	2.64 m	-	1.91 m	1.91 m	1.90 m	1.90 m
5	-	-	0.83 m	0.83 m	0.87 m	0.87 m
6	5.28 d (4.8)	1.89 m	1.11–1.14 o	1.12–1.15 o	1.14 m	1.11–1.14 o
		2.37 m	1.11–1.14 o	1.12–1.15 o	1.17 m	1.11–1.14 o
7	1.48 m	0.88 m	0.73 m	0.73 m	0.78 m	0.76 m
	1.84 m	1.68 m	1.56 m	1.56 m	1.47 d (10.2)	1.47 br d (9.6)
8	1.48 m	2.14 brd (13.8)	1.74 m	1.74 m	1.36 m	1.36 m
9	0.87 m	1.19 m	1.35 m	1.35 m	0.49 m	0.47 m
10	-	-	-	-	-	-
11	1.40 0	2.26 o	2.24 dd (4.8, 13.8)	2.24 dd (4.8, 13.8)	1.24 o	1.17 m
	1.42 m	2.50 t (13.8)	2.40 t (13.8)	2.40 t (13.8)	1.38 o	1.36 m
12	1.13 m	-	-	-	1.02 m	0.99 m
	1.74 m	-			2.13 br s	1.57 m
13	-	-	-	-	-	-
14	0.84 m	1.21 m	0.89 m	0.89 m	0.74 m	0.99 m
15	1.45 m	1.67 m	1.57 m	1.57 m	1.32 m	1.33 m
	2.14 m	2.18 m	2.05 m	2.05 m	2.41 m	1.97 m
16	4.78 m	4.73 m	4.85 m	4.85 m	5.63 m	4.51 m
17	2.42 d (10.2)	3.41 d (10.2)	2.88 dd (6.6, 8.4)	2.88 dd (6.6, 8.4)	2.45 d (7.8)	1.73 m
18	0.71 s	0.99 s	1.11 S	1.10 S	1.17 s	0.72 S
19	0.90 s	1.08 s	0.84 S	0.84 S	0.83 s	0.83 S
20	-	-	2.19 m	2.19 m	-	1.91 o
21	1.63 s	1.75 s	1.54 d (6.6)	1.52 d (6.6)	2.10 s	1.05 d (6.6)
22	-	-	-	-	-	-
23	2.24 m	2.24 m	2.01 m	1.95 m	1.23 o	1.95 o
		2.26 m	2.04 m	2.05 m	2.36 o	2.66 dd (4.2,12.6)
24	1.47 m	1.44 m	1.65 m	1.66 m	1.54 m	4.02 m
	1.82 m	1.83 m	2.02 m	2.03 m	1.94 m	-
25	1.93 m	1.95 m	1.61 m	1.61 m	1.86 m	1.90 m
26	3.61 dd (6.0, 9.0)	3.62 dd (6.0, 9.6)	3.60 dd (6.0,9.6)	3.48 o	3.49 o	3.56 t (11.4)
	3.93 m	3.95 m	3.95 m	4.06 m	3.88 o	3.63 br d (11.4)
27	1.04 d (7.8)	1.02 d (6.6)	0.95 d (6.6)	1.01 d (6.6)	0.91 d (7.2)	1.13 d (6.0)
	**Gal**	**Glc**	**Gal**	**Gal**	**Gal**	**Gal**
1′	4.88 d (7.8)	4.81 d (7.8)	4.82 d (7.8)	4.82 d (7.8)	4.83 d (7.8)	4.84 d (7.8)
2′	4.41 o	4.01 t (8.4)	4.45 o	4.45 o	4.45 o	4.46 o
3′	4.12 o	4.22 o	4.13 o	4.13 o	4.13 o	4.13 o
4′	4.59 o	4.21 o	4.48 o	4.48 o	4.48 o	4.48 o
5′	4.02 m	3.93 m	4.10 o	4.10 o	4.10 o	4.11 o
6′	4.21 m	4.37 dd (5.4, 12.0)	4.19 m	4.19 m	4.19 m	4.18 m
	4.69 t (10.2)	4.53 m	4.68 br s	4.68 br s	4.68 br d (11.4)	4.69 dd (9.6, 15.6)
	**Glc**		**Rha**	**Rha**	**Rha**	**Rha**
1′′	5.19 d (7.8)		6.18 s	6.18 s	6.18 s	6.18 s
2′′	4.42 dd (7.8, 16.8)		4.75 br s	4.75 br s	4.75 br s	4.75 br s
3′′	4.16 t (9.0)		4.53 o	4.53 o	4.53 o	4.53 o
4′′	3.82 t (10.2)		4.22 o	4.22 o	4.22 o	4.23 o
5′′	3.88 m		4.89 m	4.89 m	4.89 m	4.92 m
6′′	4.03 o		1.70 d (6.0)	1.70 d (6.0)	1.70 d (6.0)	1.70 d (6.0)
	4.52 o					
	**Glc**		**Glc**	**Glc**	**Glc**	**Glc**
1′′′	5.56 d (7.8)		4.97 d (7.8)	4.97 d (7.8)	4.97 d (7.8)	4.97 d (7.8)
2′′′	4.06 o		4.26 t (8.4)	4.26 t (8.4)	4.26 t (8.4)	4.26 o
3′′′	4.06 o		4.06 o	4.06 o	4.06 o	4.06 o
4′′′	4.22 o		3.81 o	3.81 o	3.81 o	3.81 o
5′′′	3.89 m		3.82 m	3.82 m	3.82 m	3.82 m
6′′′	4.36 o		4.01 br d (10.8)	4.01 br d (10.8)	4.01 br d (10.8)	4.01 o
	4.57 o		4.51 o	4.51 o	4.51 o	4.51 o
	**Xyl**		**Xyl**	**Xyl**	**Xyl**	**Xyl**
1′′′′	5.23 d (7.8)		5.23 d (7.8)	5.23 d (7.8)	5.23 d (7.8)	5.23 d (7.8)
2′′′′	3.95 o		3.94 o	3.94 o	3.95 o	3.94 o
3′′′′	4.08 o		4.08 o	4.08 o	4.08 o	4.08 o
4′′′′	4.10 m		4.11 m	4.11 m	4.11 m	4.11 m
5′′′′	3.66 t (10.8)		3.65 t (10.8)	3.65 t (10.8)	3.65 t (10.8)	3.65 t (10.8)
	4.22 o		4.20 o	4.20 o	4.20 o	4.20 o
	**Glc**		**Xyl**	**Xyl**	**Xyl**	**Xyl**
1′′′′′	4.83 d (7.8)		5.42 d (7.8)	5.42 d (7.8)	5.42 d (7.8)	5.42 d (7.8)
2′′′′′	4.02 o		3.96 o	3.96 o	3.96 m	3.96 o
3′′′′′	4.23 o		4.07 o	4.07 o	4.07 o	4.07 o
4′′′′′	4.23 o		4.49 m	4.49 m	4.49 m	4.49 m
5′′′′′	3.91 m		3.47 t (10.8)	3.47 t (10.8)	3.46 o	3.47 t (10.8)
6′′′′′	4.38 o		4.75 d (10.8)	4.75 d (10.8)	4.76 o	4.75 d (10.8)
			**Glc**	**Glc**	**Glc**	**Glc**
1′′′′′′			4.81 d (7.8)	4.81 d (7.8)	4.81 d (7.8)	4.92 d (7.8)
2′′′′′′			4.01 t (8.4)	4.01 t (8.4)	4.01 t (8.4)	4.04 o
3′′′′′′			4.22 o	4.22 o	4.22 o	4.19 o
4′′′′′′			4.21 o	4.21 o	4.21 o	4.24 o
5′′′′′′			3.93 m	3.93 m	3.93 m	3.85 m
6′′′′′′			4.37 dd (5.4, 12.0)	4.37 dd (5.4, 12.0)	4.37 dd (5.4, 12.0)	4.37 br d (7.2)
			4.53 m	4.53 m	4.53 m	4.47 o

o: overlapped with other signals; m: multiplet signals.

## Supplementary Materials

Supplementary materials can be accessed at: http://www.mdpi.com/1420-3049/21/4/429/s1.
