# Supplementary material for: Furostanol and Spirostanol Saponins from Tribulus terrestris
Source: Molecules. 2016 Mar 30;21(4):429. doi: 10.3390/molecules21040429 (PMC6273128; doi:10.3390/molecules21040429)
Supplement: Supplementary file 1 [file molecules-21-00429-s001.pdf]

Supplementary Materials: Furostanol and Spirostanol Saponins from *Tribulus terrestris*

Zhen-Fang Wang, Bing-Bing Wang, Yang Zhao, Fang-Xu Wang, Yan Sun, Rui-Jie Guo, Xin-Bo Song, Hai-Li Xin and Xin-Guang Sun

Figures S1–S7 1D and 2D NMR spectrum in C_5D_5N and HRESIMS spectrum in CH_3CN of compound **1**
Figures S8–S13 1D and 2D NMR spectrum in C_5D_5N and HRESIMS spectrum in CH_3CN of compound **2**
Figures S14–S20 1D and 2D NMR spectrum in C_5D_5N and HRESIMS spectrum in CH_3CN of compound **3**
Figures S21–S26 1D and 2D NMR spectrum in C_5D_5N and HRESIMS spectrum in CH_3CN of compound **4**
Figures S27–S32 1D and 2D NMR spectrum in C_5D_5N and HRESIMS spectrum in CH_3CN of compound **5**
Figures S33–S38 1D and 2D NMR spectrum in C_5D_5N and HRESIMS spectrum in CH_3CN of compound **6**
Figures S39–S44 1D and 2D NMR spectrum in C_5D_5N and HRESIMS spectrum in CH_3CN of compound **7**
Figures S45–S50 1D and 2D NMR spectrum in C_5D_5N and HRESIMS spectrum in CH_3CN of compound **8**
Figures S51–S56 1D and 2D NMR spectrum in C_5D_5N and HRESIMS spectrum in CH_3CN of compound **9**
Figures S57–S62 1D and 2D NMR spectrum in C_5D_5N and HRESIMS spectrum in CH_3CN of compound **10**
Figures S63–S68 1D and 2D NMR spectrum in C_5D_5N and HRESIMS spectrum in CH_3CN of compound **11**
Figures S69–S75 1D and 2D NMR spectrum in C_5D_5N and HRESIMS spectrum in CH_3CN of compound **12**

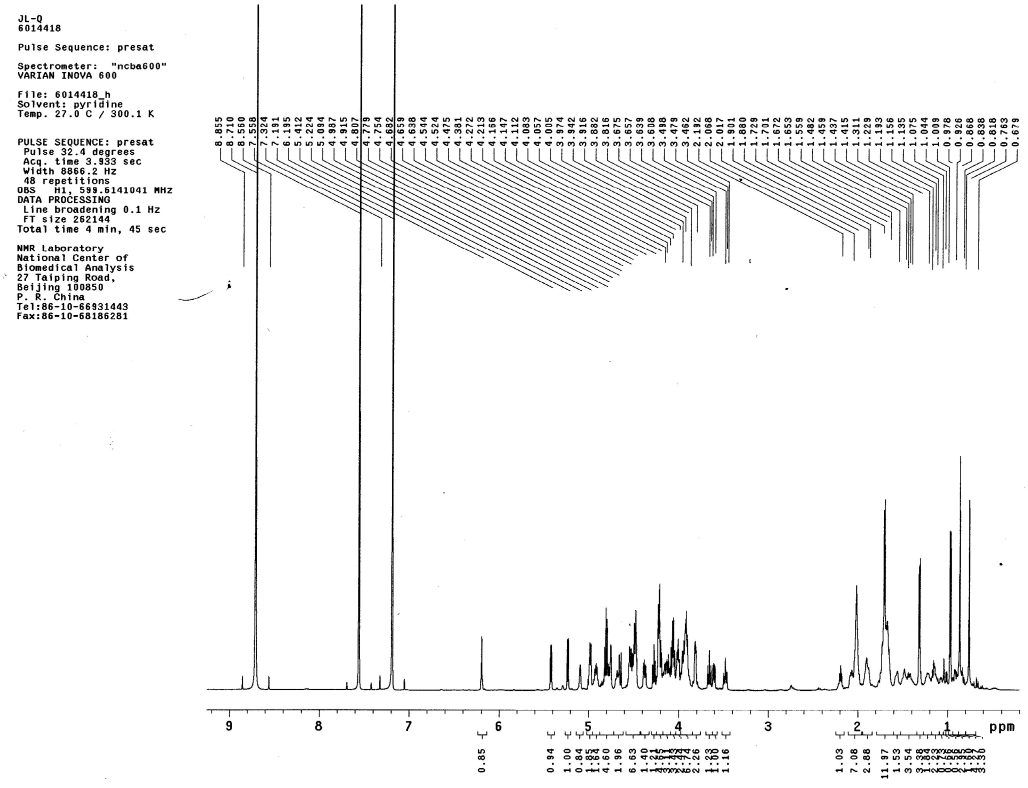

Figure S1. ^{1}H-NMR spectrum of compound **1** in C_5D_5N.

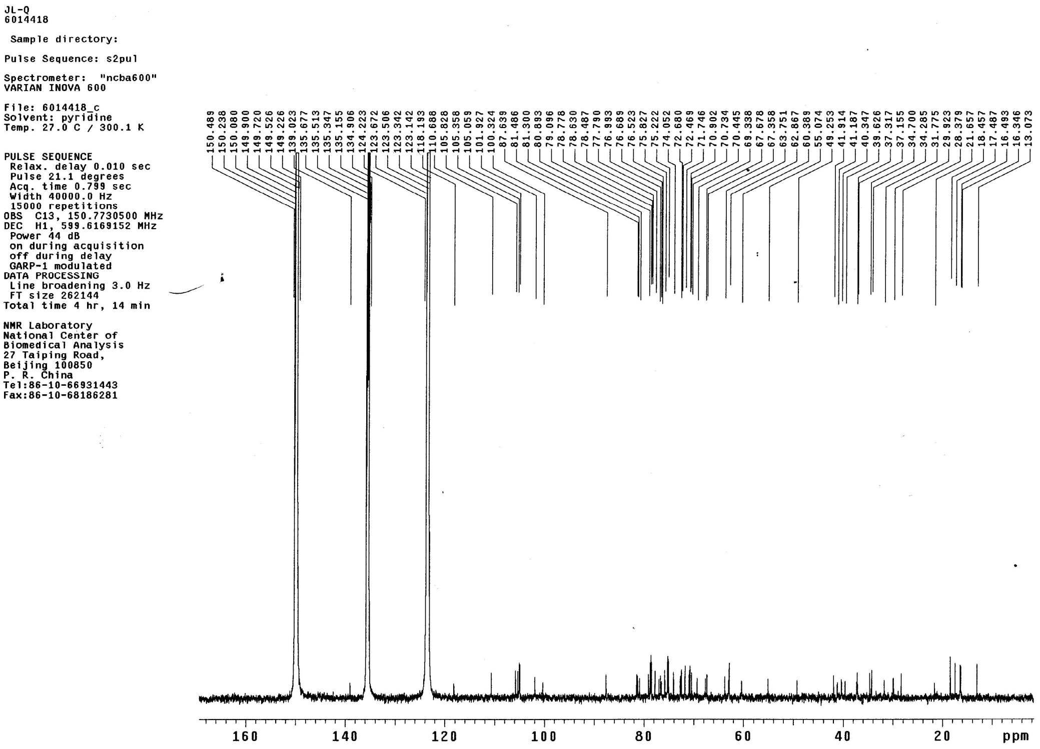

Figure S2. ^{13}C-NMR spectrum of compound **1** in C_5D_5N.

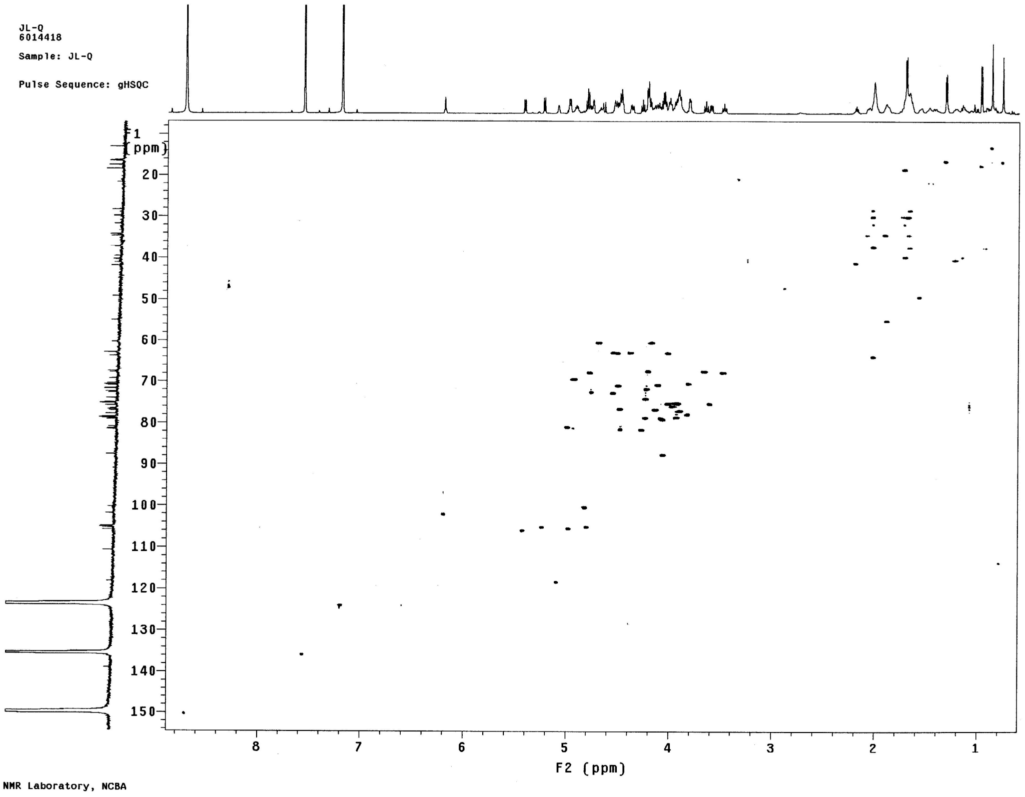

Figure S3. HSQC spectrum of compound **1** in C_5D_5N.

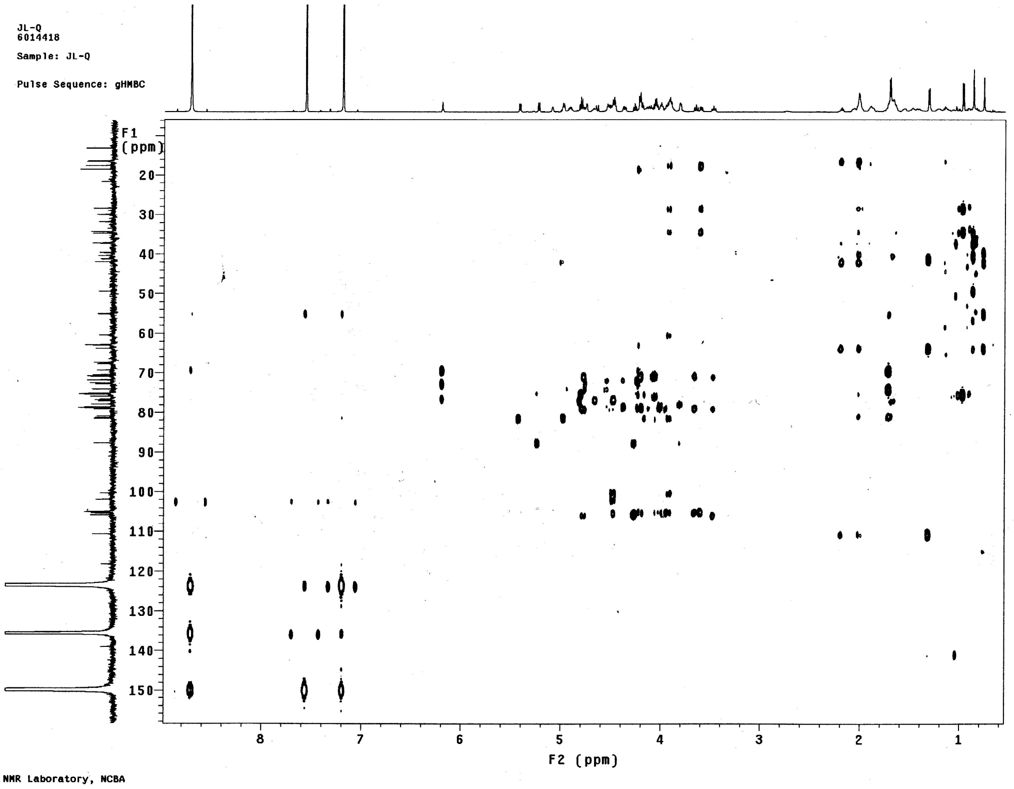

Figure S4. HMBC spectrum of compound **1** in C_5D_5N.

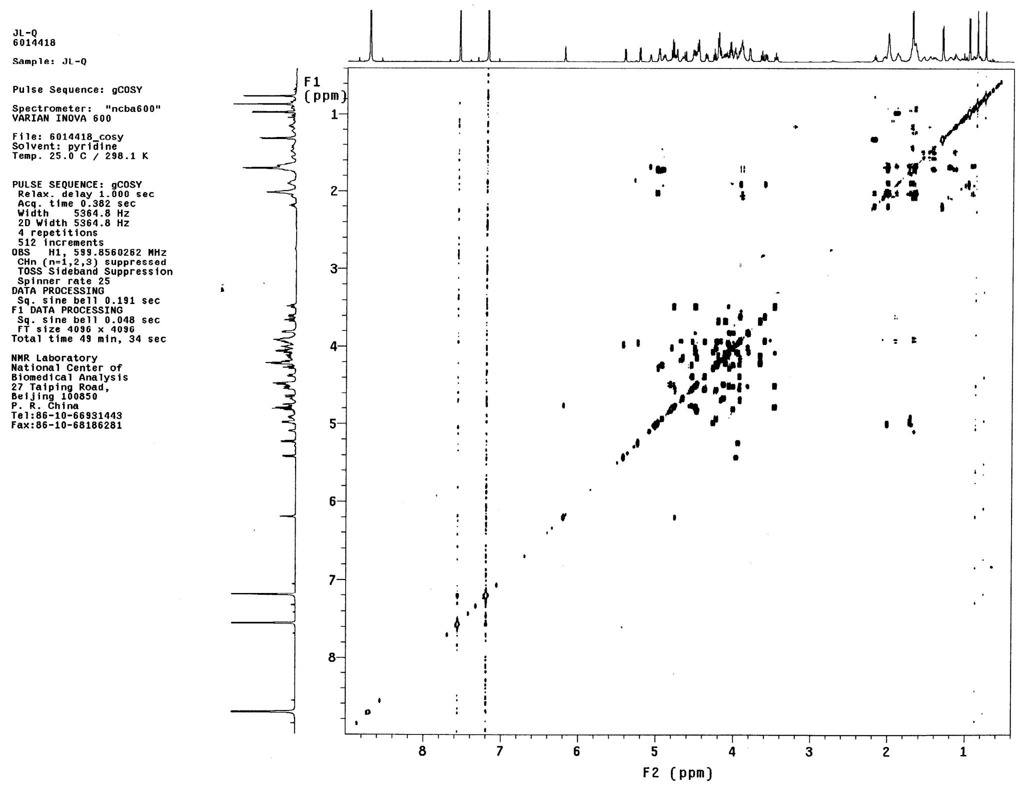

Figure S5. ^1H-^1H COSY spectrum of compound **1** in C_5D_5N.

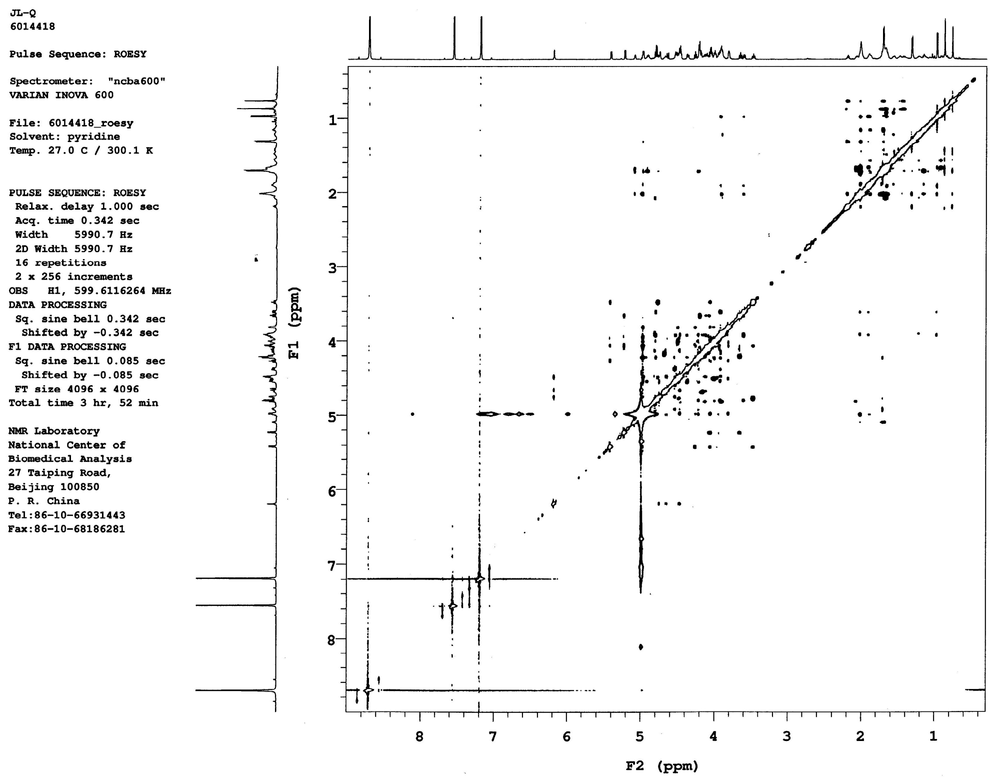

Figure S6. ROESY spectrum of compound **1** in C_5D_5N.

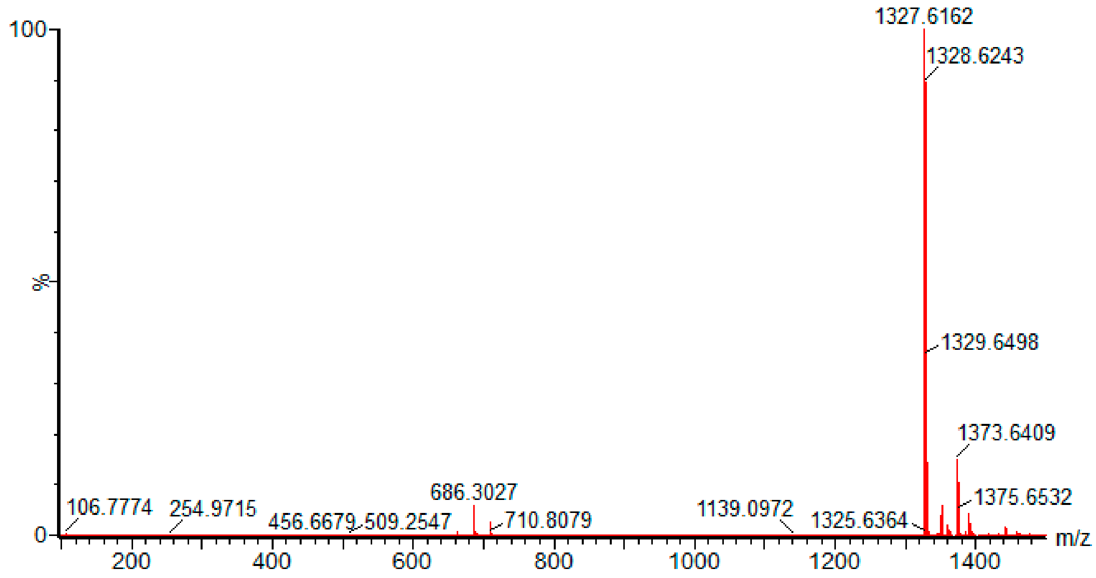

Figure S7. HRESIMS of compound **1** in CH_3CN.

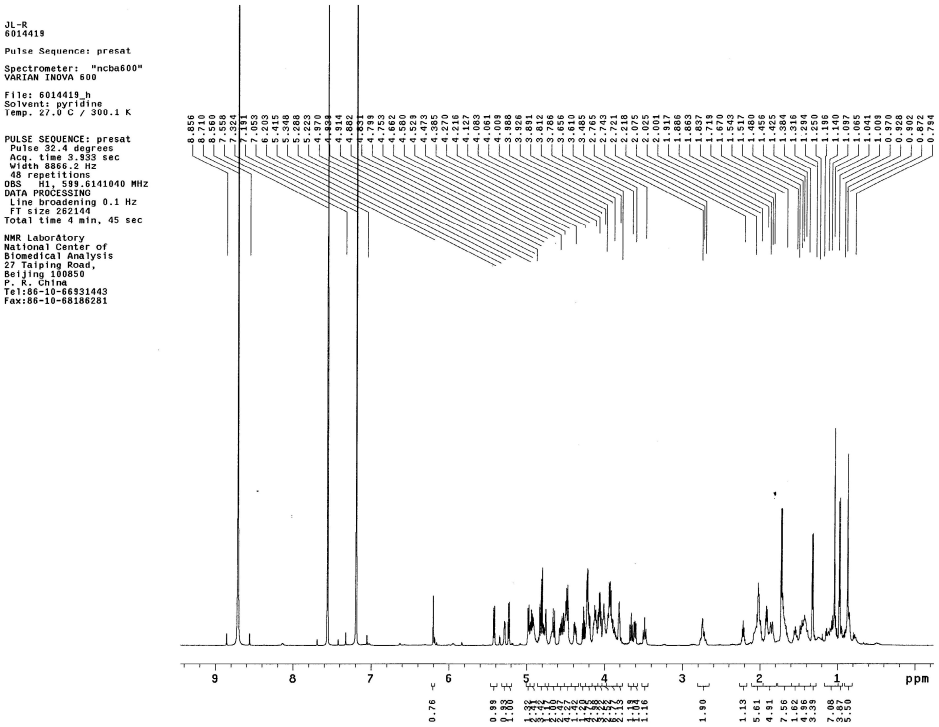

Figure S8. ^{1}H-NMR spectrum of compound **2** in C_5D_5N.

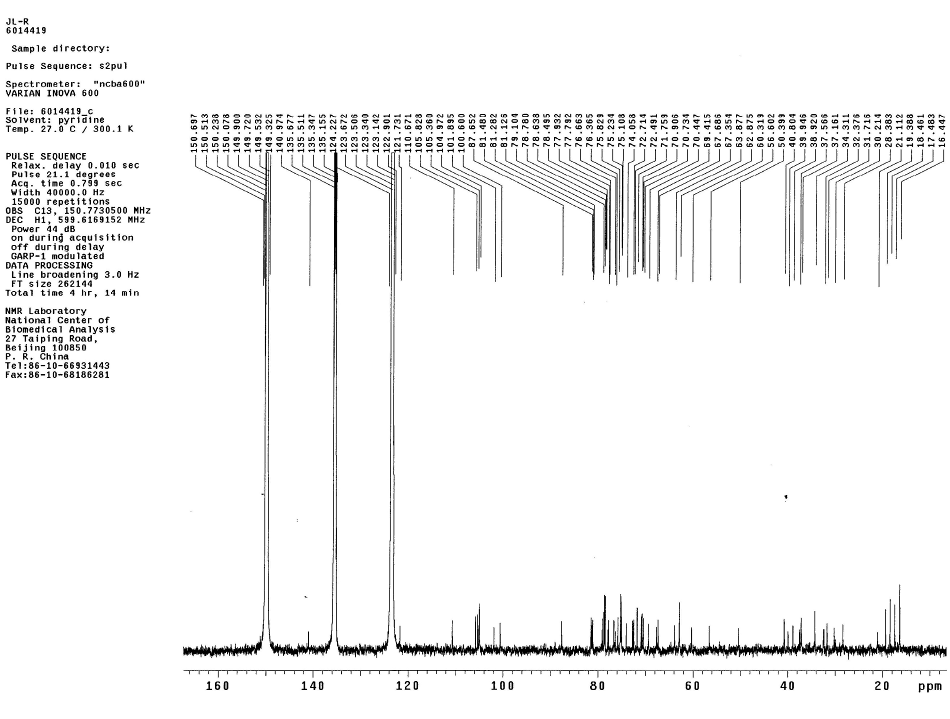

Figure S9. ^{13}C-NMR spectrum of compound **2** in C_5D_5N.

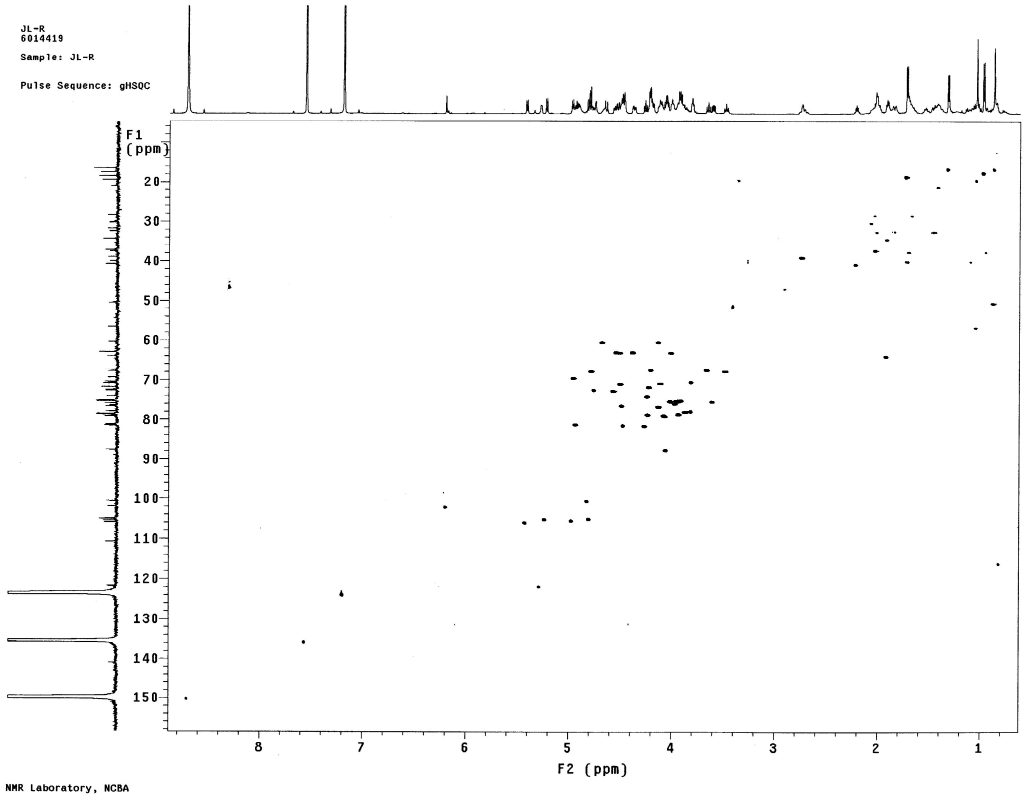

Figure S10. HSQC spectrum of compound **2** in C_5D_5N.

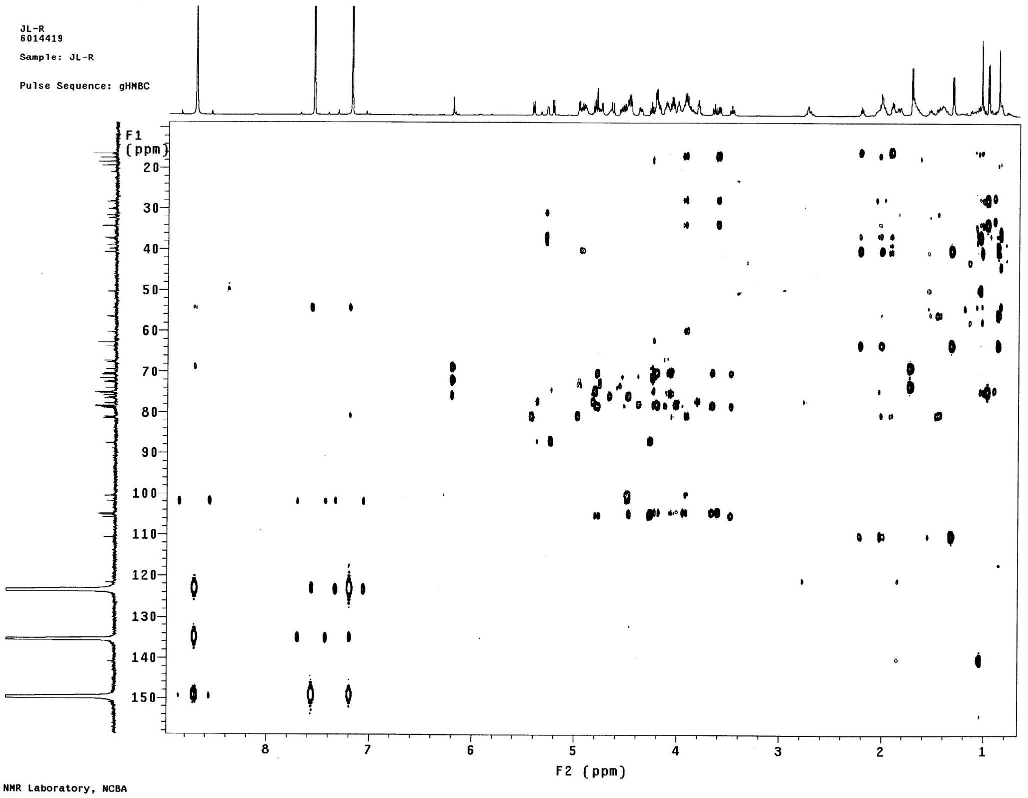

Figure S11. HMBC spectrum of compound **2** in C_5D_5N.

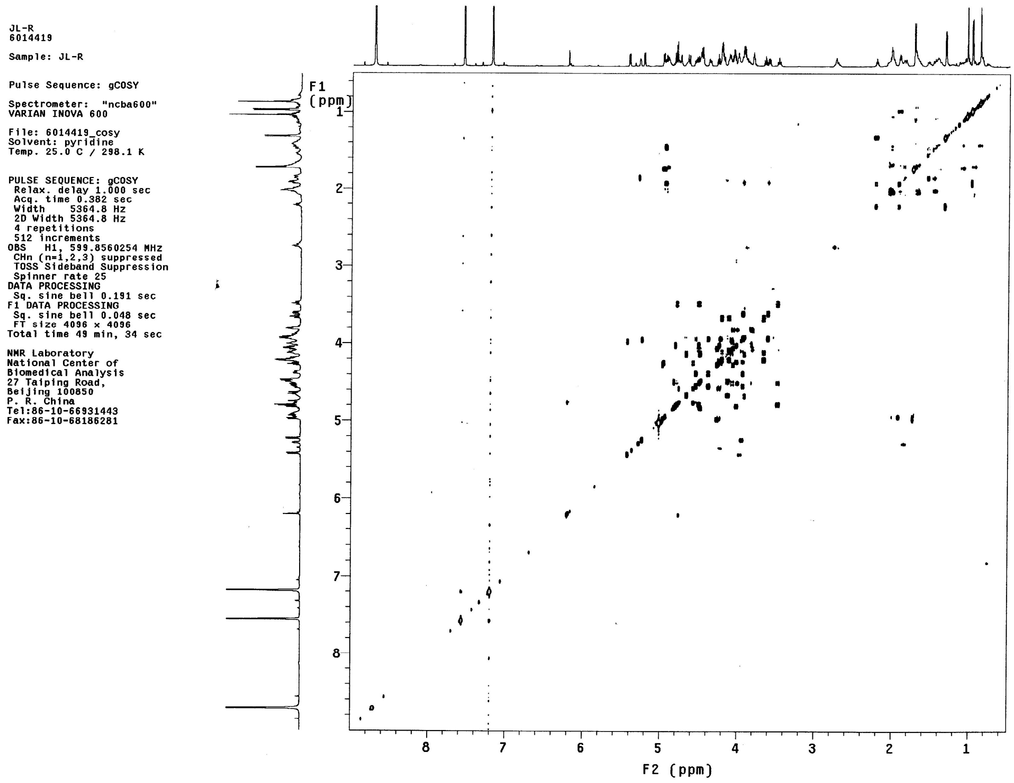

Figure S12. ^1H-^1H COSY spectrum of compound **2** in C_5D_5N.

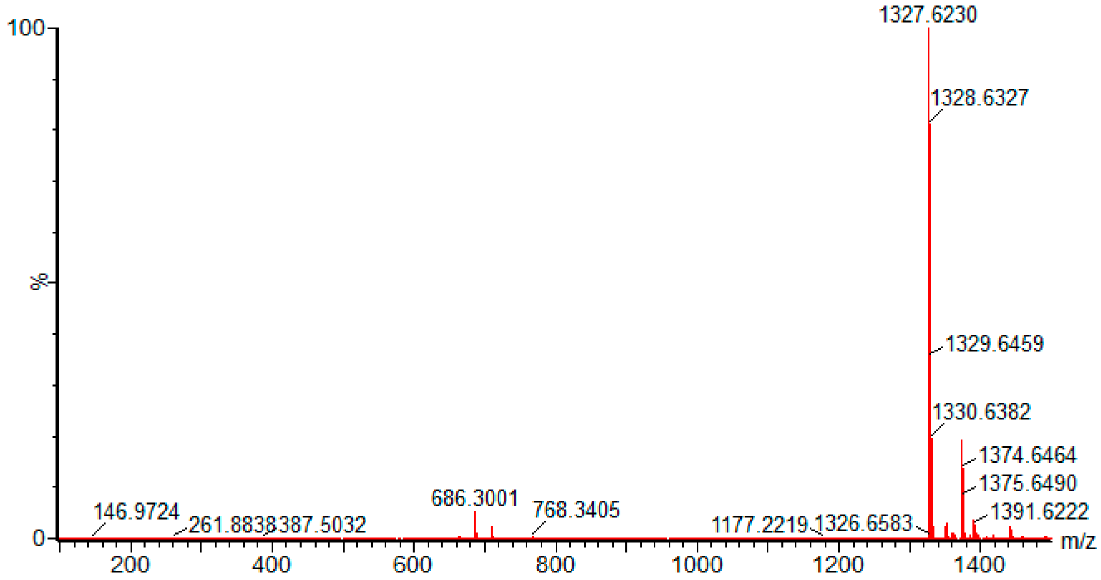

Figure S13. HRESIMS of compound **2** in CH_3CN.

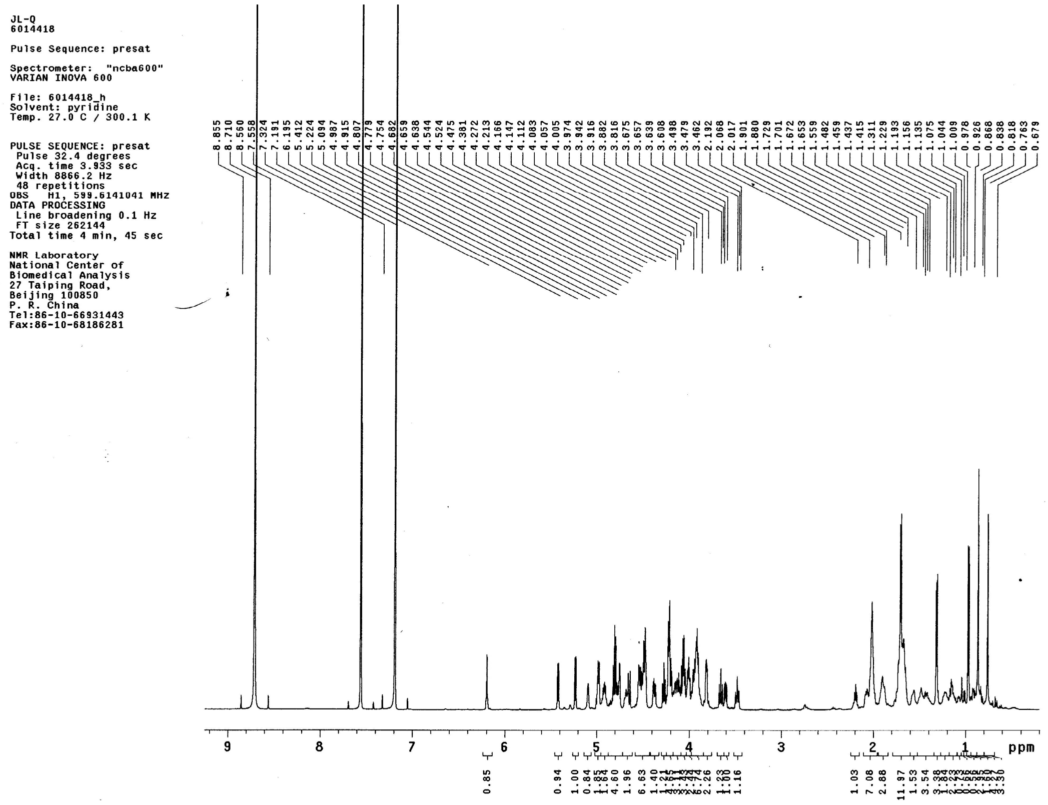

Figure S14. ^{1}H-NMR spectrum of compound **3** in C_5D_5N.

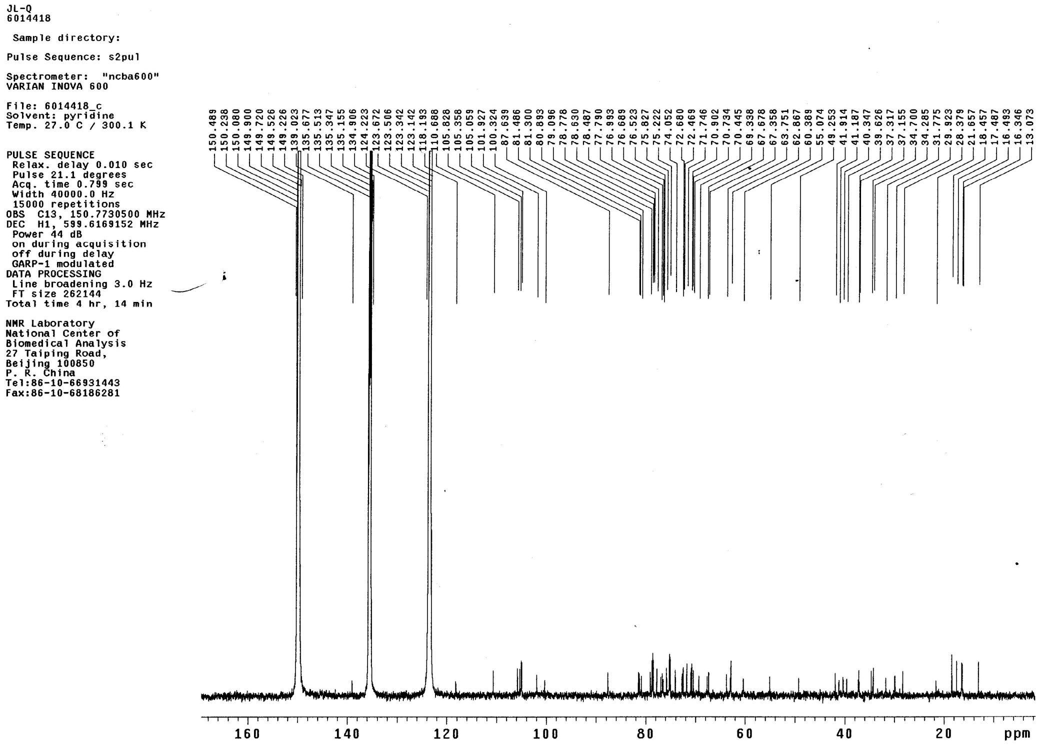

Figure S15. ^{13}C-NMR spectrum of compound **3** in C_5D_5N.

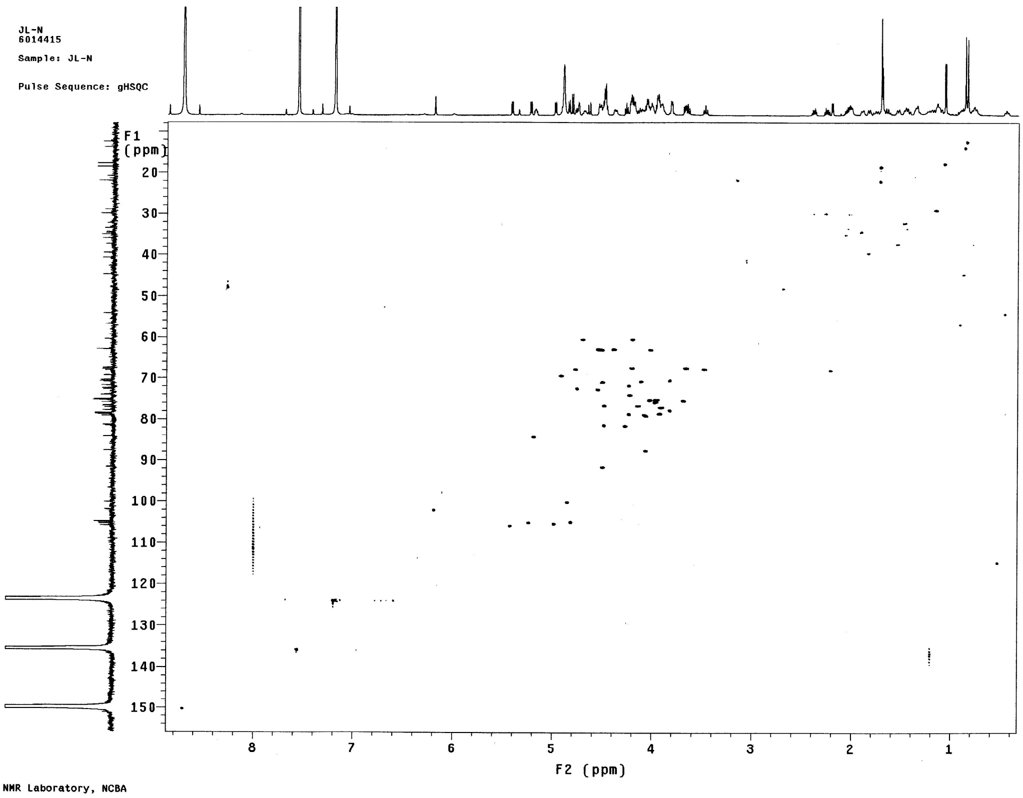

Figure S16. HSQC spectrum of compound **3** in C_5D_5N.

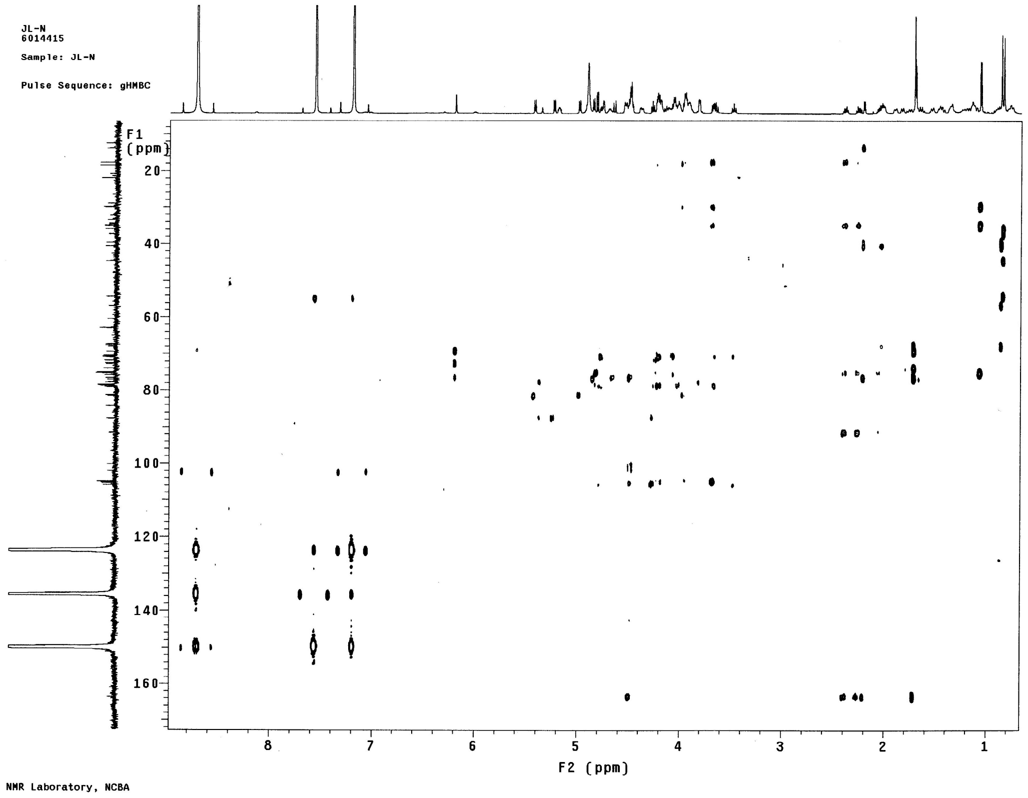

Figure S17. HMBC spectrum of compound **3** in C_5D_5N.

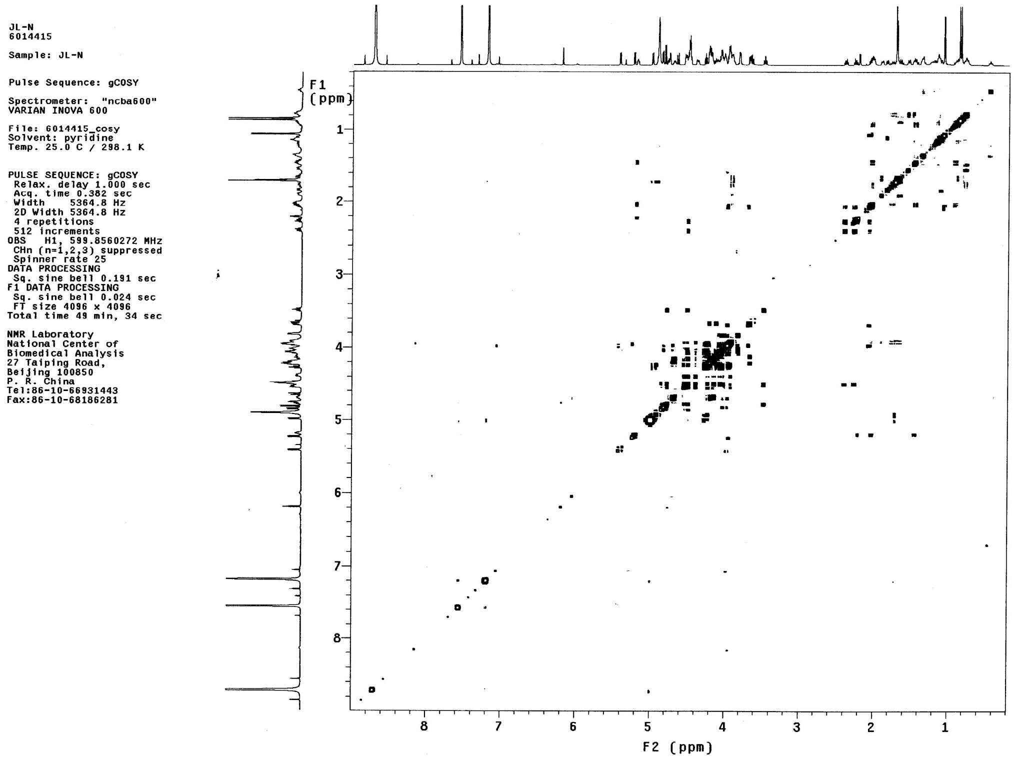

Figure S18. ^1H-^1H COSY spectrum of compound **3** in C_5D_5N.

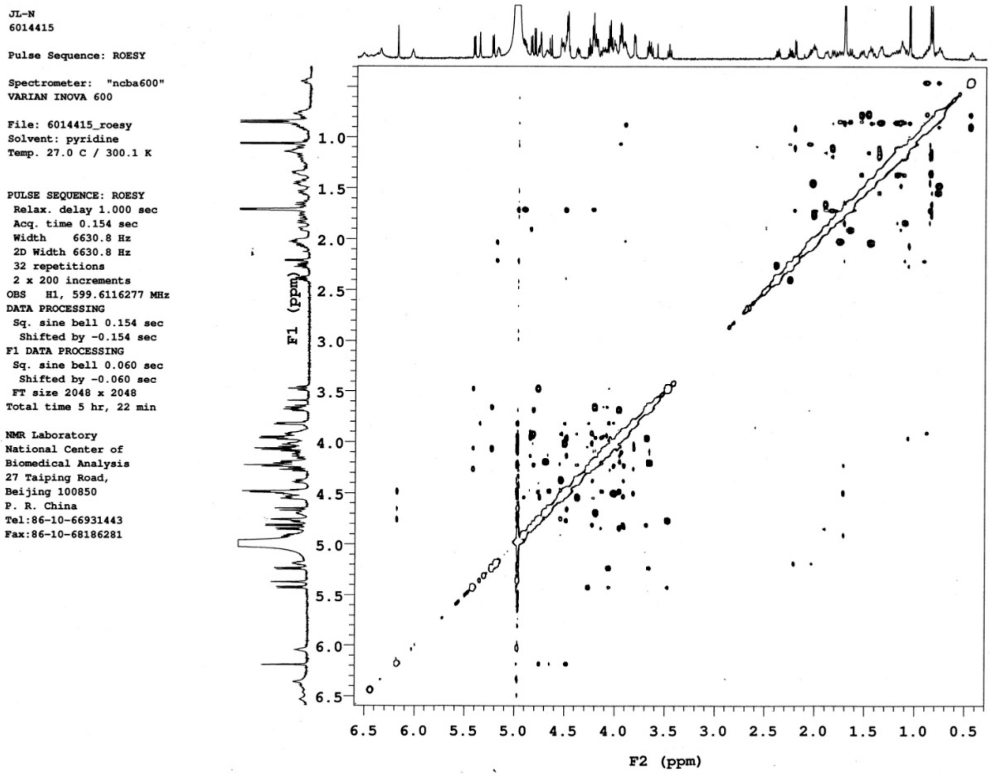

Figure S19. ROESY spectrum of compound **3** in C_5D_5N.

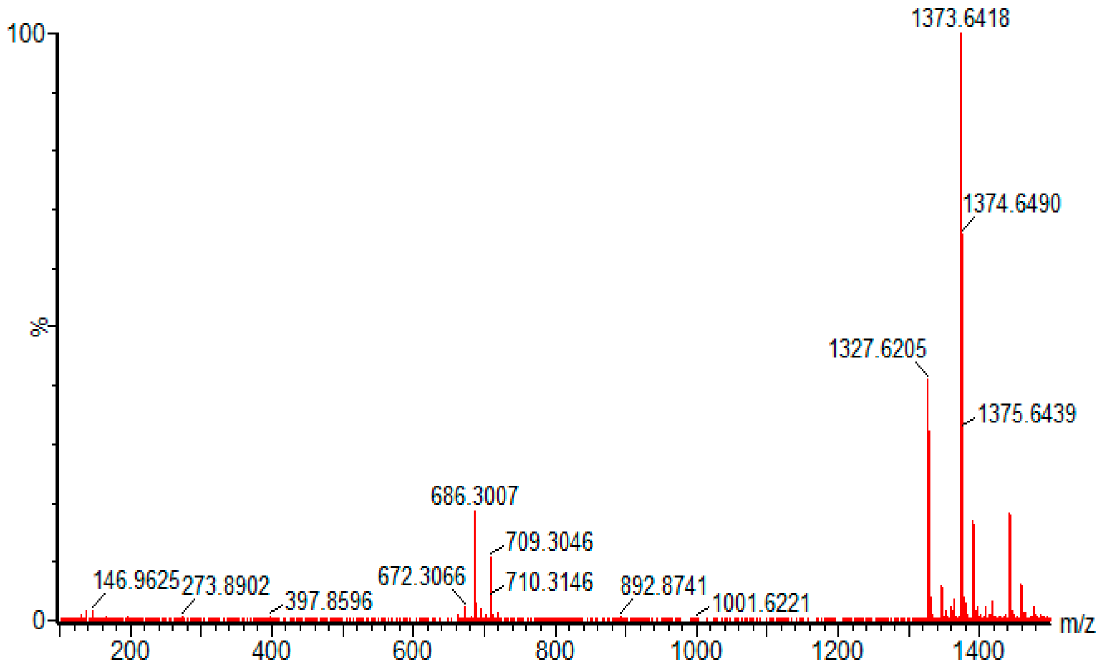

Figure S20. HRESIMS of compound **3** in CH_3CN.

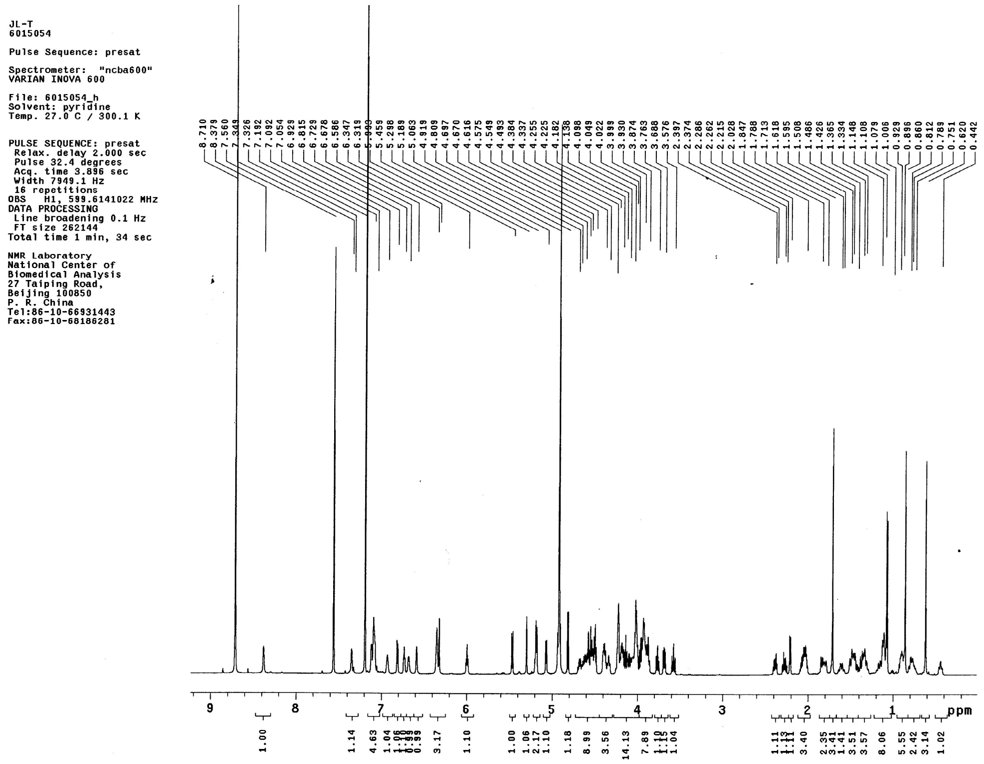

Figure S21. ^{1}H-NMR spectrum of compound **4** in C_5D_5N.

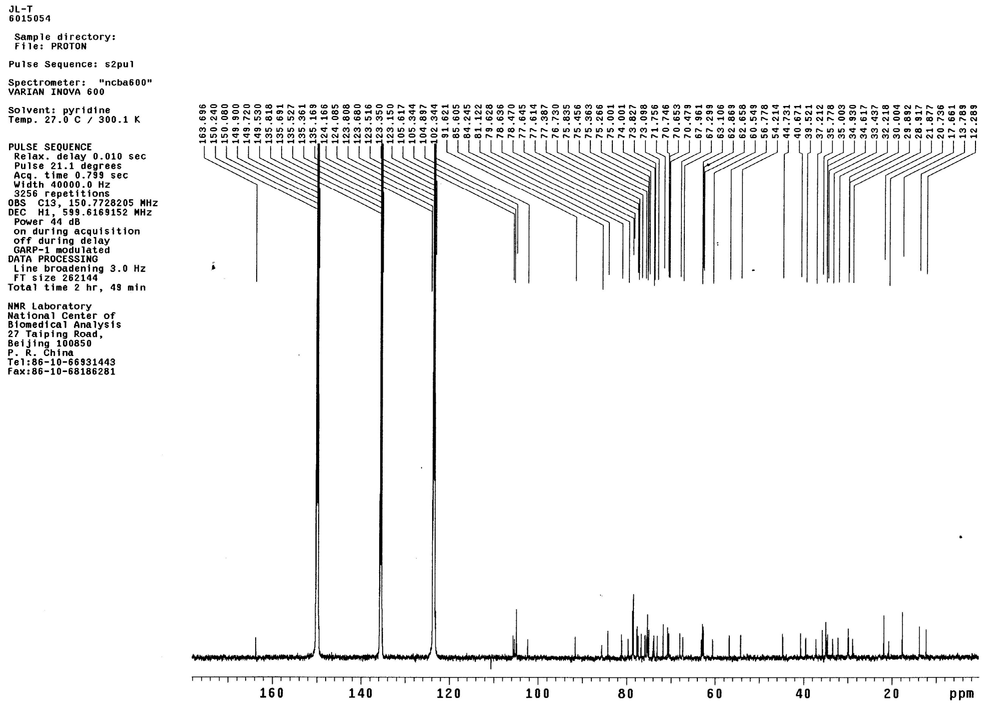

Figure S22. ^{13}C-NMR spectrum of compound **4** in C_5D_5N.

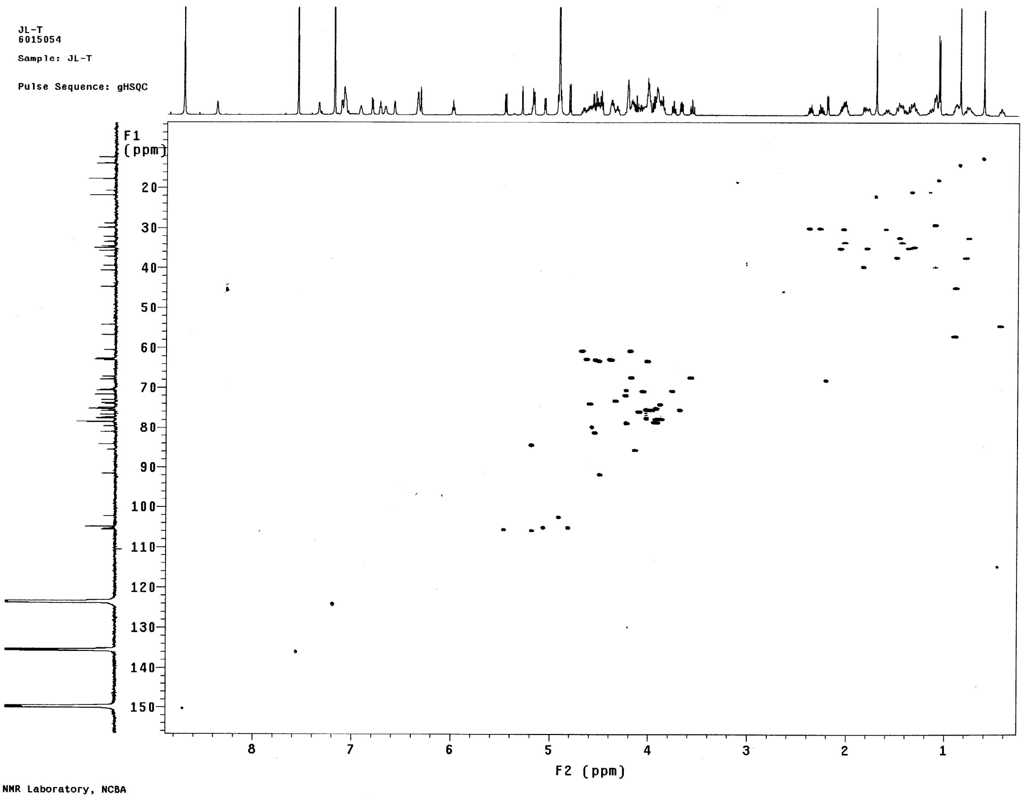

Figure S23. HSQC spectrum of compound **4** in C_5D_5N.

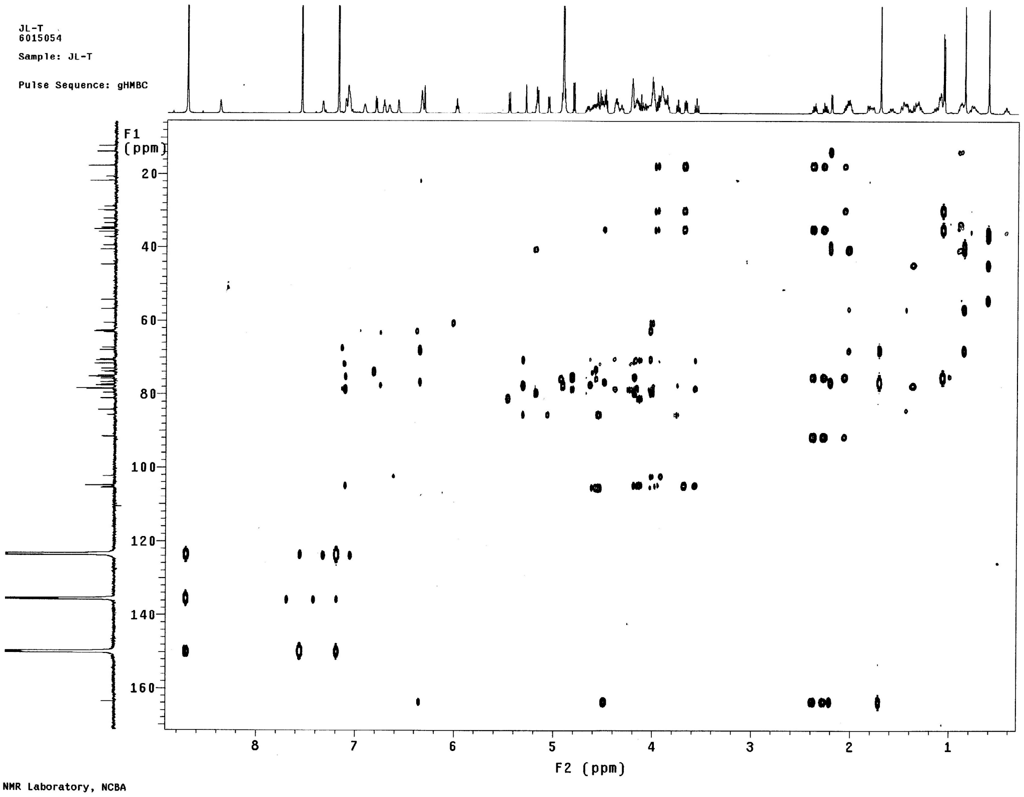

Figure S24. HMBC spectrum of compound **4** in C_5D_5N.

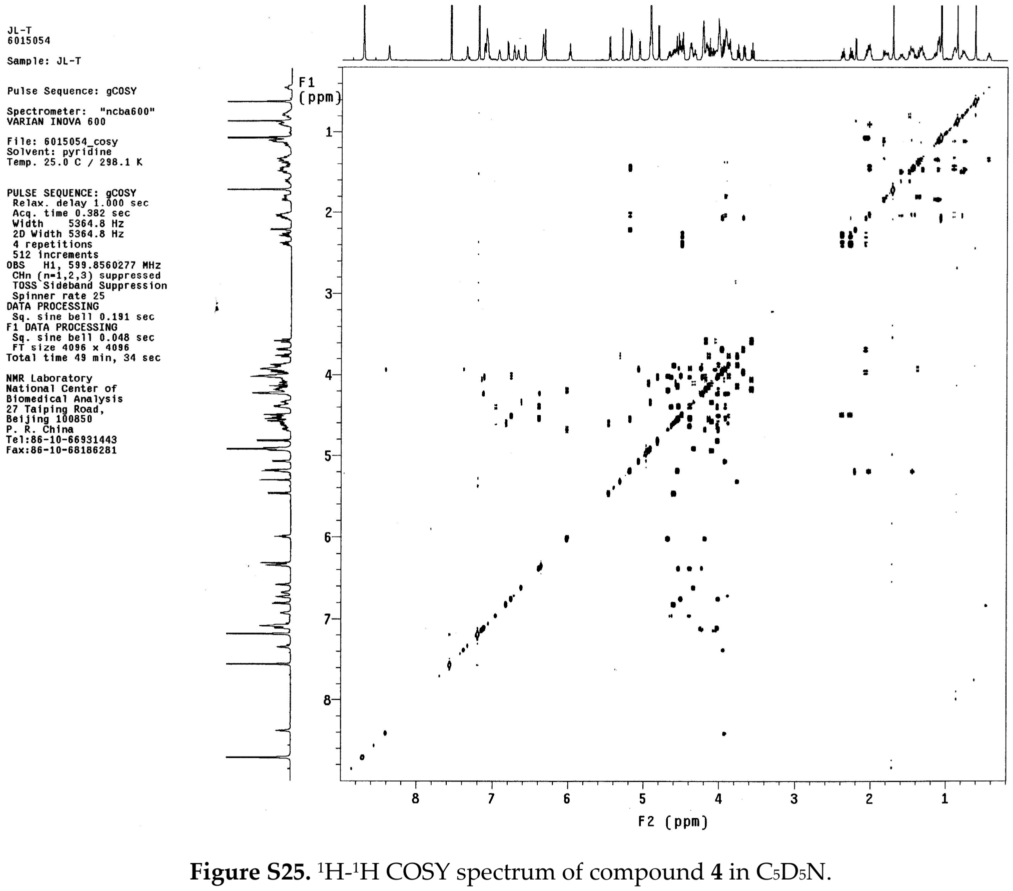

Figure S25. ^1H-^1H COSY spectrum of compound **4** in C_5D_5N.

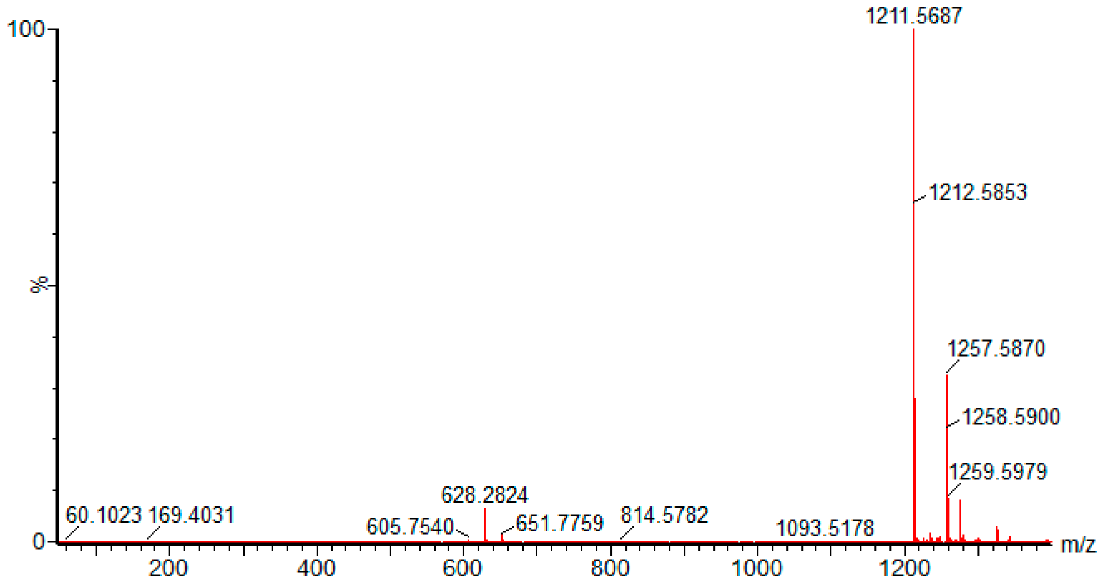

Figure S26. HRESIMS of compound **4** in CH_3CN.

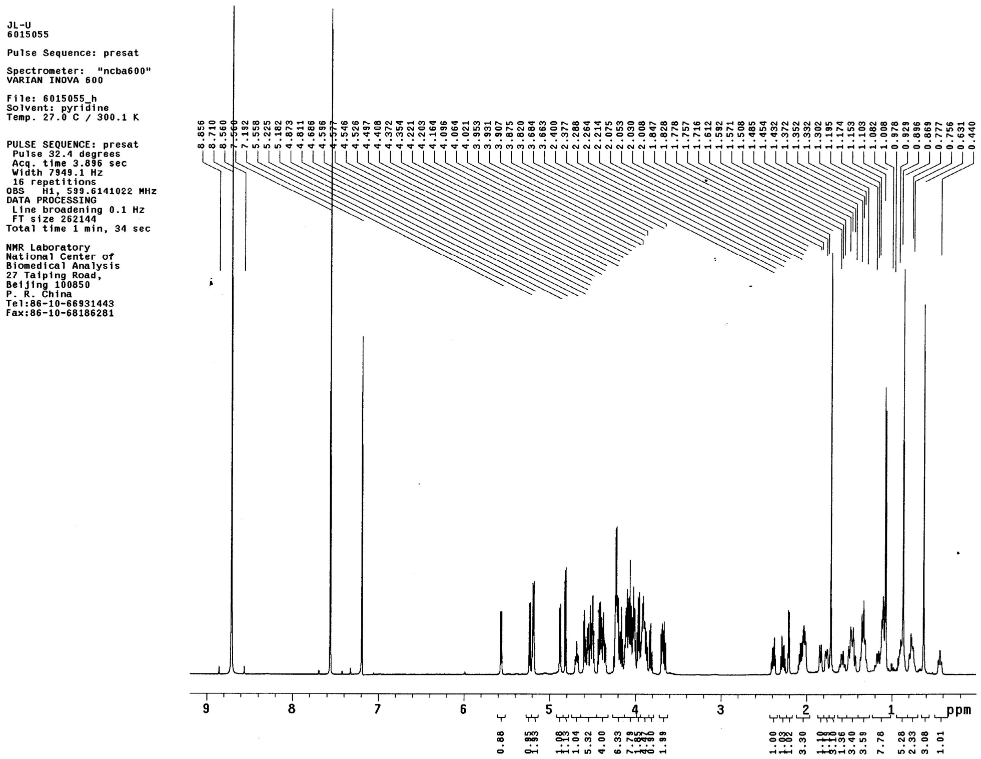

Figure S27. ^{1}H-NMR spectrum of compound **5** in C_5D_5N.

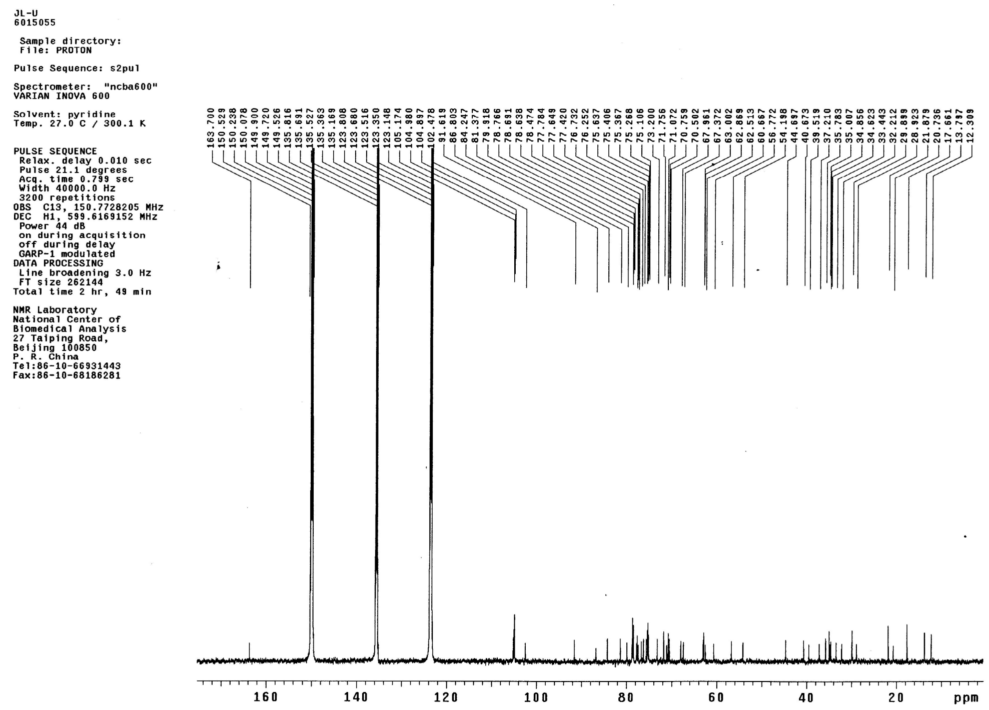

Figure S28. ^{13}C-NMR spectrum of compound **5** in C_5D_5N.

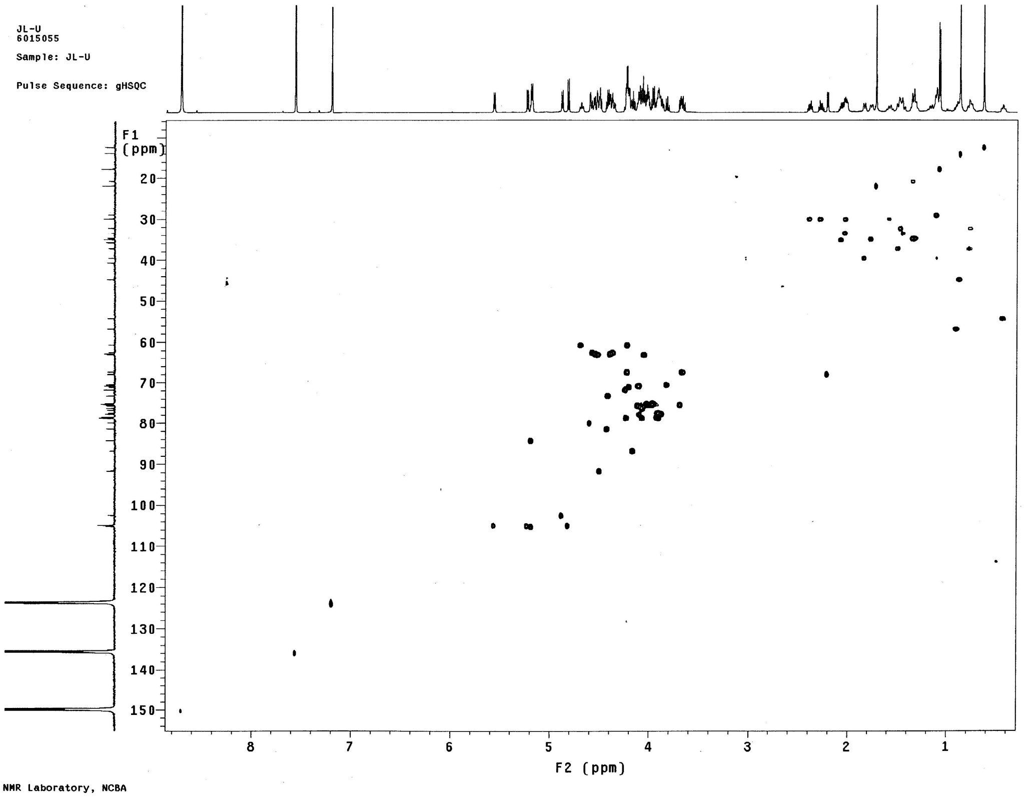

Figure S29. HSQC spectrum of compound **5** in C_5D_5N.

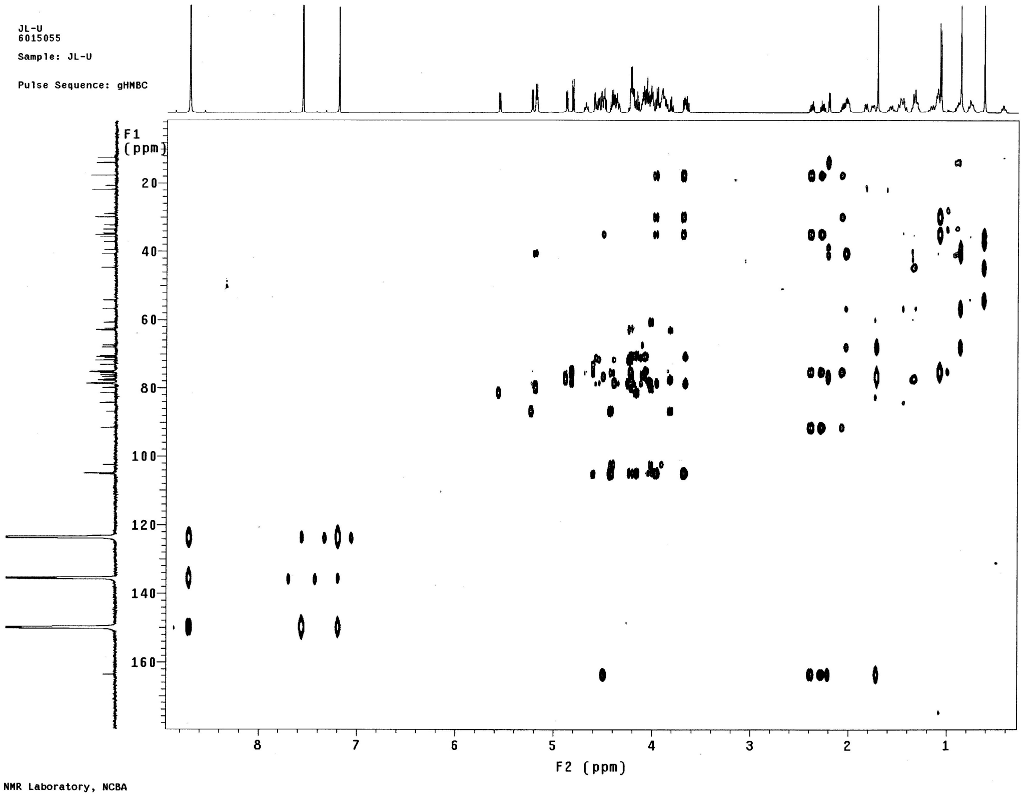

Figure S30. HMBC spectrum of compound **5** in C_5D_5N.

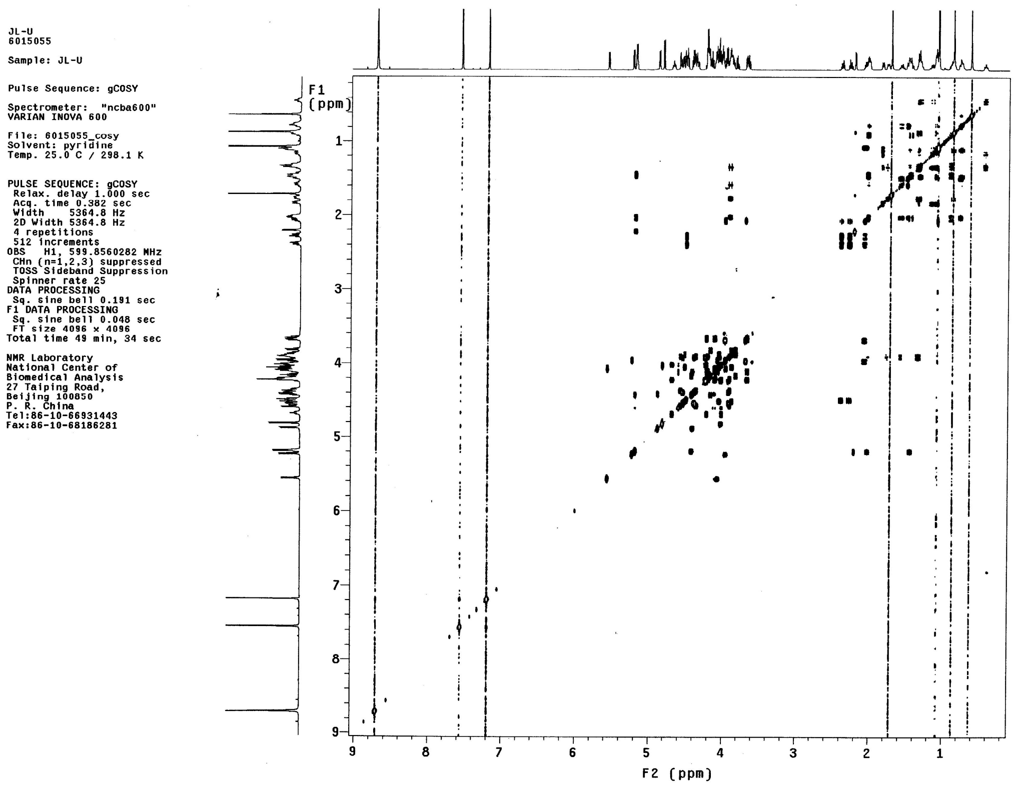

Figure S31. ^{1}H-^{1}H COSY spectrum of compound **5** in C_5D_5N.

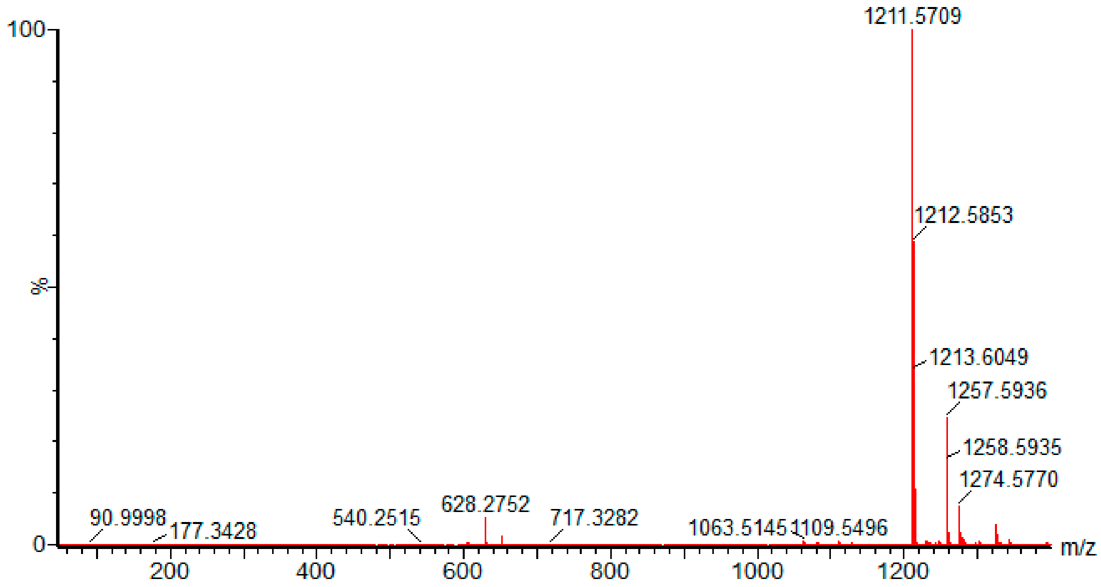

Figure S32. HRESIMS of compound **5** in CH_3CN.

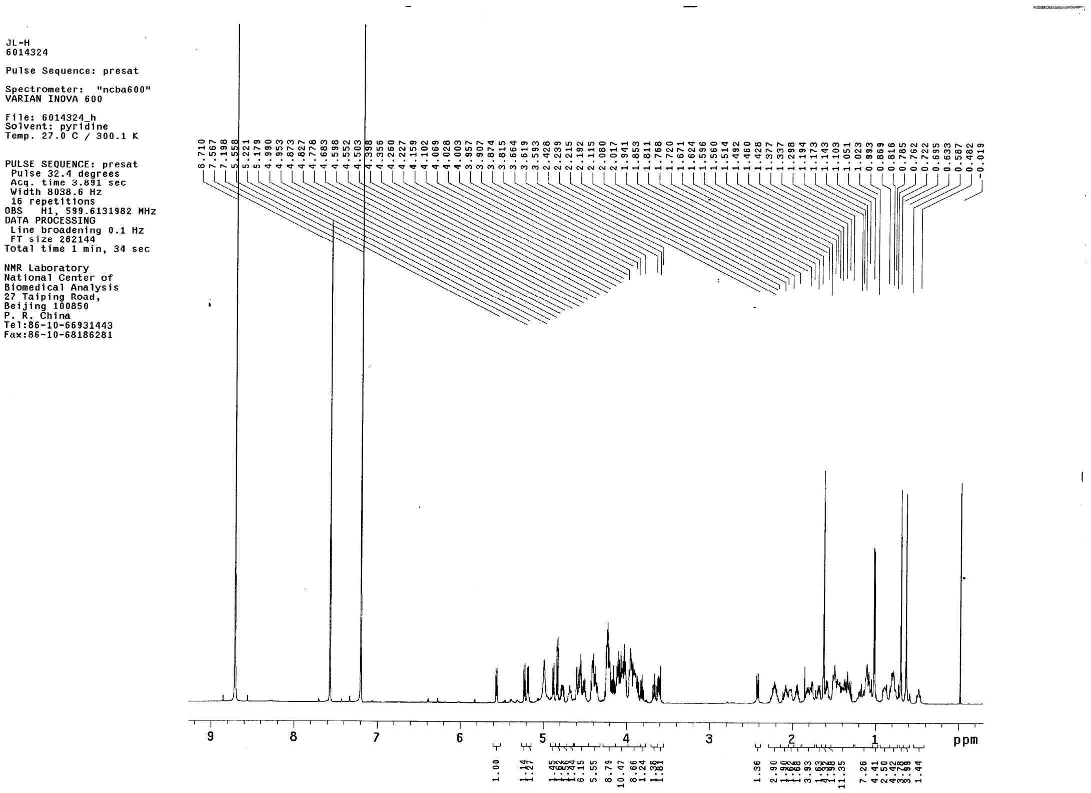

Figure S33. ^{1}H-NMR spectrum of compound **6** in C_5D_5N.

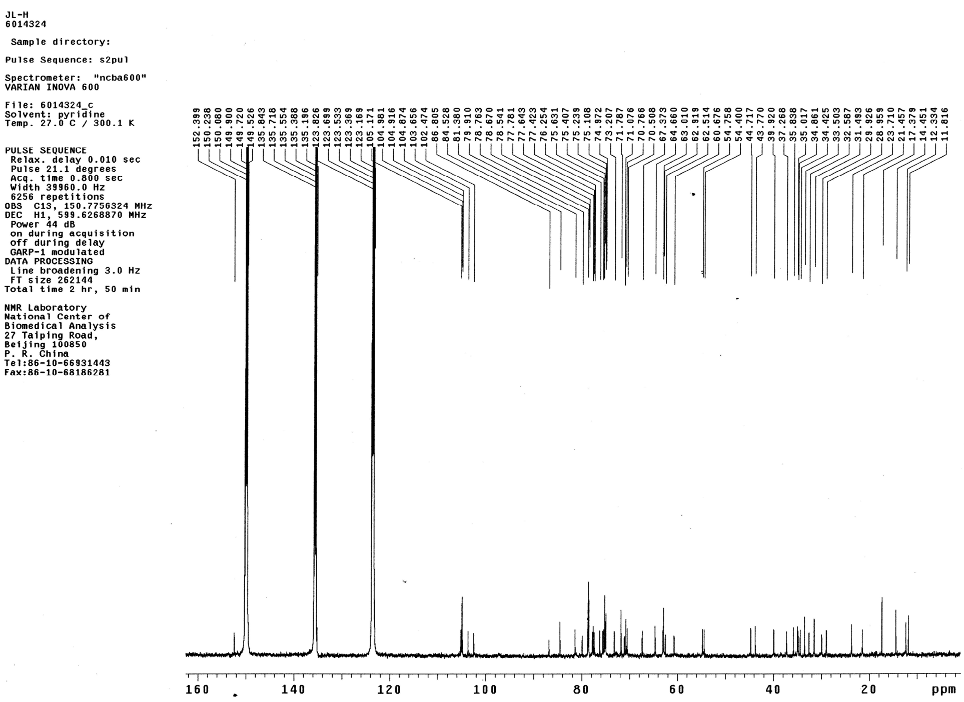

Figure S34. ^{13}C-NMR spectrum of compound **6** in C_5D_5N.

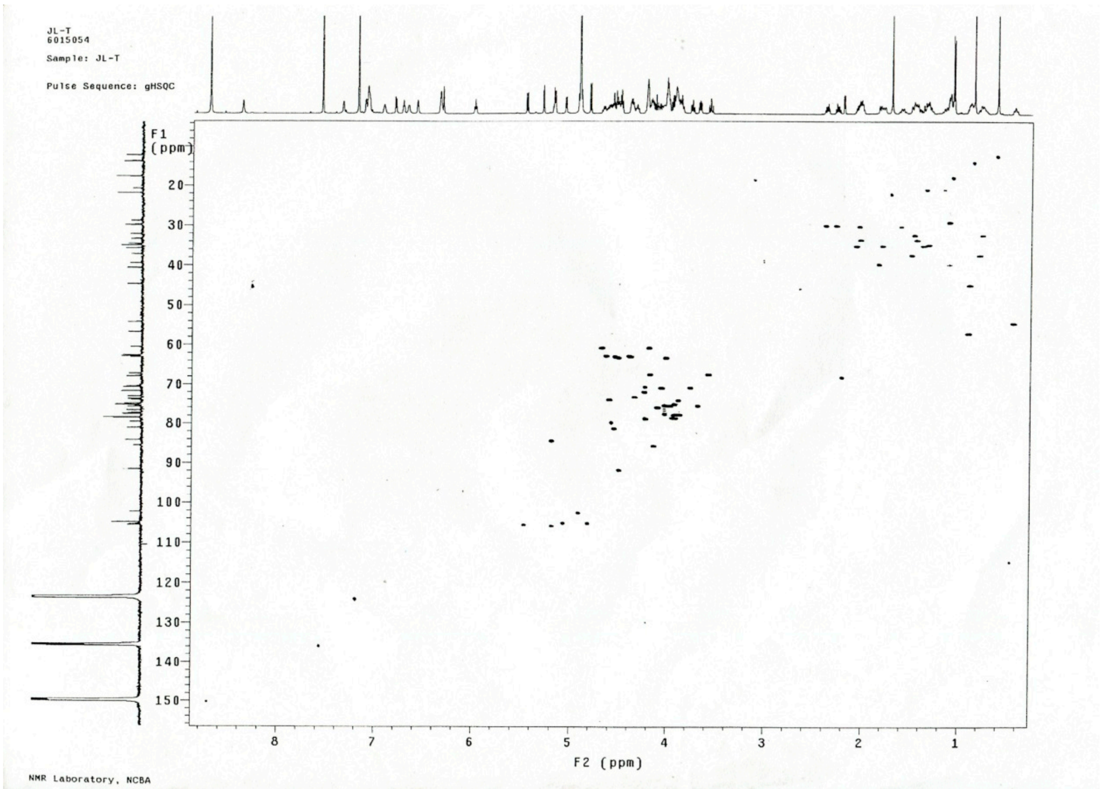

Figure S35. HSQC spectrum of compound **6** in C_5D_5N.

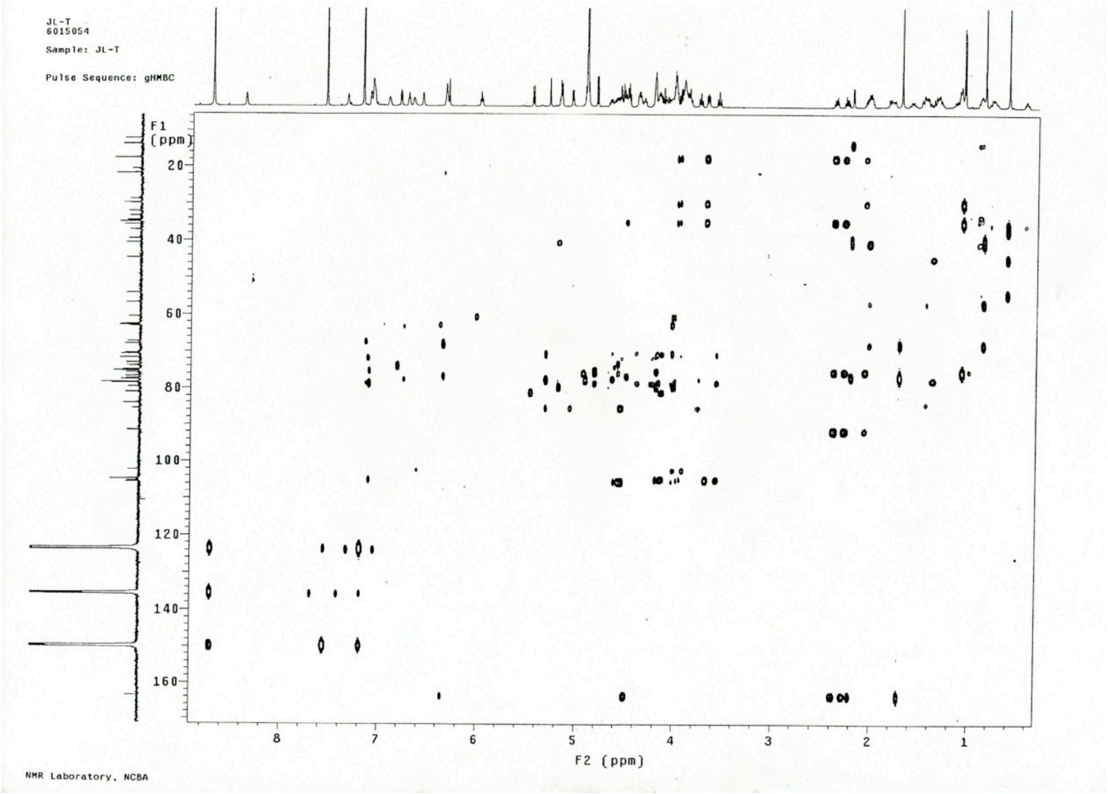

Figure S36. HMBC spectrum of compound **6** in C_5D_5N.

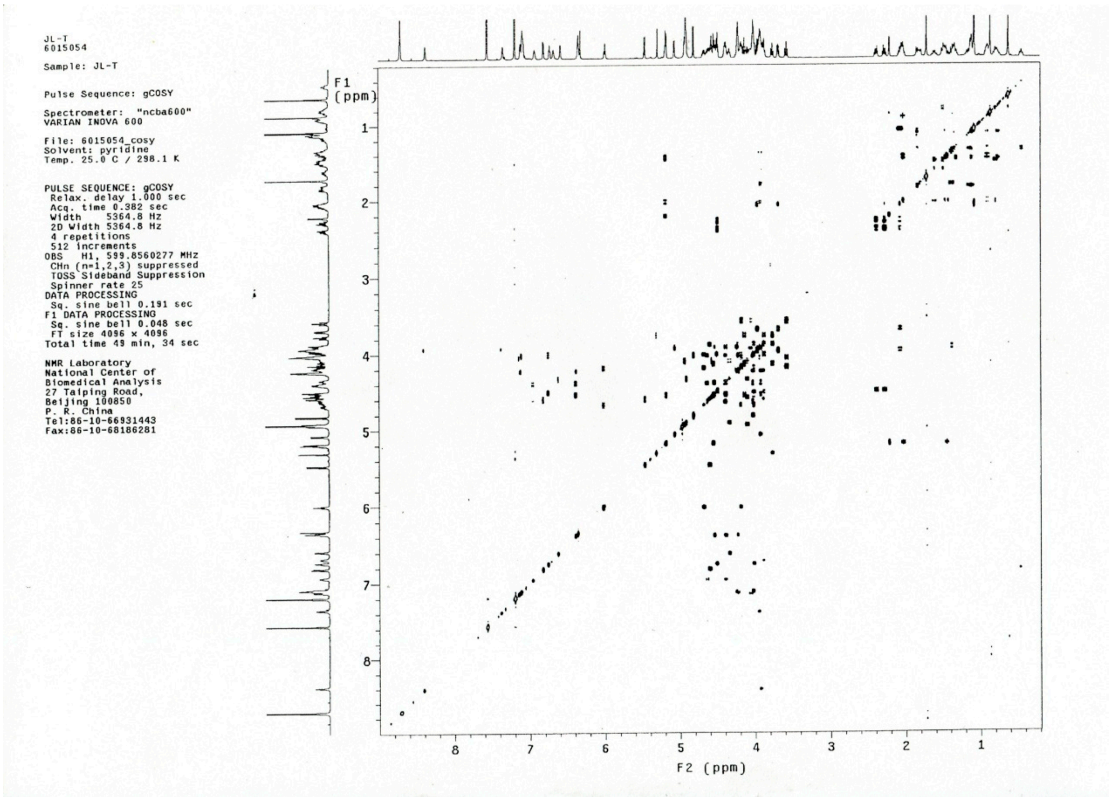

Figure S37. ^{1}H-^{1}H COSY spectrum of compound **6** in C_5D_5N.

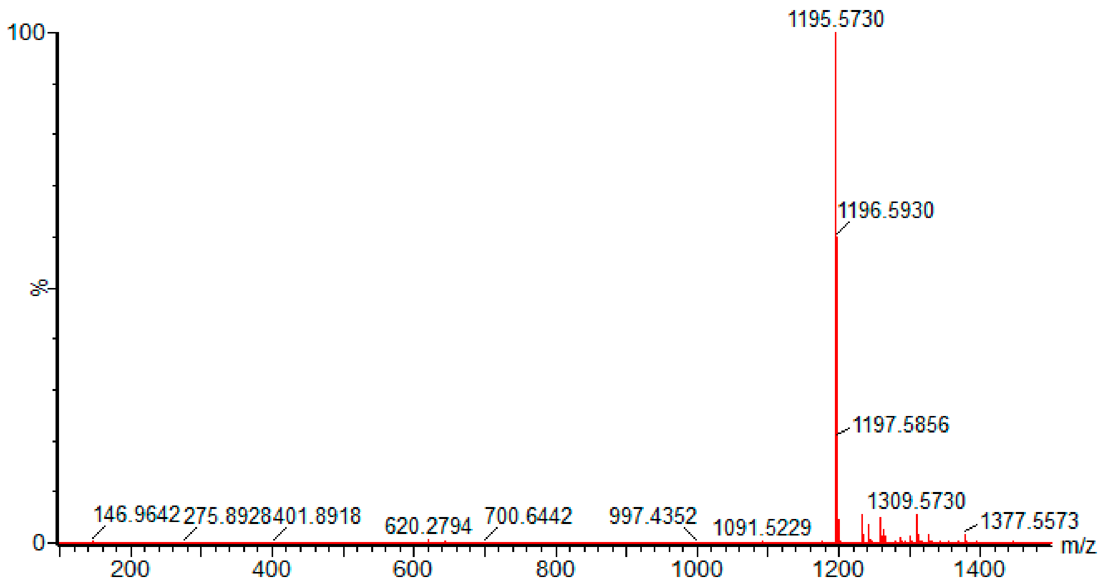

Figure S38. HRESIMS of compound **6** in CH_3CN.

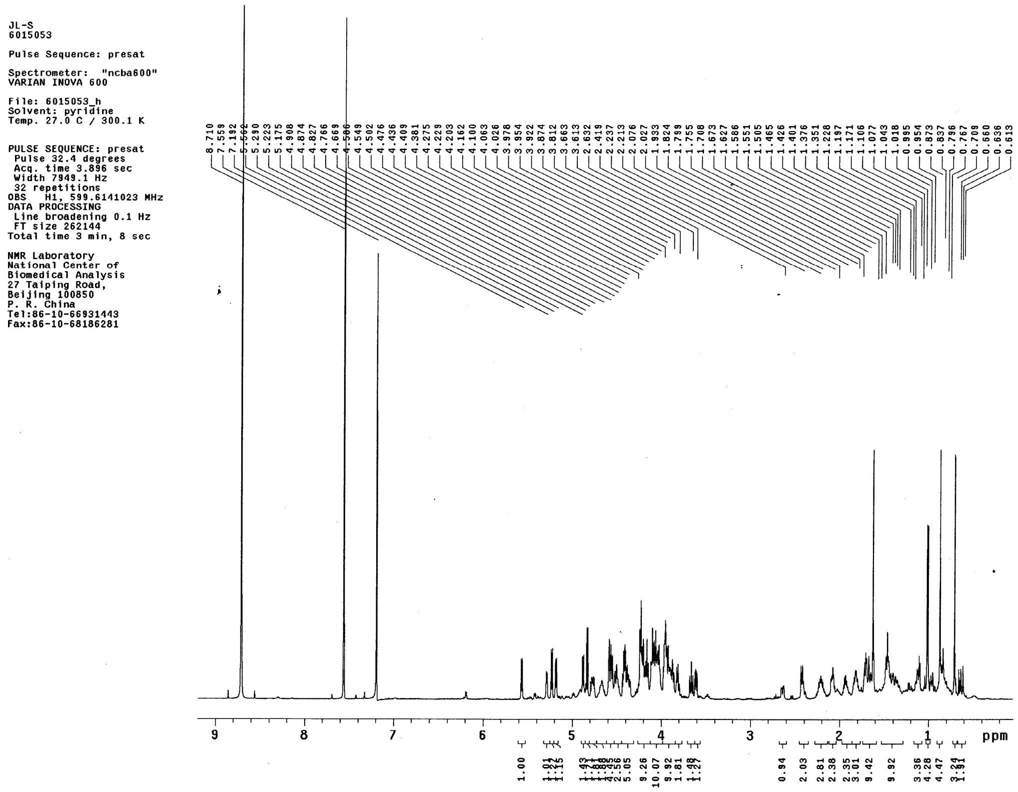

Figure S39. ^{1}H-NMR spectrum of compound **7** in C_5D_5N.

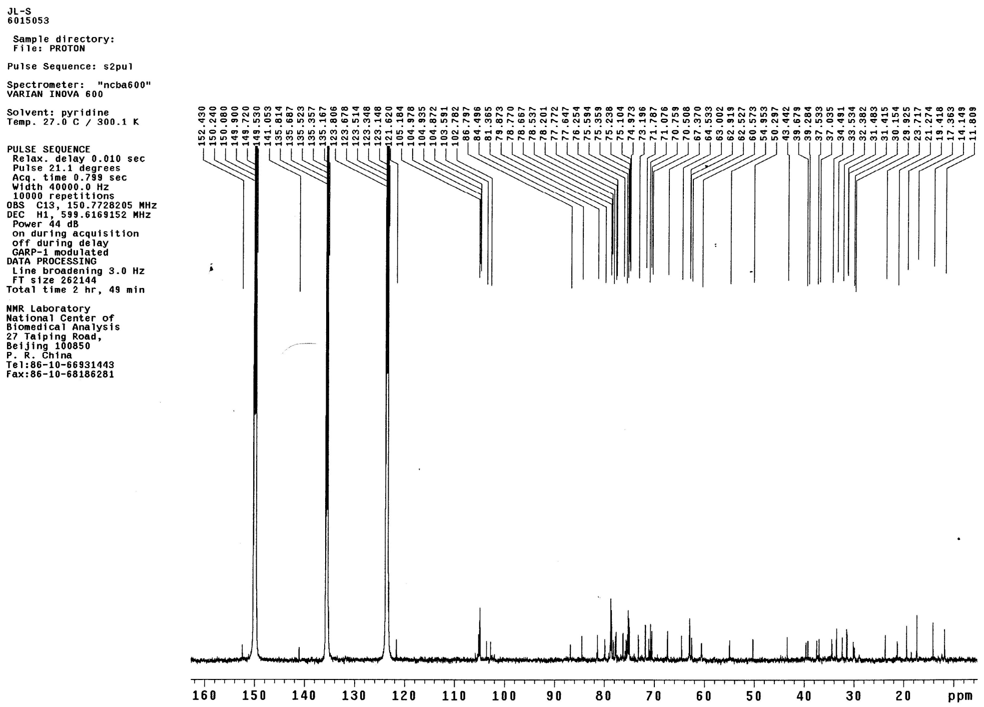

Figure S40. ^{13}C-NMR spectrum of compound **7** in C_5D_5N.

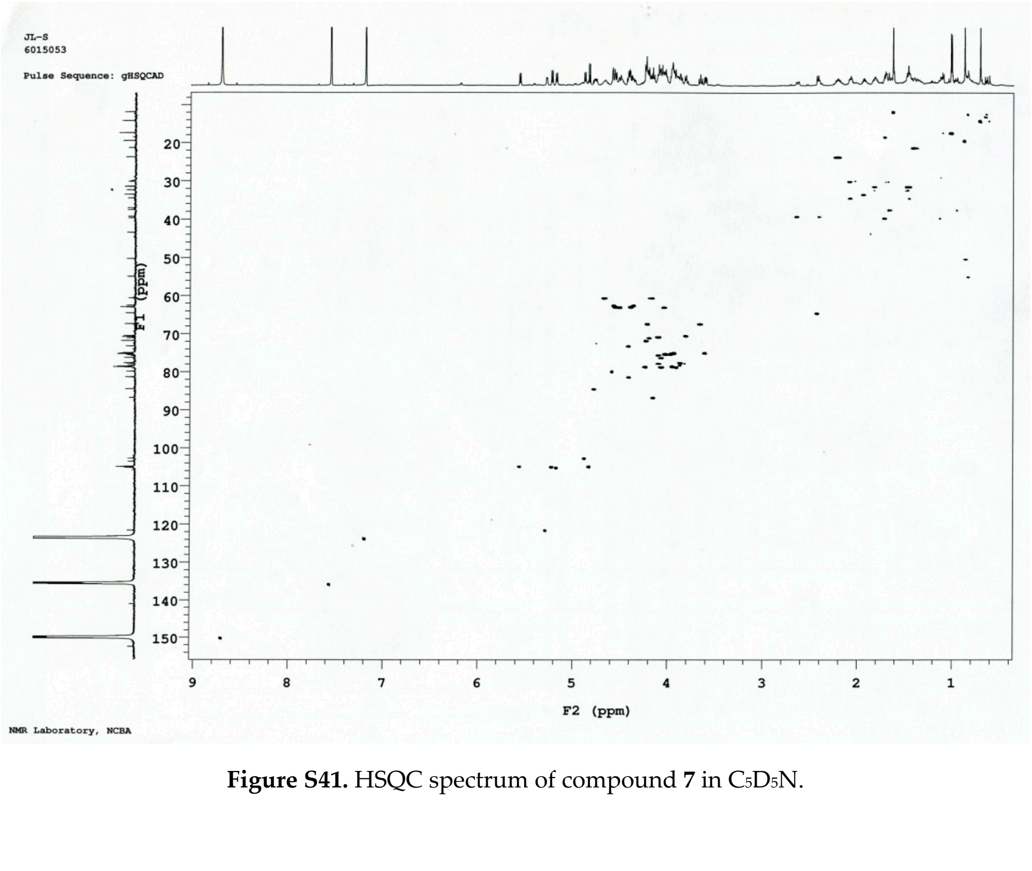

Figure S41. HSQC spectrum of compound **7** in C_5D_5N.

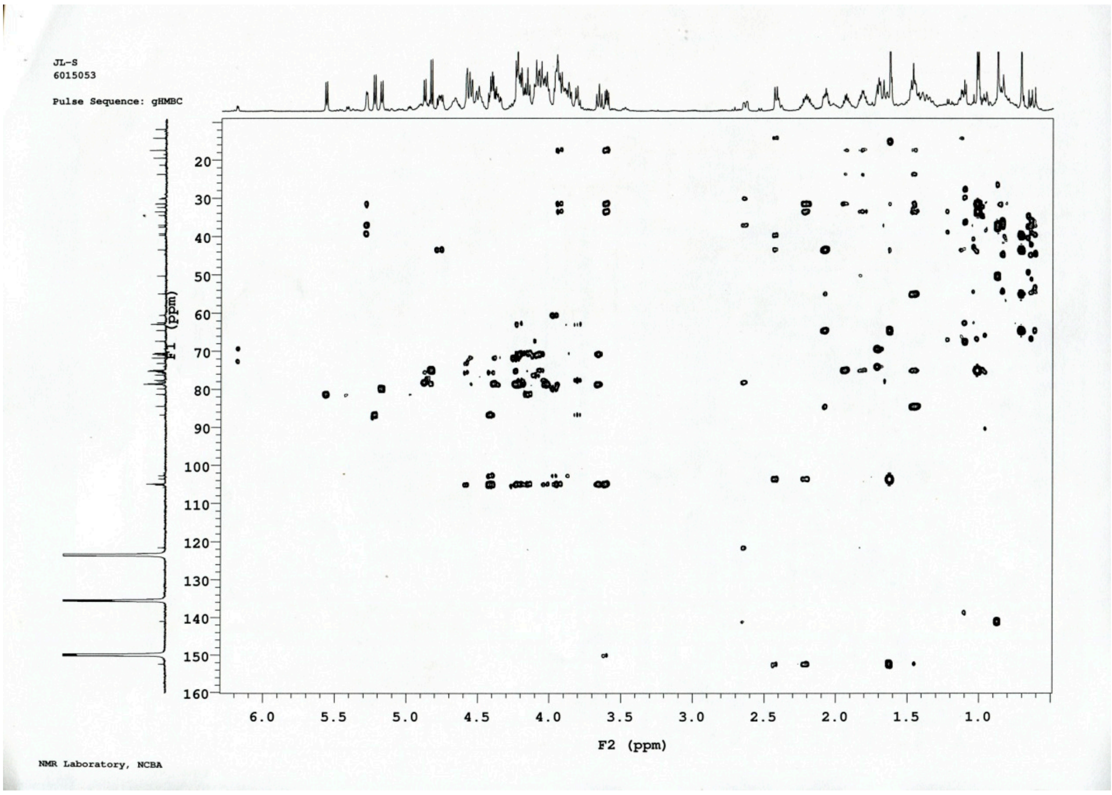

Figure S42. HMBC spectrum of compound **7** in C_5D_5N.

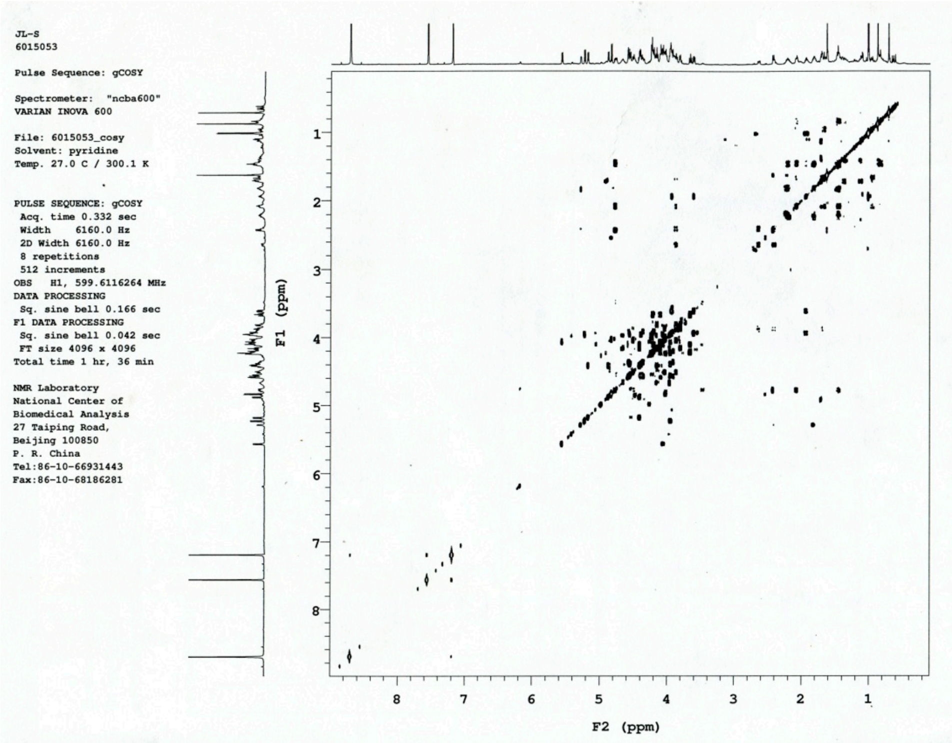

Figure S43. ^1H-^1H COSY spectrum of compound **7** in C_5D_5N.

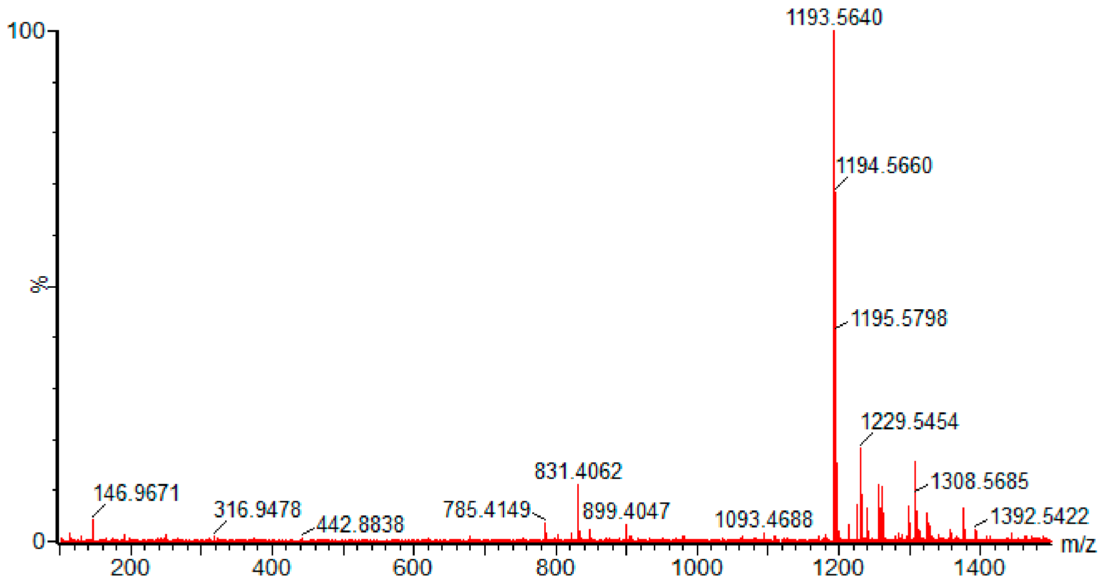

Figure S44. HRESIMS of compound **7** in CH_3CN.

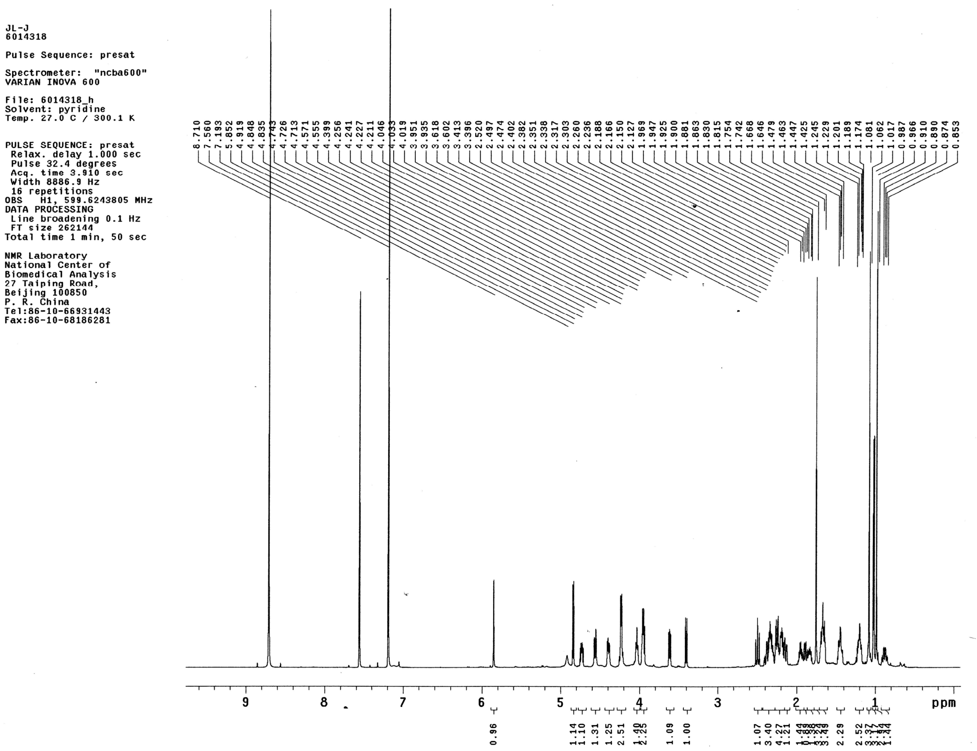

Figure S45. ^{1}H-NMR spectrum of compound **8** in C_5D_5N.

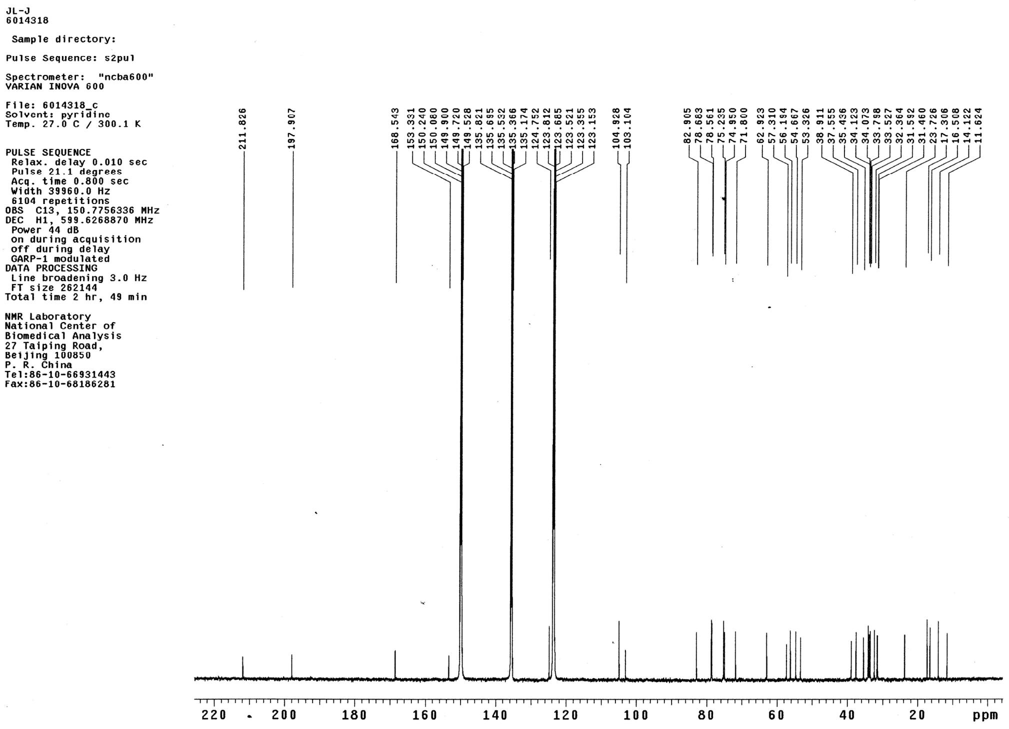

Figure S46. ^{13}C-NMR spectrum of compound **8** in C_5D_5N.

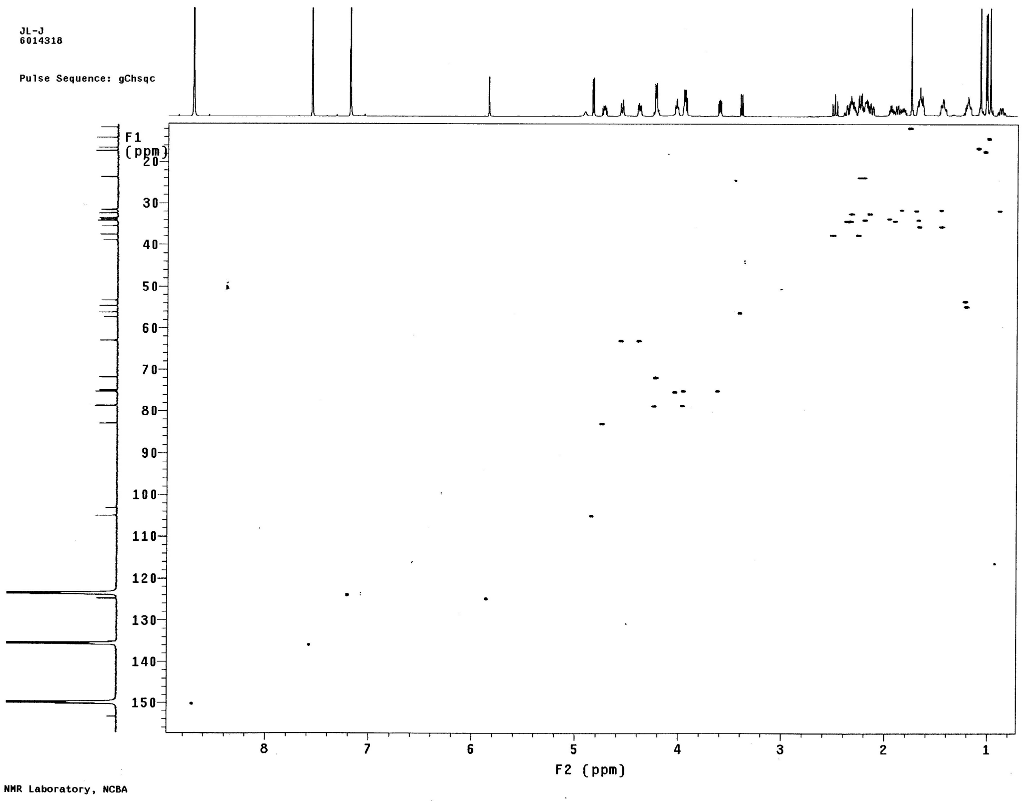

Figure S47. HSQC spectrum of compound **8** in C_5D_5N.

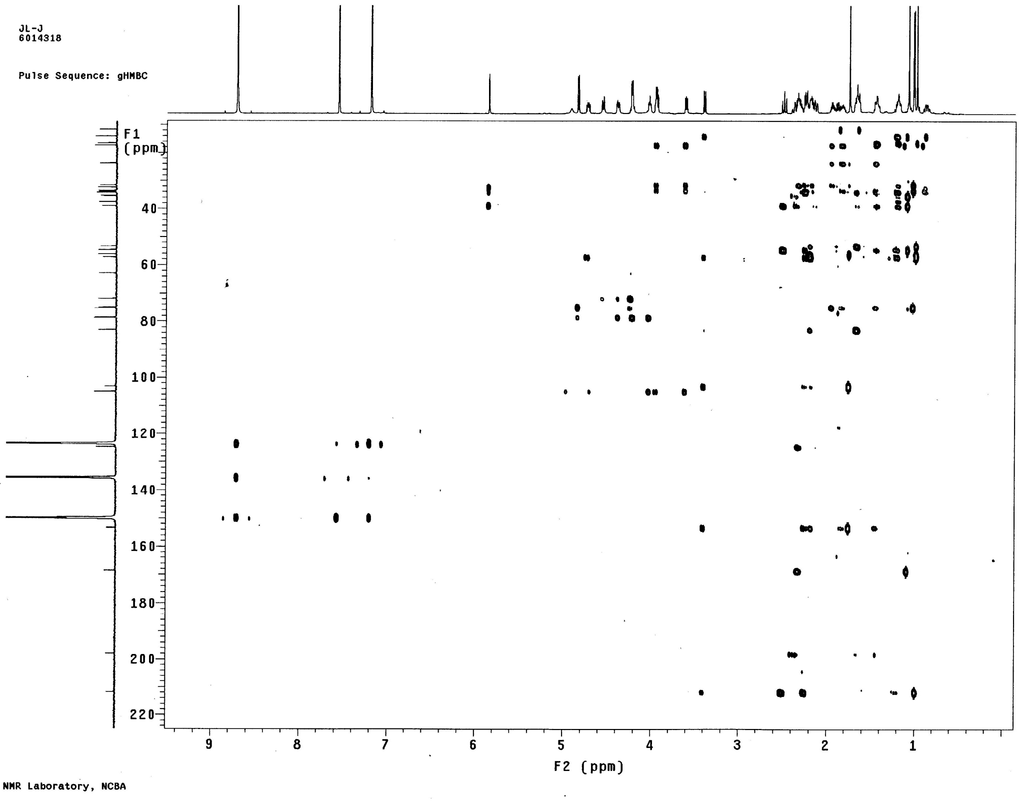

Figure S48. HMBC spectrum of compound **8** in C_5D_5N.

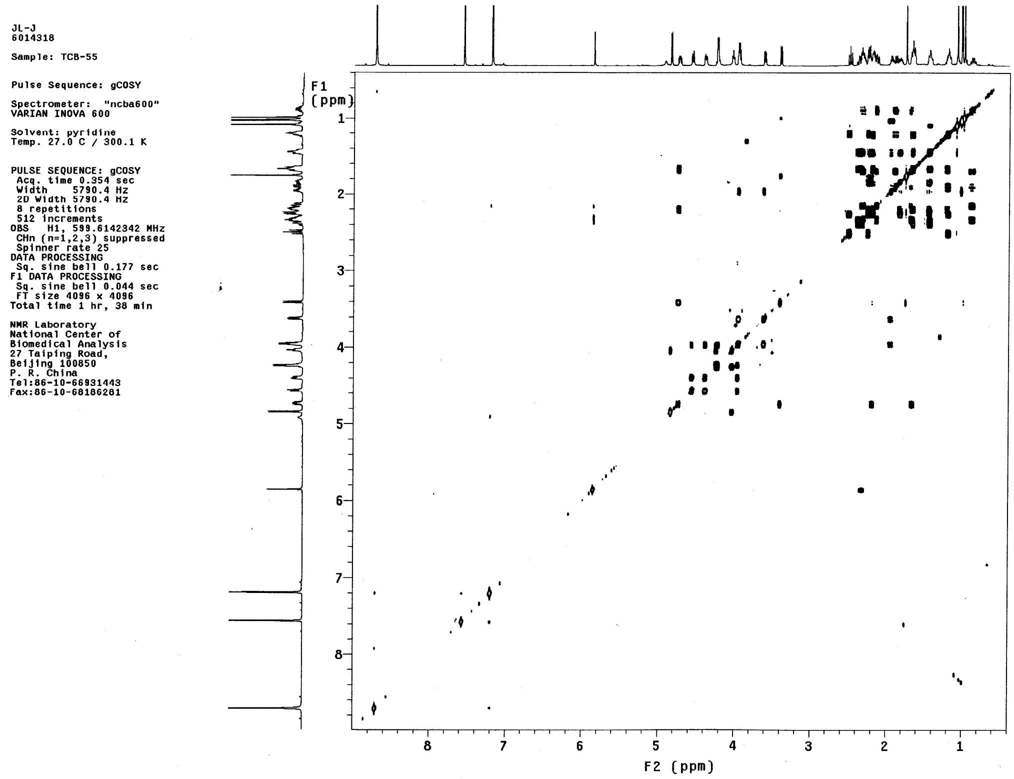

Figure S49. ^{1}H-^{1}H COSY spectrum of compound **8** in C_5D_5N.

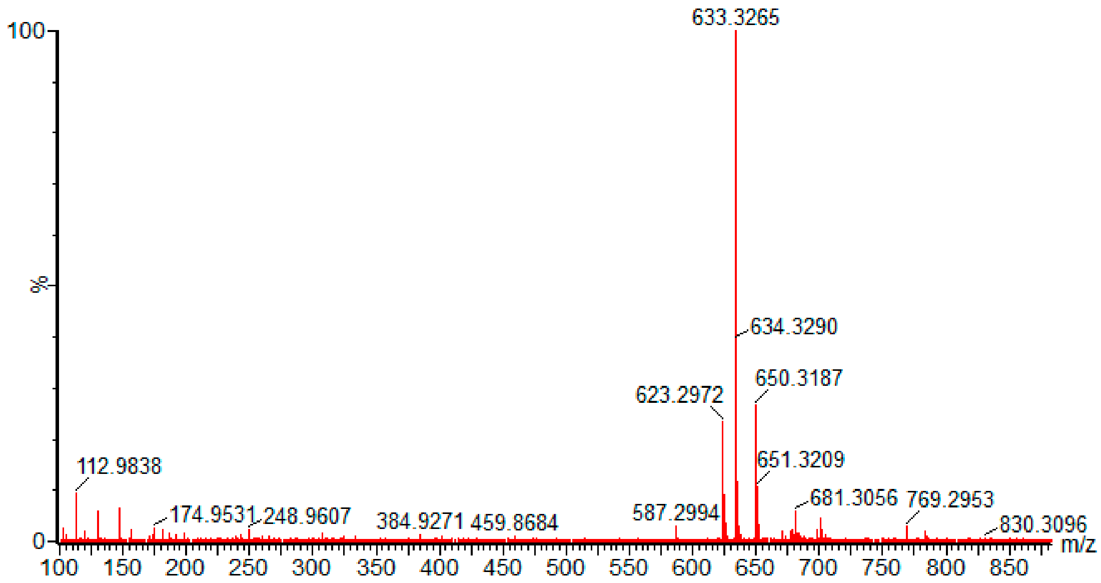

Figure S50. HRESIMS of compound **8** in CH_3CN.

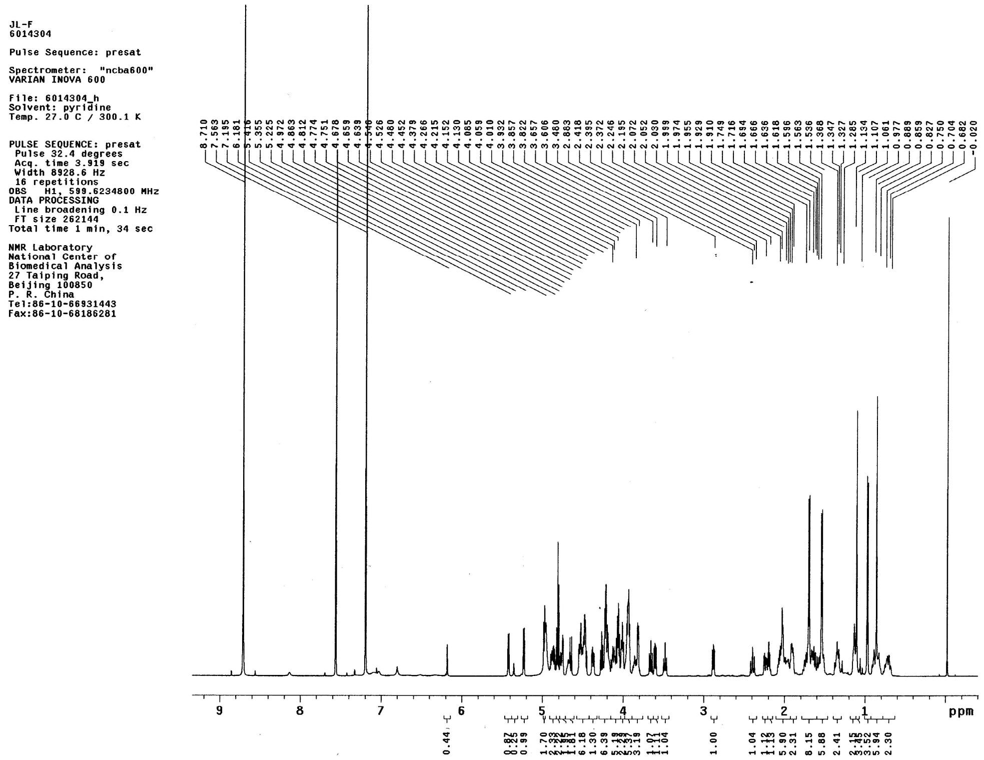

Figure S51. ^{1}H-NMR spectrum of compound **9** in C_5D_5N.

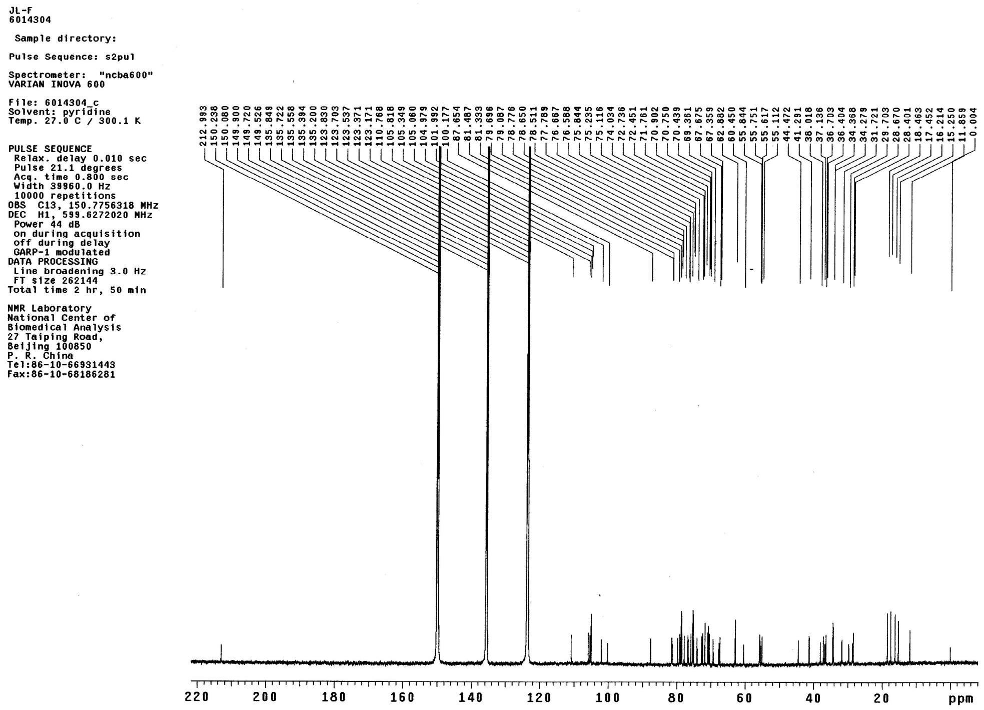

Figure S52. ^{13}C-NMR spectrum of compound **9** in C_5D_5N.

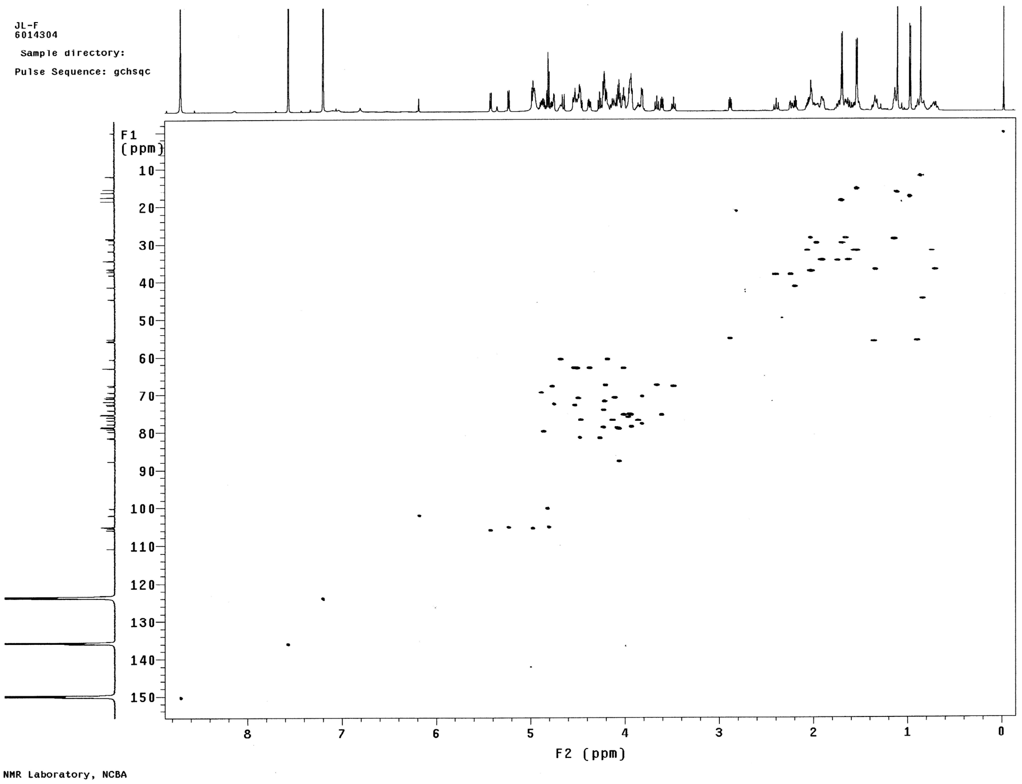

Figure S53. HSQC spectrum of compound **9** in C_5D_5N.

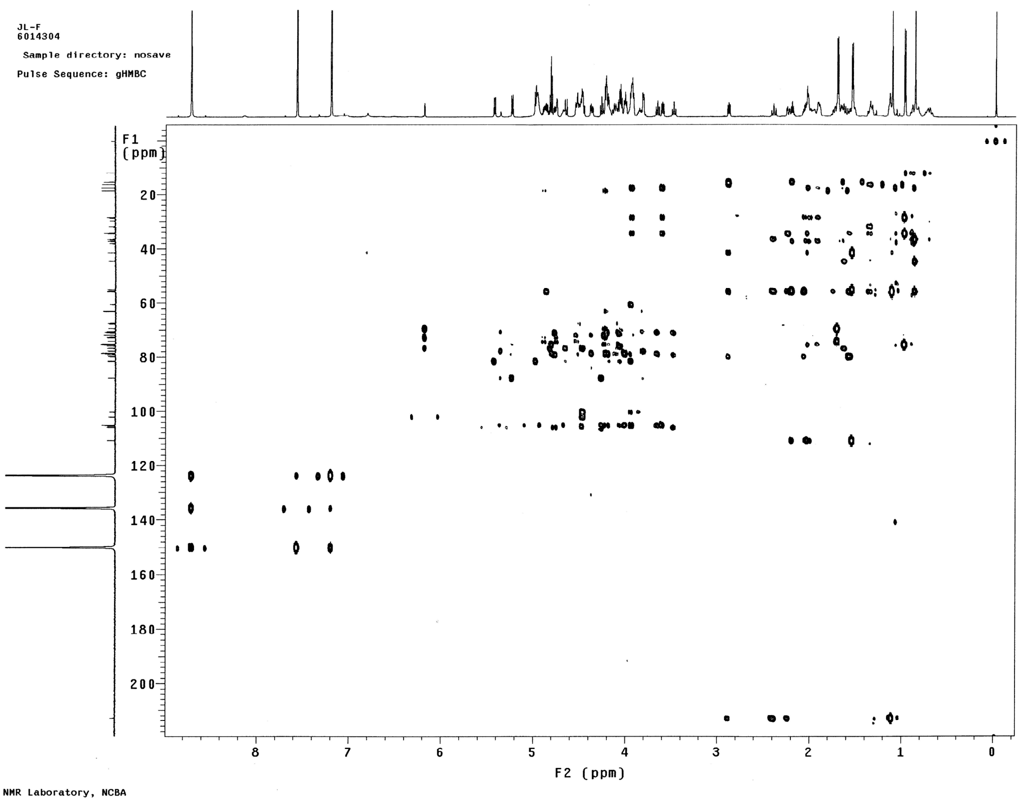

Figure S54. HMBC spectrum of compound **9** in C_5D_5N.

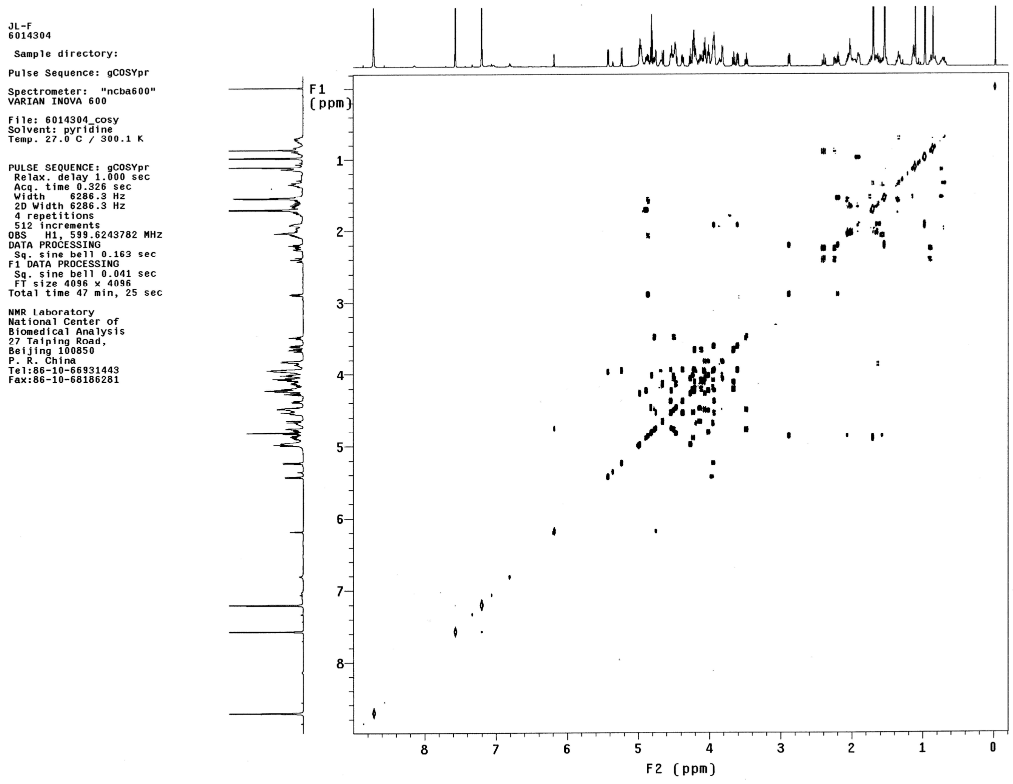

Figure S55. ^1H-^1H COSY spectrum of compound **9** in C_5D_5N.

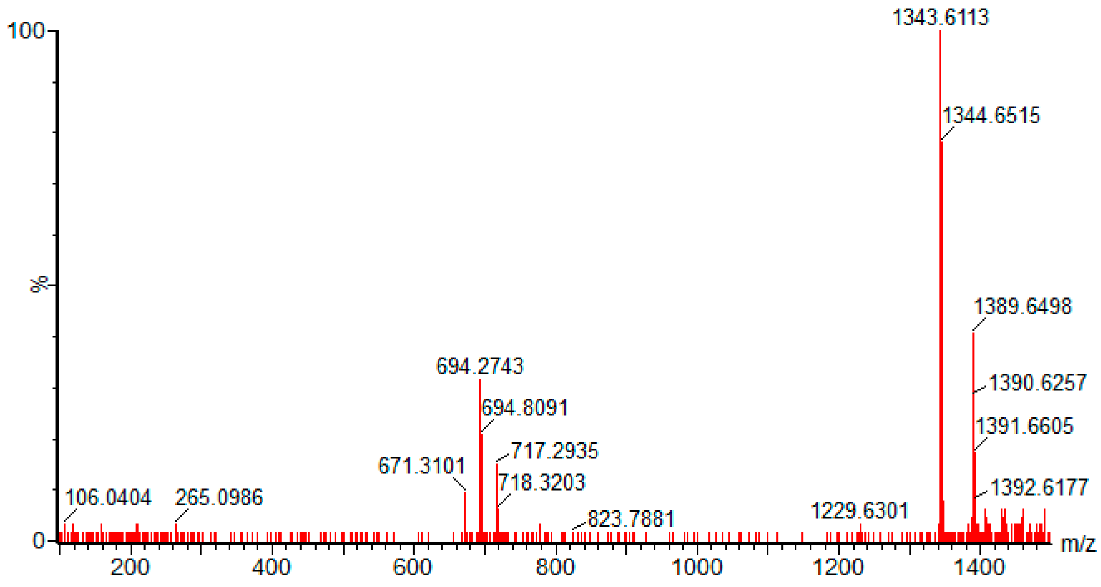

Figure S56. HRESIMS of compound **9** in CH_3CN.

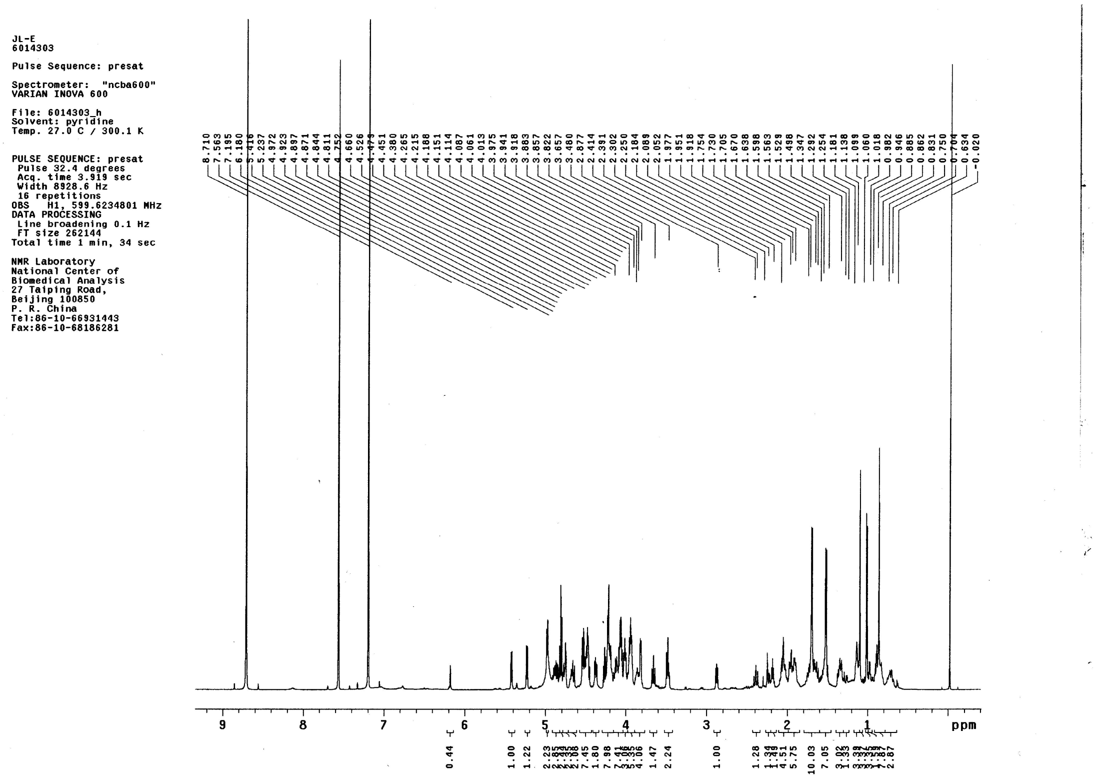

Figure S57. ^{1}H-NMR spectrum of compound **10** in C_5D_5N.

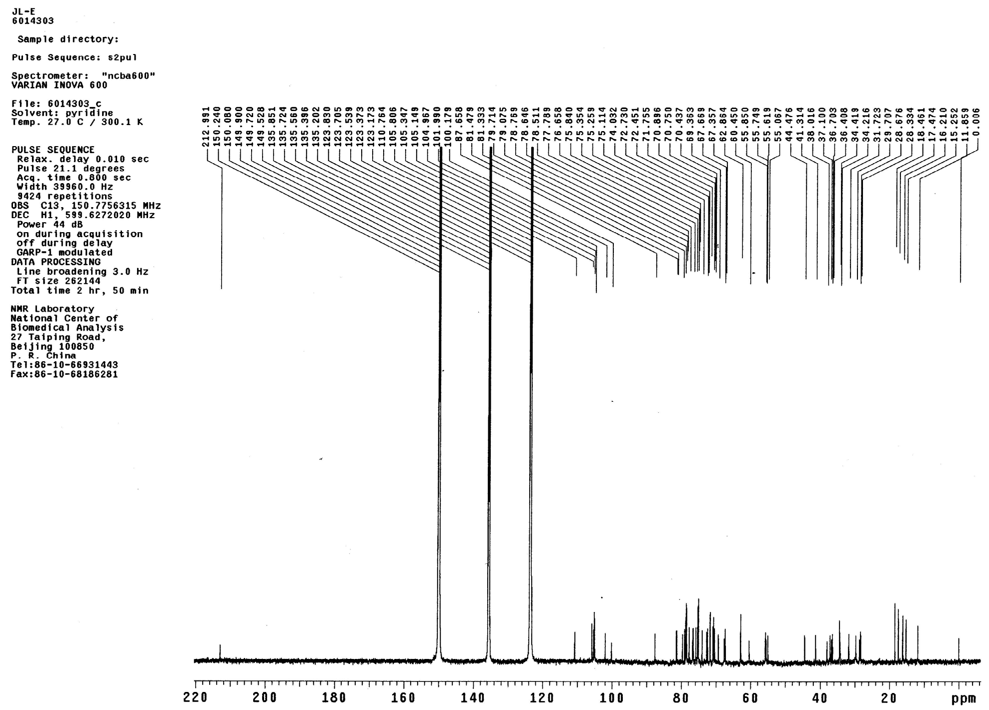

Figure S58. ^{13}C-NMR spectrum of compound **10** in C_5D_5N.

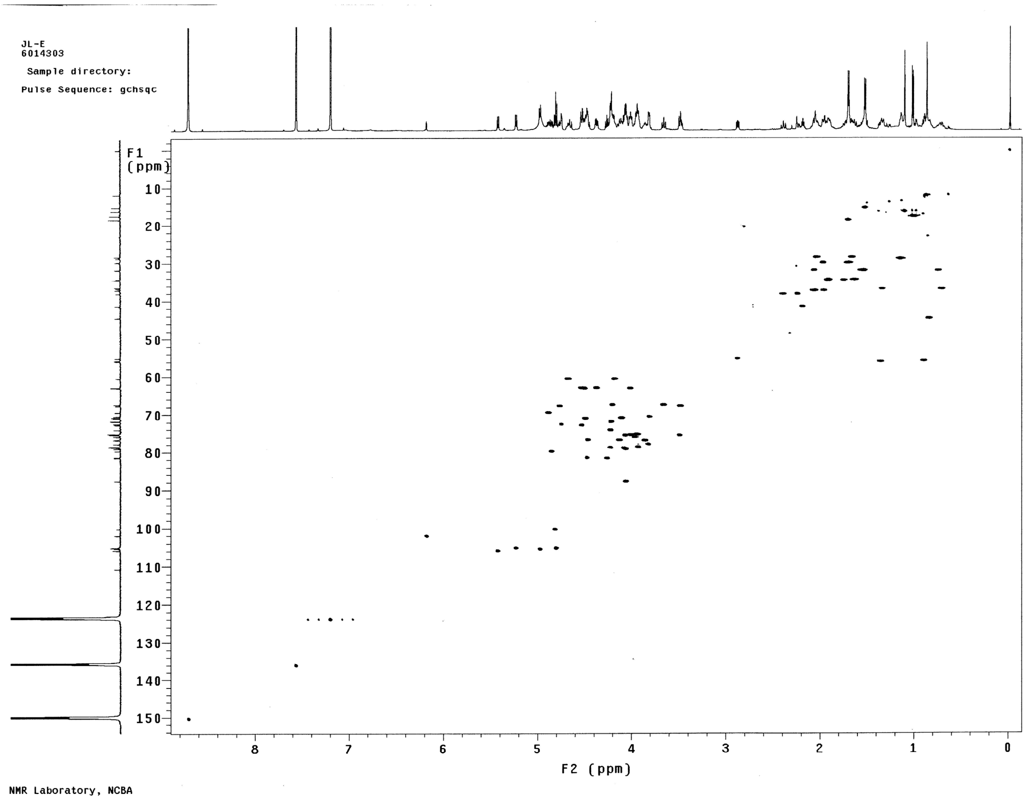

Figure S59. HSQC spectrum of compound **10** in C_5D_5N.

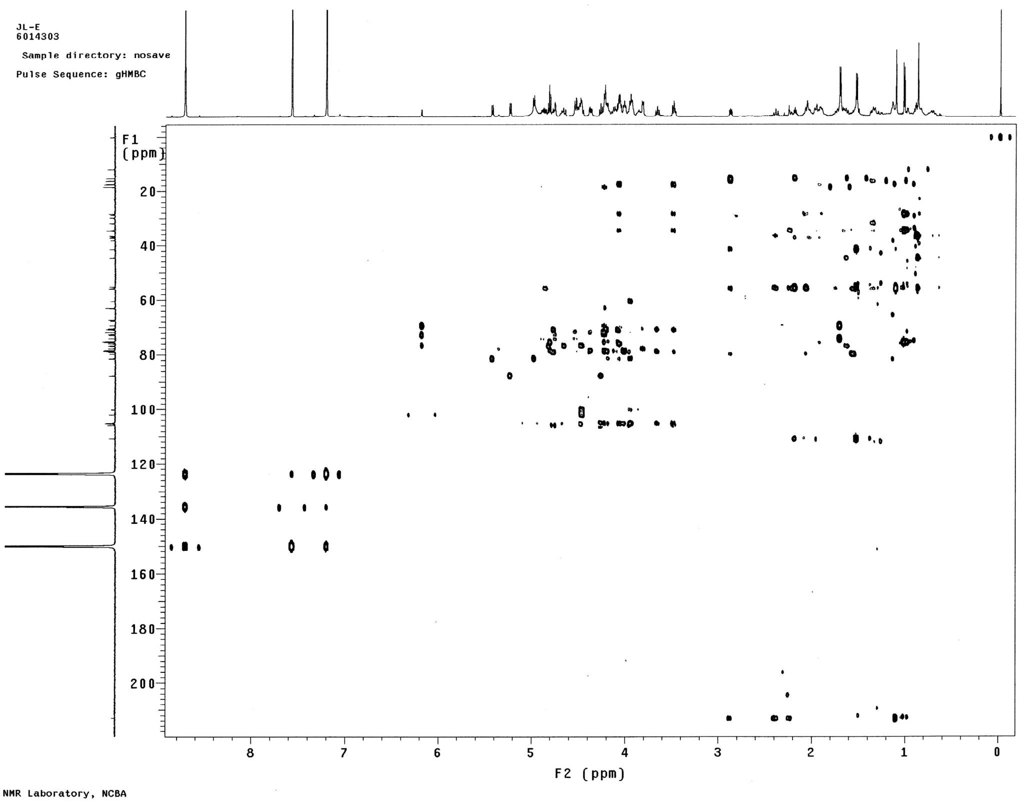

Figure S60. HMBC spectrum of compound **10** in C_5D_5N.

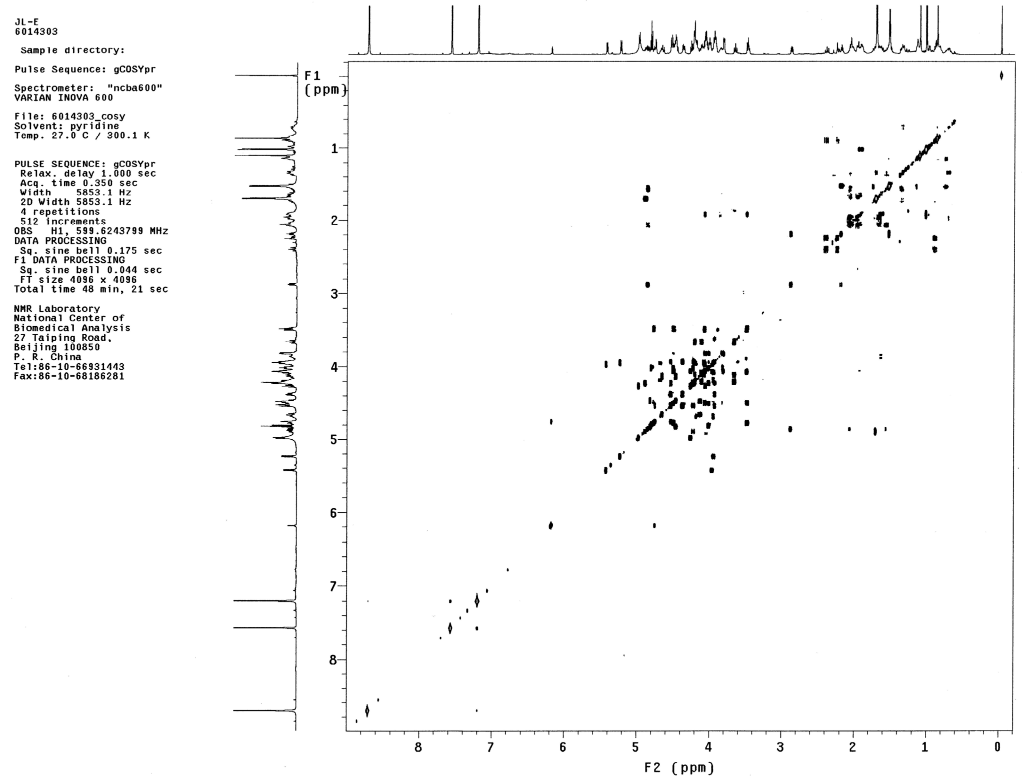

Figure S61. ^1H-^1H COSY spectrum of compound **10** in C_5D_5N.

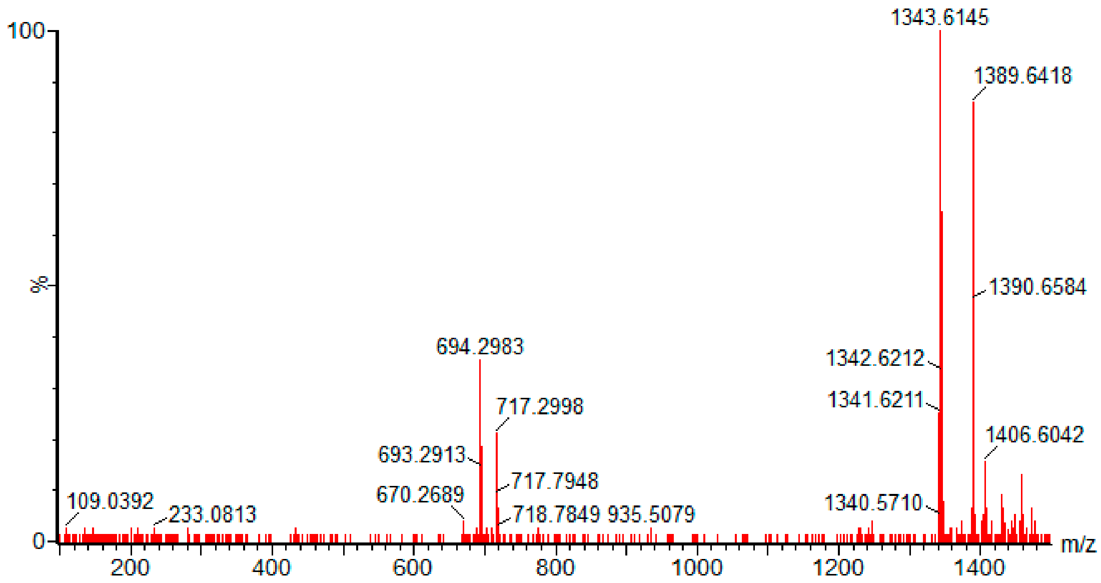

Figure S62. HRESIMS of compound **10** in CH_3CN.

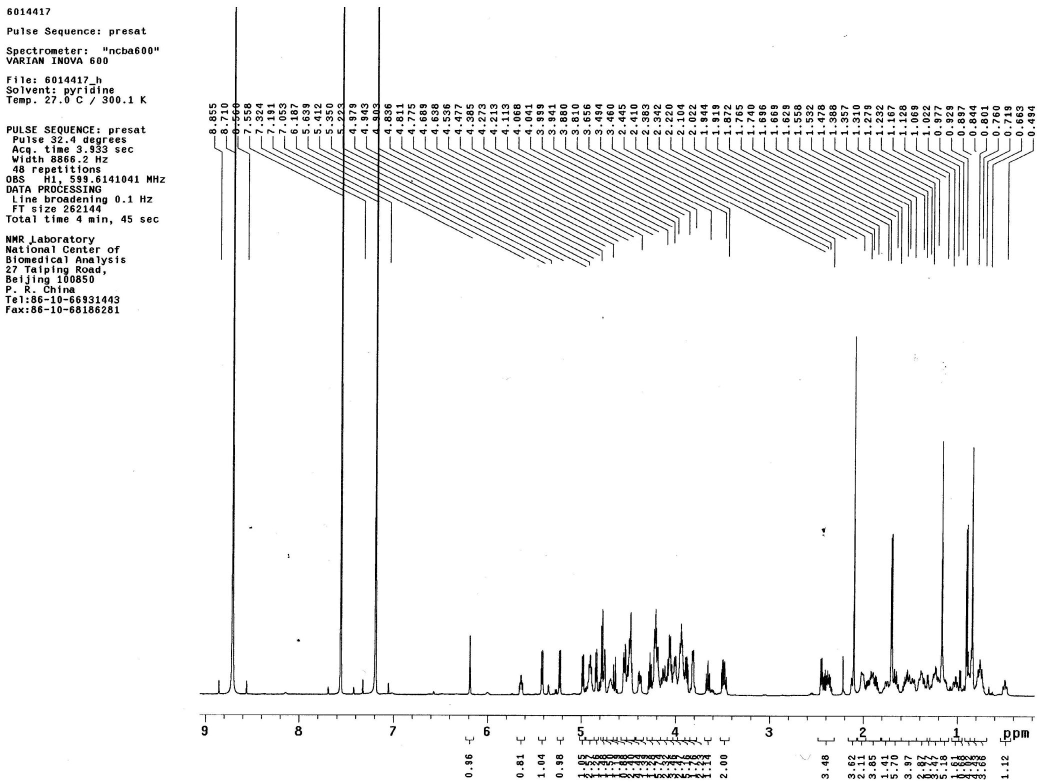

Figure S63. ^{1}H-NMR spectrum of compound **11** in C_5D_5N.

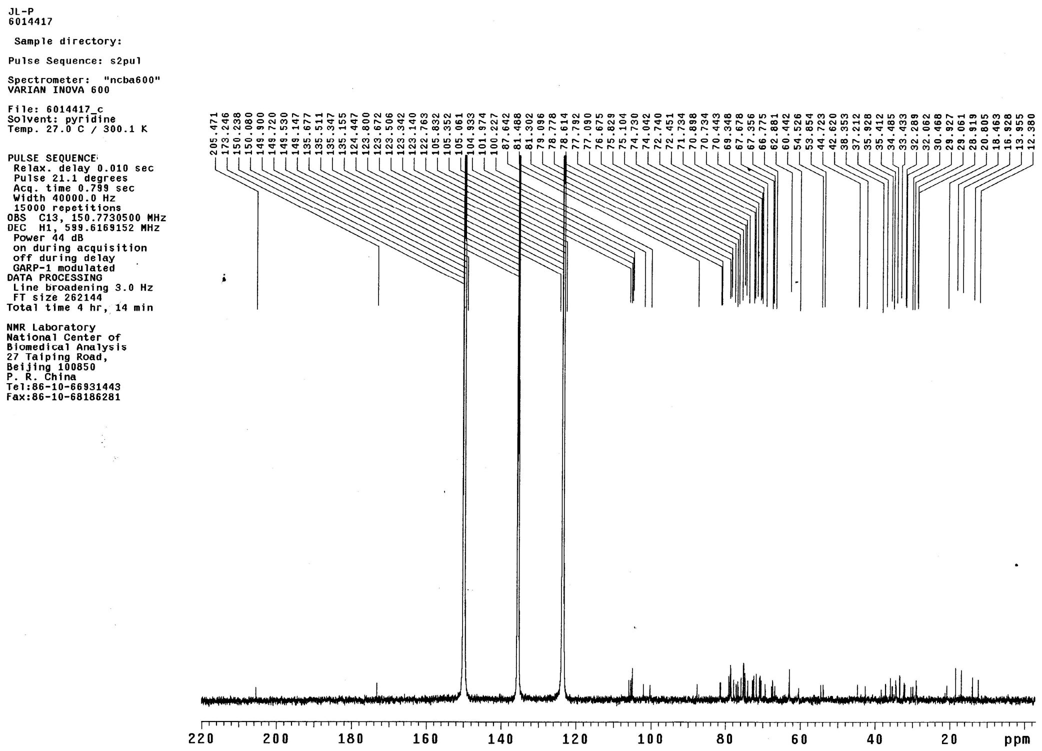

Figure S64. ^{13}C-NMR spectrum of compound **11** in C_5D_5N.

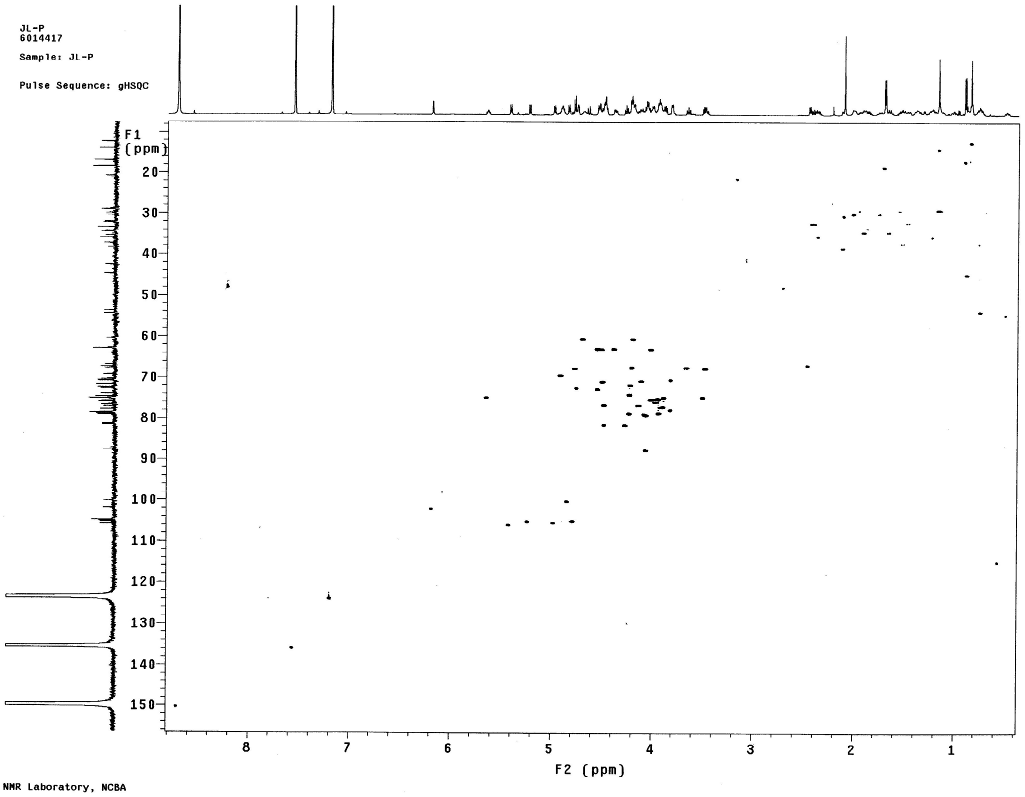

Figure S65. HSQC spectrum of compound **11** in C_5D_5N.

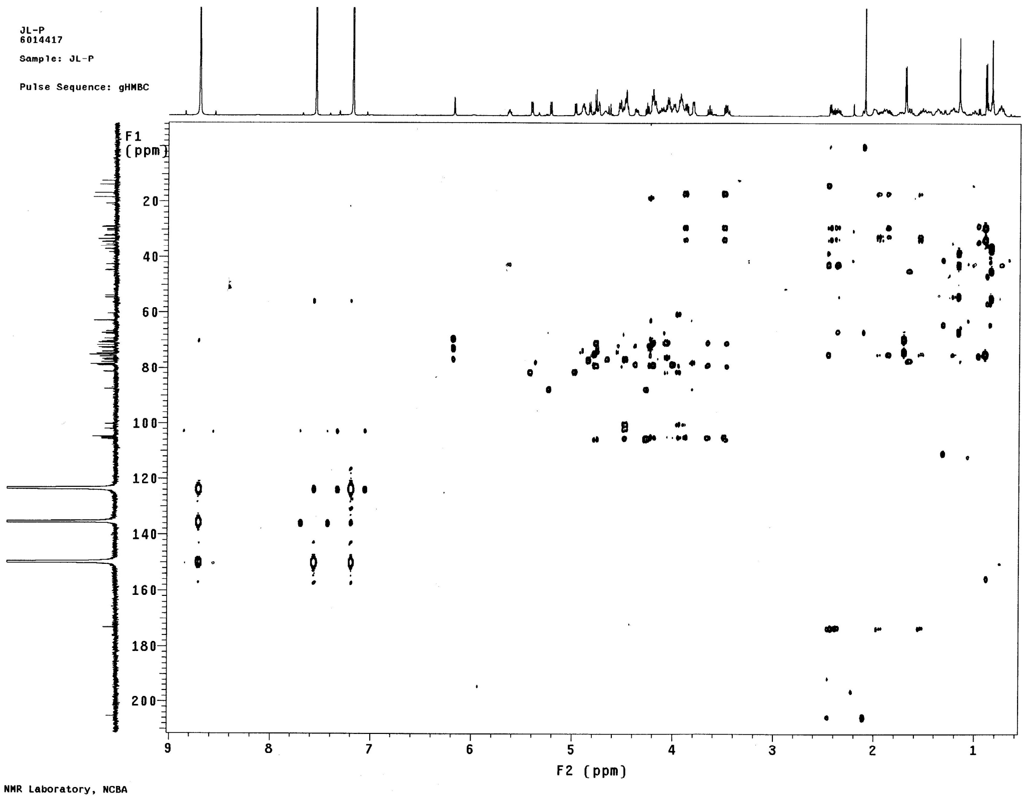

Figure S66. HMBC spectrum of compound **11** in C_5D_5N.

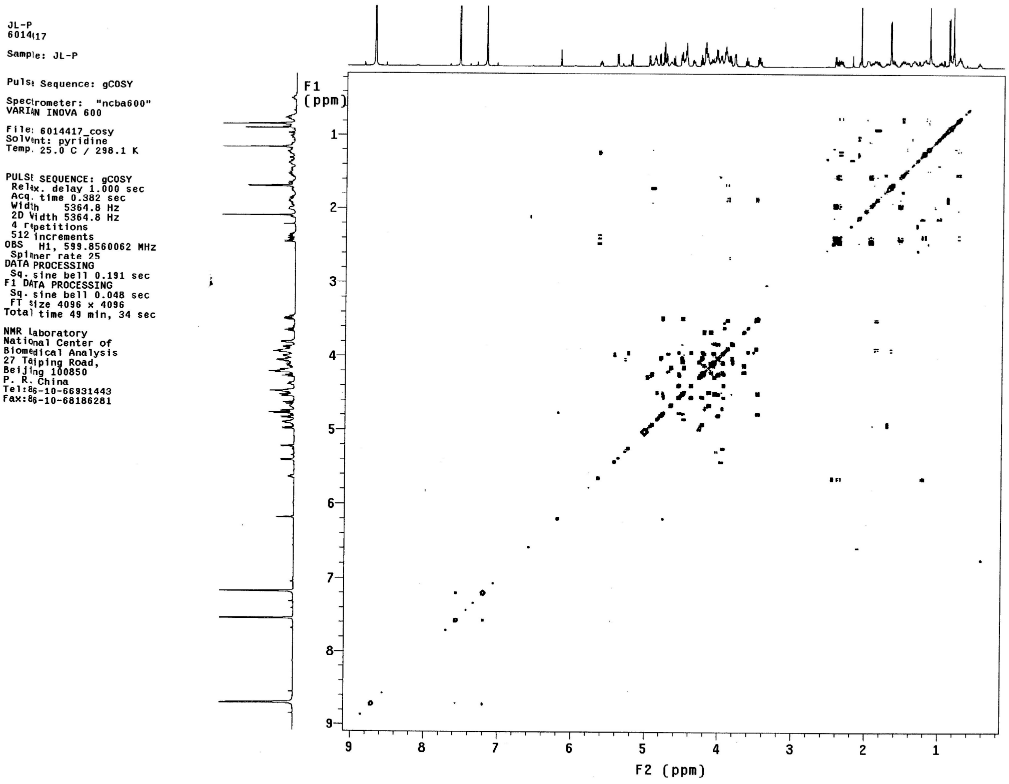

Figure S67. ^1H-^1H COSY spectrum of compound **11** in C_5D_5N.

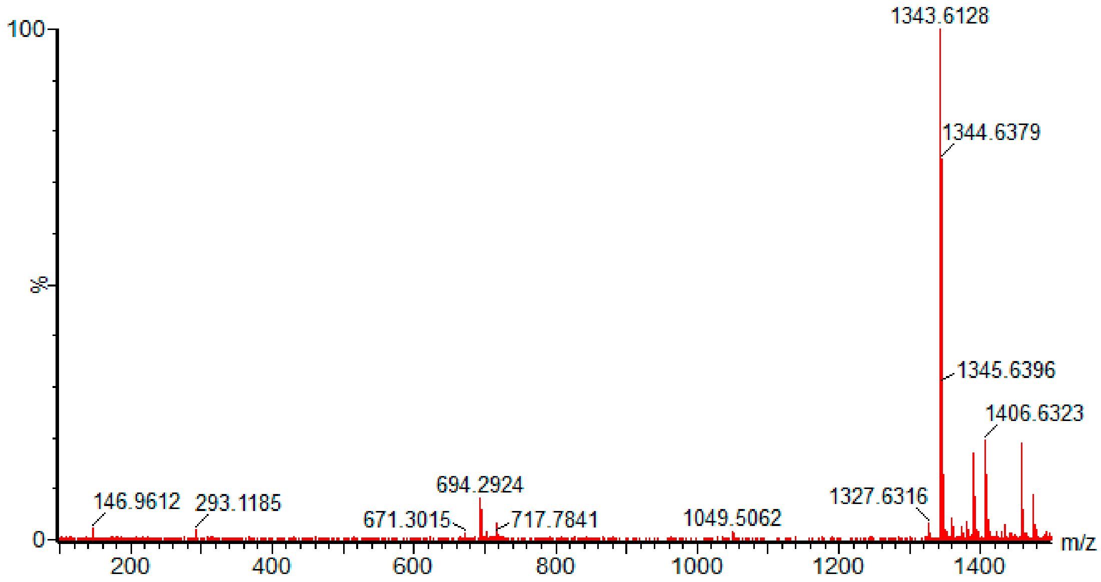

Figure S68. HRESIMS of compound **11** in CH_3CN.

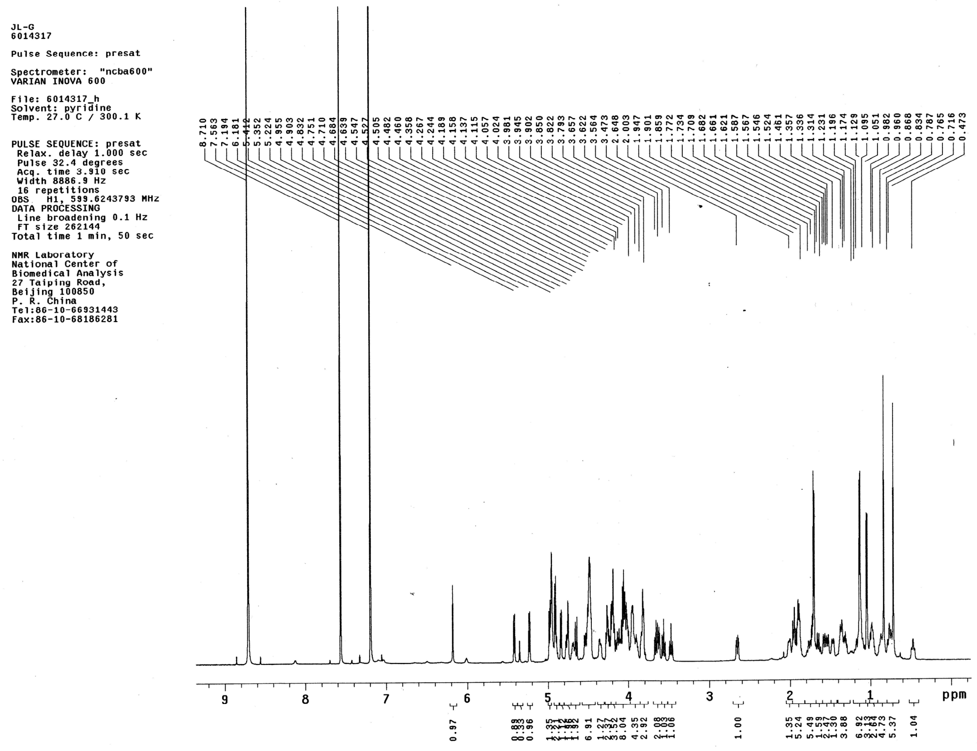

Figure S69. ^{1}H-NMR spectrum of compound **12** in C_5D_5N.

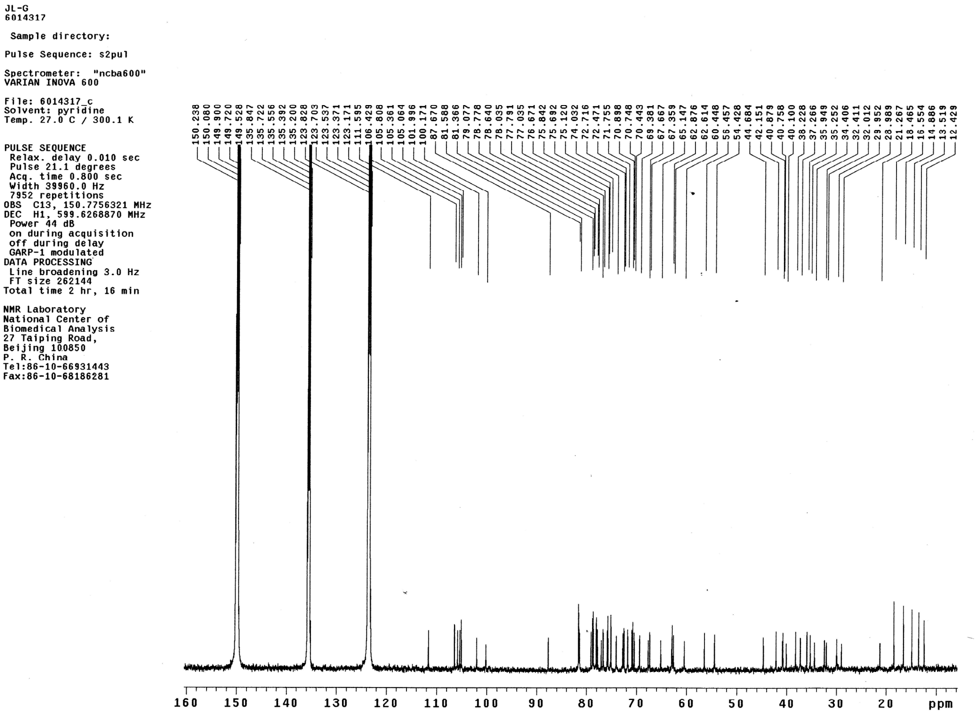

Figure S70. ^{13}C-NMR spectrum of compound **12** in C_5D_5N.

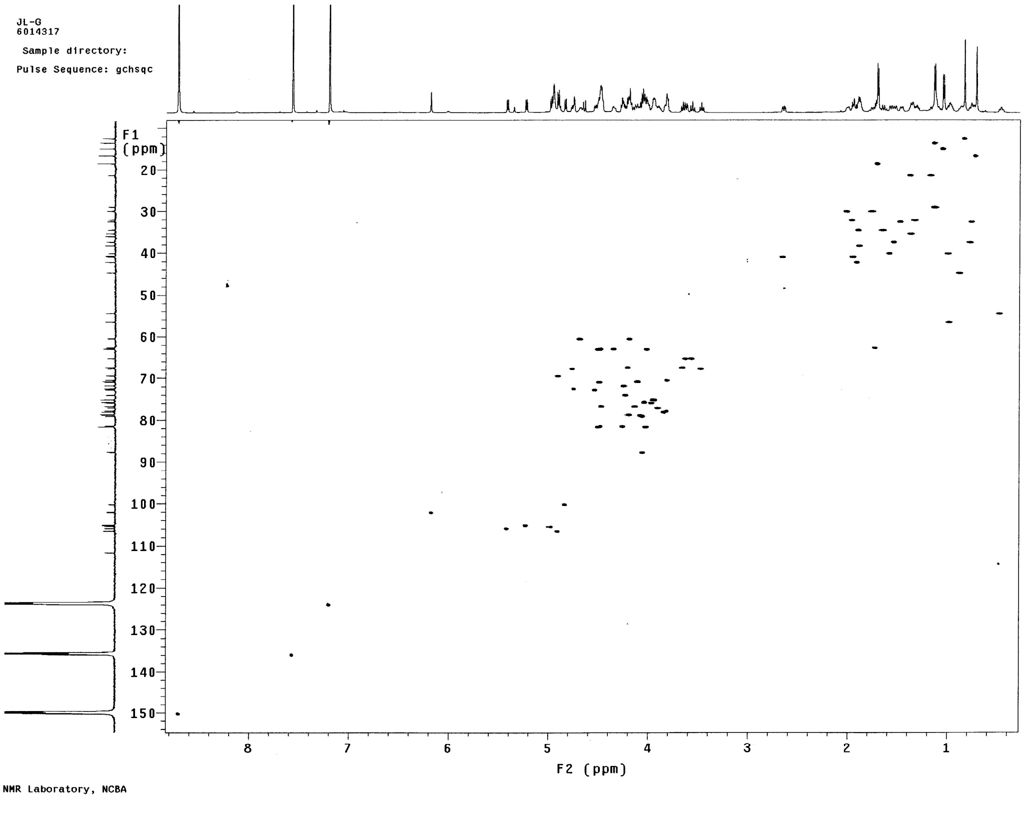

Figure S71. HSQC spectrum of compound **12** in C_5D_5N.

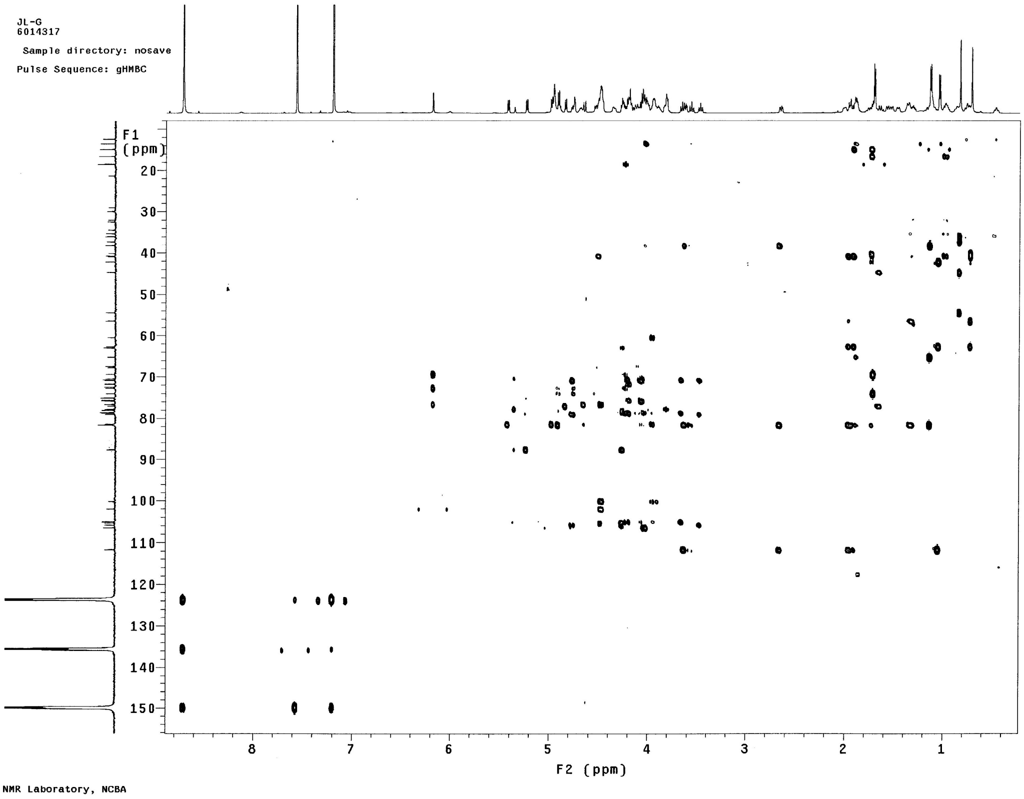

Figure S72. HMBC spectrum of compound **12** in C_5D_5N.

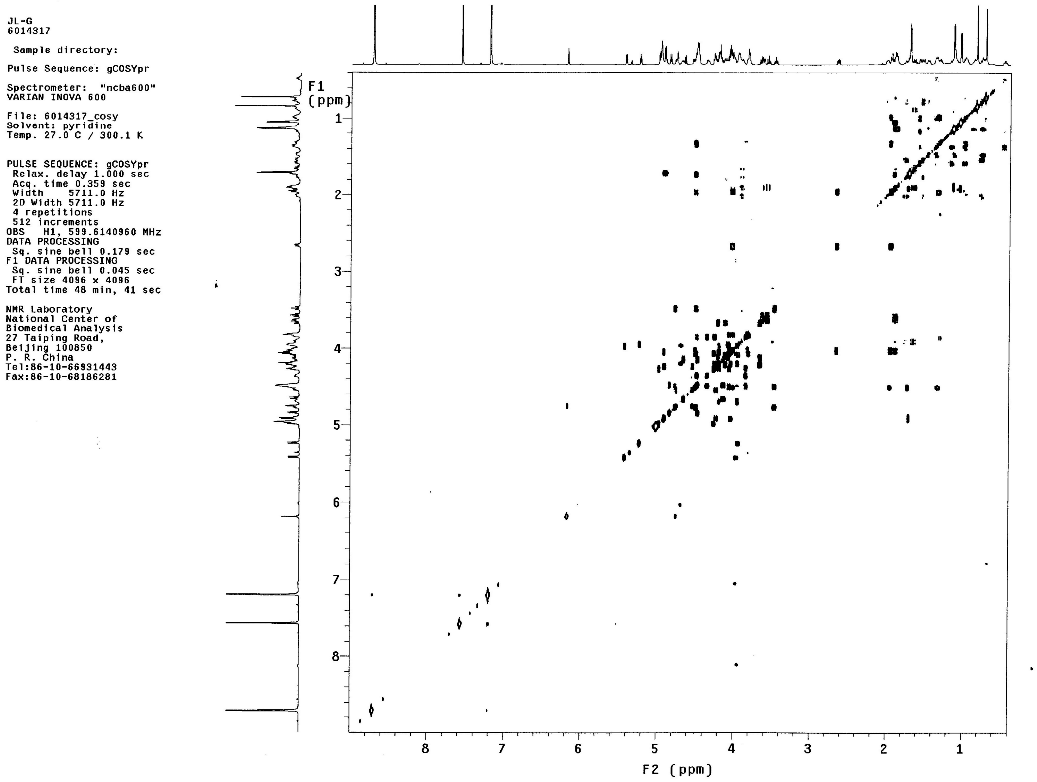

Figure S73. ^{1}H-^{1}H COSY spectrum of compound **12** in C_5D_5N.

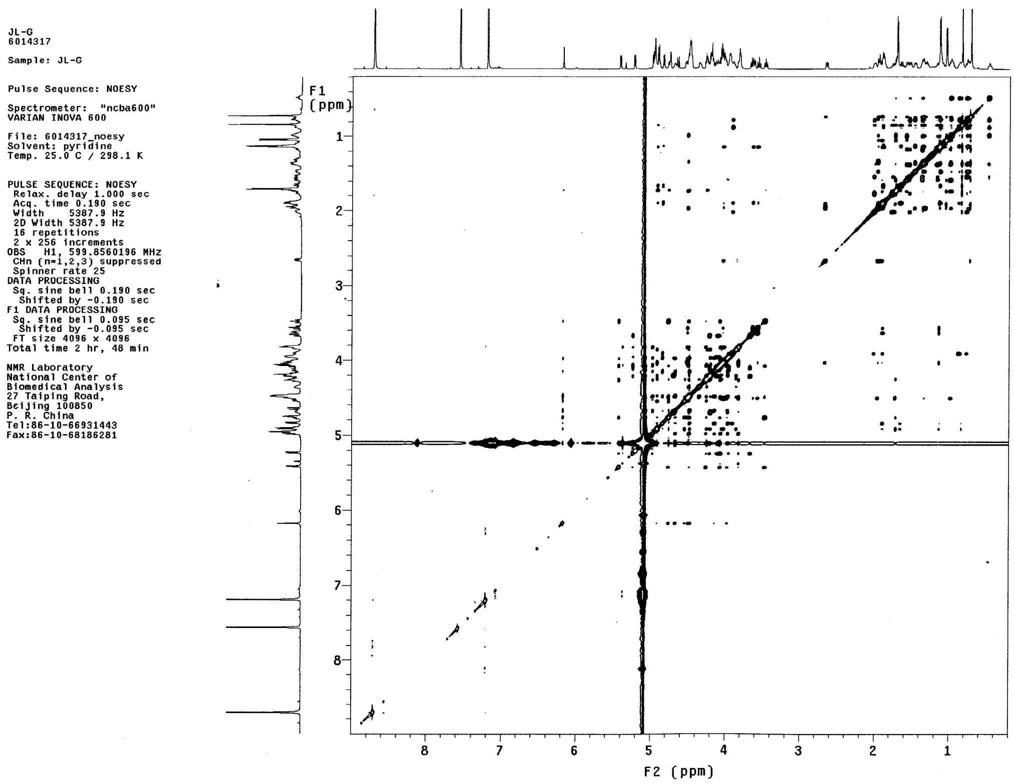

Figure S74. ROESY of compound **12** in C_5D_5N.

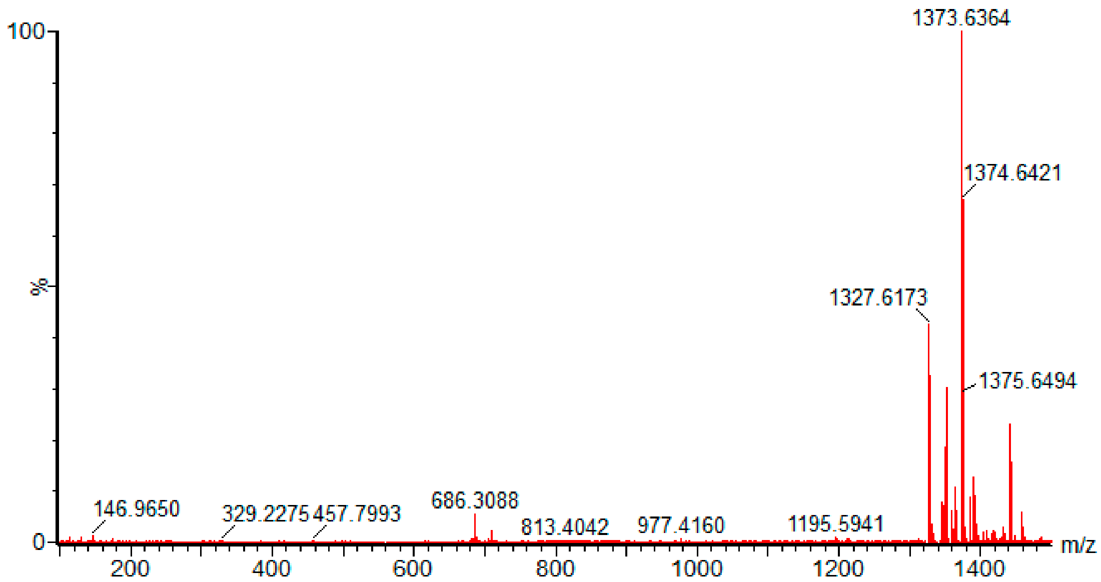

Figure S75. HRESIMS of compound **12** in CH_3CN.
